# Mediating Role of Internet Use in Cognitive-Depressive Pathways: A Random Intercept Cross-Lagged Panel Modeling Approach

**DOI:** 10.3389/ijph.2025.1608478

**Published:** 2025-10-21

**Authors:** Zhichao Wang, Zhongliang Zhou, Jiao Lu, Xinyue Zhang, Xiaohui Zhai, Yan Zhuang

**Affiliations:** ^1^ School of Public Policy and Administration, Xi’an Jiaotong University, Xi’an, China; ^2^ School of Public Health, Health Science Center, Xi’an Jiaotong University, Xi’an, China

**Keywords:** cognitive function, depression, internet use, longitudinal study, mediation analysis, Random Intercept Cross-Lagged Panel Model

## Abstract

**Objectives:**

Prior work has identified an inverse relationship between depression and cognition in older adults, but the mechanisms underlying this association remain unclear. This study investigated whether internet use mediates this relationship in middle-aged and older adults.

**Methods:**

Data were drawn from the China Health and Retirement Longitudinal Study (CHARLS) from 2015 to 2020 (n = 9,610). The Random Intercept Cross-Lagged Panel Model (RI-CLPM) with mediation analysis was used; subgroup analyses were conducted for middle-aged (45–64) and older (65+) adults.

**Results:**

At the between-person level, a significant negative correlation was found between cognitive function and depressive symptoms. Within-person analysis revealed a bidirectional relationship: poorer cognitive function predicted increased depressive symptoms (β* = −0.080, p < 0.001), and conversely, increased depressive symptoms predicted poorer cognitive function (β* = −0.019, p < 0.05). Internet use partially mediated this relationship, accounting for 8.58% and 9.69% of the total effects, respectively. This mediating effect was stronger in middle-aged adults than in older adults.

**Conclusion:**

These results emphasize the continued importance of exploring multidisciplinary interventions to mitigate depressive symptoms and delay cognitive decline in middle-aged and older adult populations.

## Introduction

As the global population ages, the increasing prevalence of age-related conditions like depression and cognitive decline has become a major public health challenge. Although these disorders do not affect all individuals equally, they can substantially compromise quality of life for those affected. At the societal level, these conditions contribute to rising healthcare costs, diminished workforce productivity, and an escalating demand for long-term care services. Collectively, these factors threaten to undermine economic stability and overwhelm social welfare systems in aging societies [1]. Depression accounts for a substantial portion of the global burden of disease, potentially leading to reduced physical activity, diminished social interaction, and lower quality of life [2, 3]. The World Health Organization reported approximately 970 million people worldwide were affected by mental disorders in 2019, and the COVID-19 pandemic dramatically worsened this situation [4]. Studies estimate that depression affects 28.4%–35.1% of people aged over 60 years, with higher prevalence in low- and middle-income countries [5]. Depressive symptoms are also prevalent in China, a middle-income country with a rapidly aging population [6]. Cognitive decline, including mild cognitive impairment, also climbs with age [7]. Additionally, depressive symptoms and cognitive decline frequently co-occur in older adults [8], with depression often present in those with cognitive impairment [9]. Elucidating the intricate relationship between cognitive decline and depressive symptoms, as well as any mediating factors, is an urgent priority in geriatric research.

Several studies have explored the association between depression and cognition, with findings generally falling into three categories. First, they appear to be bidirectionally linked. Meta-analyse show a significant association between depression severity and cognitive impairments (including executive function, memory, and processing speed) [10]. Bennett and Thomas [11] posited that early-life depression may increase later dementia risk, and *vice versa* [11]. A recent 16-year study in the UK demonstrated that severe depressive symptoms predict accelerated memory decline [12]. From the scarring hypothesis perspective, depression triggers chronic physiological and neurochemical changes that impair cognitive function, resulting in long-term cognitive deficits [13]. Conversely, cognitive impairment can worsen depression by hindering self-regulation, communication, and social engagement [14]. Second, cognitive function can predict depression, but the reverse may not hold. Aichele and Ghisletta [15] found that delayed memory recall predicted future depression, but this effect lessened with age [15]. However, their study did not show that prior depression caused a decline in memory recall. Likewise, a study of older Americans revealed that higher baseline depression levels predicted future declines in episodic memory, but not the reverse [16]. Third, prior research, such as the study by Gale et al. [17], has indicated that depression does not predict cognitive impairment [17], suggesting that the two conditions are not necessarily causally linked. Previous studies have identified mechanisms linking depression and cognitive decline in older adults, including behaviors like physical inactivity, smoking, and social withdrawal, as well as mediators such as emotion regulation and sleep quality [15, 18, 19].

The rapid advancement of artificial intelligence and digital technologies has significantly increased internet adoption among Chinese middle-aged and older adults. According to official statistics, national internet penetration rates rose from 50.3% to 70.4% between 2015 and 2020, with the 50-and-older demographic exhibiting particularly dramatic growth (from 9.2% to 26.3%) [20]. The expansion of internet applications has facilitated online health services, such as medication ordering and online medical consultations, potentially enabling better self-management of health in middle-aged and older adults. In fact, previous multinational studies have demonstrated internet use’s protective effects on cognitive function [21], general healthy status [22], and psychological health [23]. While online activity necessitates some level of cognitive ability, cross-cultural evidence from North America, Europe, and East Asia suggests internet use may also enhance cognitive capacity [24] and mitigating overall cognitive decline [21, 22]. For instance, internet use may offer non-pharmacological relief from cognitive decline through cognitive stimulation and social engagement [21]. Online social activities (e.g., chats, social networking) may enhance cognitive function in seniors by promoting neuroplasticity and social engagement [25], similar to the cognitive stimulation that builds resistance to age-related brain changes [26]. Additionally, active internet users can effectively stay in touch with friends and expand their social networks, and this could reduce loneliness and depression, especially for older adults [27]. A longitudinal study of Americans over 50 found that internet use reduced depression risk by one-third [28], while Chinese cross-sectional studies have linked moderate internet use to positive outcomes for older adults, including reduced depressive symptoms and improved cognition [29]. Given that many digital tools, such as online chats and games, can affect both cognitive function and depression in this demographic, and although preferred platforms differ regionally [23, 24, 30], there may be a potential relationship between cognition, depression, and internet use. Additionally, lack of social contact and recreation increases the risk of both cognitive impairment and depression, with social activities acting as a crucial link [15, 27]. Importantly, online interaction and entertainment can provide this social engagement, improving psychological wellbeing through social activities and community connection [21, 22]. Considering internet use as an alternative form of social engagement or leisure, we hypothesize that access to the internet may mediate the relationship between cognitive function and depression.

However, current research lacks clarity on how variations in internet use impact the relationship between cognitive function and depression across different age groups [31]. The digital divide perspective underscores this uncertainty, highlighting significant disparities in internet access and its subsequent cognitive and mental health benefits between middle-aged and older adults, stemming from the latter’s limited resources and social capital [22, 32]. Furthermore, the reliance on cross-sectional and bilateral studies limits our ability to establish causality or examine trilateral associations [10, 29], and widely used longitudinal designs struggle to separate interpersonal and individual factors, hindering our understanding of the complex relationships and potentially causing biased estimates [33–35]. Therefore, we utilized the Random Intercept Cross-Lagged Panel Model (RI-CLPM), a type of structural equation modeling (SEM) [36], to disentangle within-person changes from between-person differences, capture dynamic reciprocal relationships, and enhance the rigor of causal inference [35].

This study aimed to address three key research questions: (1) What is the longitudinal association between cognitive function and depressive symptoms in middle-aged and older Chinese adults? (2) To what extent does internet use mediate this relationship? (3) Are there differences in these mediation effects between middle-aged and older populations? Using CHARLS 5-year longitudinal data and controlling for demographics [37], socioeconomic status [38], lifestyle behaviors [19], daily activities [39], and health conditions [14, 33], we systematically examined these relationships while also exploring differences in the mediating effect of internet use across different age groups.

## Methods

### Data and Sample

The data for this study were drawn from the China Health and Retirement Longitudinal Survey (CHARLS), a nationally representative longitudinal study designed to explore the multifaceted transitions associated with aging in China [40]. Managed by the National School for Development (China Center for Economic Research), CHARLS has been tracking a cohort of participants every 2 years since its inception in 2011, encompassing 28 provinces, 150 counties, and 450 communities. This survey focuses on individuals aged 45 and older and their spouses, employing structured face-to-face interviews to gather comprehensive data on their social, economic, and health profiles [8].

This study analyzed longitudinal data from Waves 3–5 (2015–2020) of the China Health and Retirement Longitudinal Study (CHARLS). The baseline sample in 2015 included 14,883 participants aged 45 years or older. Consistent with established methodologies for longitudinal cognitive research, we excluded participants meeting any of the following criteria: (1) missing baseline cognitive assessments (n = 2,163), (2) incomplete baseline depression measures (n = 473), or (3) loss to follow-up (n = 3,637). The final analytic cohort consisted of 9,610 participants with complete baseline data and at least one follow-up assessment. Supplementary Figure S1 in Supplementary Appendix A illustrates the detailed sample attrition process.

### Measures

#### Cognitive Function

Cognitive function encompasses diverse processes, including sustained attention, language processing, executive functions (e.g., problem-solving, cognitive flexibility), learning, and memory [41]. We assessed cognitive function using a validated Chinese Mini-Mental State Examination (mMMSE, 0–21 points) comprising three dimensions: orientation and calculation (TICS-10, 0–10), episodic memory (immediate and delayed word recall, 0–10), and visual construction (figure drawing, 0–1). Correct responses scored 1 (incorrect/don't know = 0), with higher scores indicating better cognition. This scale showed excellent reliability (Cronbach’s α = 0.81–0.83 across CHARLS waves) and discriminative validity for cognitive impairment and depression in Chinese populations [8, 42].

#### Depressive Symptoms

Depressive symptoms were assessed using the Center for Epidemiologic Studies Depression Scale (CESD-10) within the CHARLS dataset. The CESD-10 is a 10-item measure of depressive symptomatology, assesses self-reported experiences across domains including being bothered by little things, concentration difficulties, fatigue, depressed mood, hope, fearful, happiness, loneliness, sleep disturbance, and feelings of lack of purpose. Items utilize a 4-point Likert scale (0 = never, 1 = sometimes, 2 = often, 3 = most of the time), with negatively worded items reverse-scored prior to summation. The resulting total score (range 0–30) represents the severity of depressive symptoms, with higher scores indicating greater symptom burden. The CESD-10 scale demonstrated high reliability and validity in measuring depressive symptoms among psychological studies [8, 37]. The scale exhibited acceptable internal consistency across the three waves of data collection, as reflected by Cronbach’s α coefficients of 0.793, 0.803, and 0.840.

#### Internet Use

Internet use was measured dichotomously (1 = yes, 0 = no) using the question: “Have you used the internet in the past month, including activities such as mobile messaging, news browsing, video streaming, gaming, and online financial services?” Compared to the “past year” recall period commonly used in prior studies [43], our 1-month timeframe reduces memory bias and better captures recent usage patterns. While this operationalization effectively measures basic access (the first level of the digital divide) [44], we recognize its limitations in assessing usage frequency and purposes. The shorter recall period enhances measurement accuracy, though future research would benefit from incorporating more multidimensional usage indicators.

#### Control Variables

Drawing from prior research, we included the following covariates related to participants’ demographic and socioeconomic characteristics: gender, age, education, residence type, and marital status. Lifestyle-related covariates consisted of physical activity level, smoking habits, and alcohol consumption. Additionally, to control for the health status of middle-aged and older adults, we incorporated both the IADL scale and the cumulative chronic disease index (see Supplementary Appendix A Supplementary Table S1 for details) as covariates.

#### Statistic Analysis

The RI-CLPM was used to estimate the direction and strength of longitudinal associations between cognitive function and depressive symptoms. As detailed in Supplementary Appendix A, Supplementary Section 2, this method is superior to other longitudinal approaches for our analysis. Supplementary Figure S2 illustrates the RI-CLPM structure employed in our study. The random intercepts reflect each participant’s average, stable level of cognitive function, internet use, and depressive symptoms. The RI-CLPM requires longitudinal measurement invariance to validly model change over time. This was assessed by confirmatory factor analysis (CFA), which demonstrated strong measurement invariance for the cognitive function construct. Specifically, invariance testing confirmed the stability of both factor loadings and intercepts across measurement occasions, fulfilling the necessary condition for robust longitudinal analysis [19, 35, 45]. In our study, CFA established scalar longitudinal invariance for both the cognitive function and depression symptom scales across three time points. This invariance, which permitted correlations between residual error variances for the same items and constrained factor loadings and intercepts, validated the appropriateness of the subsequent longitudinal cross-lagged analysis between these constructs. Longitudinal measurement invariance was assessed for cognitive function and depressive symptom scales using nested model comparisons. Scalar invariance was supported for both scales (Details in Supplementary Appendix A, Supplementary Table S2).

To enhance parsimony, a series of four-level nested RI-CLPMs were compared. Model 1 established a baseline model incorporating within-wave correlations and autoregressive paths (six latent variables representing cognitive function and depressive symptoms at three time points; four autoregressive paths; three within-wave correlation parameters). Subsequent models incrementally added parameters: Model 2 tested the equivalence of autoregressive paths across time; Model 3 introduced cross-lagged paths to assess reciprocal effects; and Model 4 evaluated the “stationarity” of cross-lagged paths. All models constrained autoregressive and cross-lagged paths to be invariant across waves and included covariates predicting intercepts. Model comparison, using the corrected scaled Chi-square difference test, determined the optimal model specification based on fit indices. To examine the potential mediating role of internet use in this relationship, Model 5 tested three indirect effects: the influence of prior cognitive function on subsequent depressive symptoms, and prior depressive symptoms on subsequent cognitive function, both mediated by current internet use. These indirect effects were calculated as the product of relevant direct effects. Subsequent models (4i and 5i) replicated the analyses of Models 4 and 5, respectively, but incorporated relevant covariates (including gender and education level as time-invariant factors, and other covariates as time-varying). Equality constraints were applied to autoregressive and cross-lagged paths, and paths from time-invariant predictors to the outcome variables, in accordance with the assumption of temporal stability in RI-CLPM.

Model parameters were estimated using maximum likelihood estimation with robust standard errors (MLR), accounting for non-normality in the data. Model fit was assessed using the Satorra-Bentler scaled chi-square test (S-B χ^2^), root mean square error of approximation (RMSEA), comparative fit index (CFI), and Tucker-Lewis index (TLI). Acceptable model fit was defined as RMSEA <0.08 (with RMSEA <0.05 indicating excellent fit), and CFI/TLI >0.90 (with CFI/TLI >0.95 indicating excellent fit) [35]. Given the sensitivity of the chi-square difference test with large samples, model comparisons primarily relied on changes in the comparative fit index (ΔCFI) and root mean square error of approximation (ΔRMSEA) between nested models. Invariance was considered acceptable when ΔCFI <0.01 and ΔRMSEA <0.015 [46].

Descriptive statistics were computed using Stata 17.0. Longitudinal analyses, employing the robust Full Information Maximum Likelihood (FIML) estimator to accommodate missing data, were performed using Mplus 8.0 [14]. All RI-CLPMs were estimated using FIML. Unstandardized coefficients (β*), standardized coefficients(*β*), standard errors, and p-values are presented. The data, analysis scripts, and Mplus output are available in the Supplementary Material.

## Results

### Descriptive Statistics

Table 1 details sample characteristics. Mean scores for cognitive function across the three assessment waves (2015, 2018, 2020) were 12.25, 13.30, and 13.49, respectively; corresponding means for depressive symptoms (CESD-10) were 7.13, 7.45, and 7.65. Internet use prevalence increased significantly from 8.05% in 2015 to 18.44% in 2018 and 52.71% in 2020. Repeated-measures ANOVA indicated a significant temporal increase in depressive symptoms (F = 69.67, p < 0.001) and a significant decrease in cognitive function (F = 86.61, p < 0.001) over the study period. Significant negative correlations were observed between cognitive function and depressive symptoms at each time point (p < 0.001). Furthermore, cognitive function was negatively correlated with subsequent depressive symptoms (p < 0.001), and depressive symptoms were negatively correlated with subsequent cognitive function (p < 0.001). Conversely, internet use was positively correlated with cognitive function and negatively correlated with depressive symptoms at the same time point (p < 0.001) (See Supplementary Appendix A, Supplementary Table S3).

**TABLE 1:** Sample Characteristics of Cognitive function, Depressive Symptoms, Internet use and Covariates. (China, 2015–2020).

Variables	Baseline 2015 (N = 9,610)		Wave-4 2018 (N = 6,502)		Wave-5 2020 (N = 5,883)	
	N	%	N	%	N	%
Age (Mean, SD)	58.84	8.70	61.05	8.29	60.93	8.21
Sex						
Male	5,298	55.13	3,696	56.84	3,341	56.79
Female	4,312	44.87	2,806	43.16	2,542	43.21
Residence type						
Rural	6,555	68.20	4,352	66.93	3,475	59.10
City/Town	3,055	31.80	2,150	33.07	2,408	40.90
Marital status						
Married	8,699	90.50	5,853	90.02	5,230	88.90
Single, divorced or widowed	911	9.50	649	9.98	653	11.10
Education						
No formal education(illiterate)	1,082	11.20	409	6.29	332	5.64
primary school	4,378	45.60	2,835	43.60	2,513	42.72
Middle school and above	4,150	43.20	3,258	50.11	3,038	51.64
Number of chronic disease	(N = 7,701)		(N = 5,945)		(N = 5,883)	
0	2,898	37.63	1,171	19.70	1,177	20.00
1	2027	26.32	1,304	21.93	1,294	22.00
2 and above	2,776	36.05	3,470	58.37	3,412	58.00
Smoking						
Current smoker	2,979	31.00	1930	29.68	1,677	28.51
Current no smoking	6,631	69.00	4,572	70.32	4,206	71.49
Drinking						
Current drinker	3,924	40.80	2,677	41.17	2,531	43.02
Current no drinking	5,686	59.20	3,825	58.83	3,352	56.98
Intensity of regular physical activities						
Rarely or never	5,155	53.67	383	5.89	390	6.63
Some light or moderate physical activities	2,633	27.41	3,973	61.10	3,379	57.44
Some vigorous physical activities	1817	18.92	2,146	33.01	2,114	35.93
Internet users						
Yes	774	8.05	1,199	18.44	3,101	52.71
No	8,836	91.95	5,303	81.56	2,782	47.29

Scale	Range	Mean	SD	Mean	SD	Mean	SD
Instrument Activities of daily living IADL	0–18	0.53	1.62	0.49	1.54	0.50	1.59
Cognitive function, mMMSE	0–21	12.25	3.18	13.30	3.51	13.49	3.14
Depressive symptoms CESD-10	0–30	7.13	5.90	7.45	6.01	7.65	6.05

### Cognitive Function and Depressive Symptoms

**FIGURE 1:**
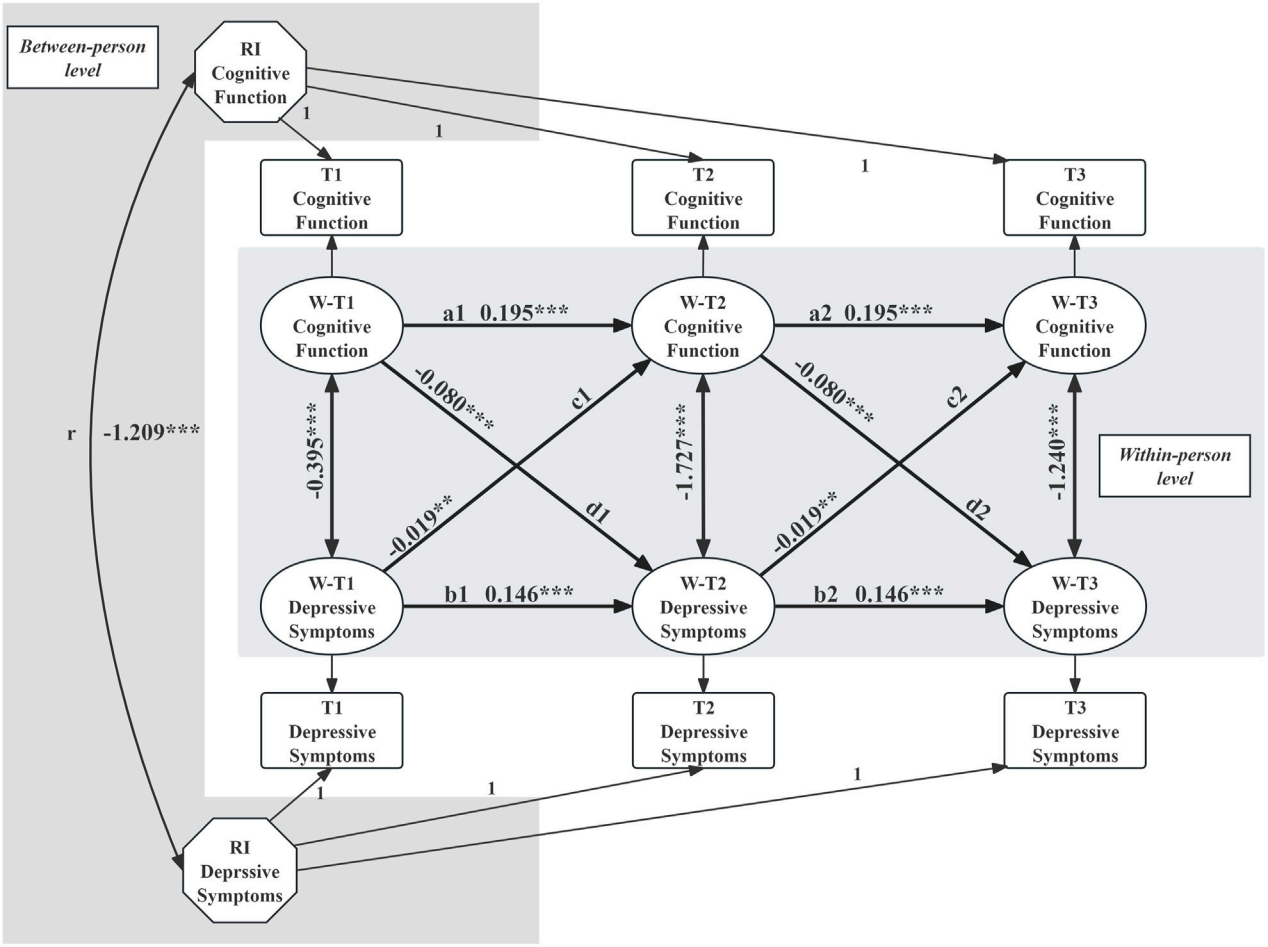
Random-intercept cross-lagged panel model was employed to examine the reciprocal relationship between depressive symptoms and cognitive function. (China, 2015–2020). [Note: The model included autoregressive effects (a and b) and cross-lagged effects (c and d), with unstandardized regression coefficients reported. The correlation between intercept factors is denoted as “r.” Significance levels are indicated as follows: ***p < 0.001, **p < 0.05. (RI = random intercept; T = time point).].

Table 2 displays the fit indices for the series of RI-CLPMs modeling the reciprocal relationship between cognitive function and depressive symptoms. All models exhibited acceptable fit. Constraining path coefficients across waves did not significantly alter model fit. Model 4i, which included covariates and is illustrated in Figure 1, demonstrated excellent fit (RMSEA = 0.017, CFI = 0.971, TLI = 0.955). The two intercept factor (RI_CF_, RI_DS_) were negative correlated (unstandardized path coefficients β* = −1.209, *p* < 0.001) at the between-person level. Within-person analyses revealed significant autoregressive effects: prior cognitive function predicted subsequent cognitive function (a1, a2: β* = 0.195, p < 0.001), and prior depressive symptoms predicted subsequent depressive symptoms (b1, b2: β* = 0.146, p < 0.001). Cross-lagged effects showed that higher levels of prior cognitive function predicted milder subsequent depressive symptoms (d1, d2: β* = −0.080, p < 0.001). In addition, more severe prior depressive symptoms were related to subsequent lower levels of cognitive function (c1,c2: β* = −0.019, p < 0.05). All estimated parameters for Model 4i are shown in Supplementary Appendix A, Supplementary Table S4.

**TABLE 2:** Fit indices of Random Intercept Cross-Lagged panel Models and mediation analysis.(China, 2015–2020).

Model	S-B χ2	df	RMSEA	CFI	TLI	△S-B χ2	△RMSEA	△CFI	*p*
1. Model 1, correlations within time points and autoregressive paths between time points	28.441	5	0.022	0.998	0.995	-	-	-	<0.001
2. Model 2, equating autoregressive paths	81.315	7	0.033	0.995	0.989	52.874	0.011	−0.003	<0.001
3. Model 3, plus cross-lagged paths	53.327	3	0.042	0.997	0.983	−27.988	0.009	0.002	<0.001
4. Model 4, equating cross-lagged paths	67.180	5	0.036	0.996	0.988	13.853	−0.006	−0.001	<0.001
4i. Model 4i, adjustment for covariates based on Model 4	336.874	109	0.017	0.971	0.955	-	-	-	<0.001
5. Model 5, plus two cross-lagged paths that shared Internet use as a mediator	1,014.889	15	0.075	0.953	0.903	-	-	-	<0.001
5i. Model 5i, adjustment for covariates based on Model 5	763.943	171	0.022	0.958	0.934	-	-	-	<0.001

S-B χ2 = Satorra-Bentler Chi-square test statistic; df = degree of freedom; CFI, comparative fit index; TLI, tucker lewis index; RMSEA, root mean square error of approximation; SRMR, standardized root mean square residual. “-” represents these models are not nested, Δ represents the comparisons of the model fit indexes of S-B χ 2, CFI, RMSEA, and df.

### Mediating Effect Results

**FIGURE 2:**
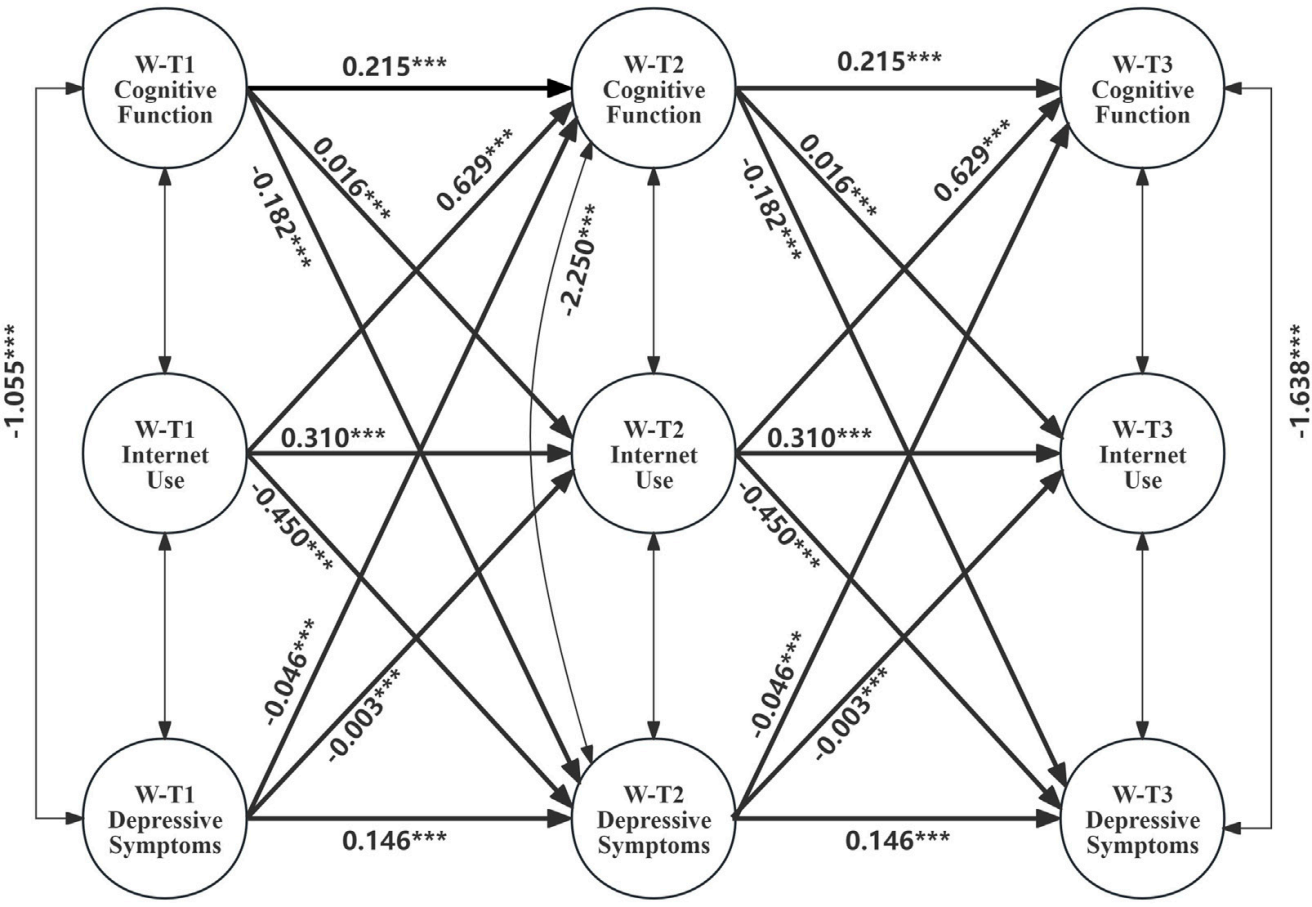
Random-Intercept Cross-Lagged Panel Model for mediating effects of Internet Use on the reciprocal relationship between Cognitive Function and Depressive Symptoms.(China, 2015–2020). [Note: W-T1 represents within-person variable in 2015; W-T2 represents within-person variable in 2018; W-T3 represents within-person variable in 2020. Unstandardized regression coefficients are presented. ***p < 0.001, **p < 0.05, *p < 0.1.].

Model 5i, incorporating three indirect paths through internet use as a mediator and adjusting for control variables, demonstrated good fit to the data (RMSEA = 0.022, CFI = 0.958, TLI = 0.934). Critically, compared to Model 4i, the 3-year cross-lagged effects of depressive symptoms on subsequent cognitive function (β* = −0.046, p < 0.001) and prior cognitive function on subsequent depressive symptoms (β* = −0.182, p < 0.001) were substantially altered. Importantly, as Figure 2 present that the RI-CLPM also revealed significant indirect effects of prior cognitive function on subsequent depressive symptoms, and prior depressive symptoms on subsequent cognitive function, both mediated by internet use. Specifically, Analysis revealed significant positive associations between prior cognitive function and subsequent internet use (β* = 0.016, p < 0.001), and between prior internet use and subsequent reductions in depressive symptoms (β* = −0.450, p < 0.001). Conversely, a significant negative association emerged between prior depressive symptoms and subsequent internet use (β* = −0.003, p < 0.001), with subsequent internet use positively associated with cognitive function (β* = 0.629, p < 0.001). The estimated parameters of Model 5i shows in Table 3.

**TABLE 3:** Estimated coefficients in Model 5i. (China, 2015–2020).

Model pathway	β*	SE	β	SE
Between-person level				
CF(T1) → RI_CF_	1.000	0.000	0.645***	0.011
CF(T2) → RI_CF_	1.000	0.000	0.562***	0.011
CF(T3) → RI_CF_	1.000	0.000	0.600***	0.011
DS(T1) → RI_DS_	1.000	0.000	0.608**	0.012
DS(T2) → RI_DS_	1.000	0.000	0.562***	0.013
DS(T3) → RI_DS_	1.000	0.000	0.573***	0.012
IU(T3) → RI_IU_	1.000	0.000	0.395***	0.028
IU(T3) → RI_IU_	1.000	0.000	0.311***	0.023
IU(T3) → RI_IU_	1.000	0.000	0.225***	0.016
Within-person level				
Autoregressive paths				
CF(T1) → CF(T2)	0.215***	0.022	0.172***	0.018
CF(T2) → CF(T3)	0.215***	0.022	0.239***	0.025
DS(T1) → DS(T2)	0.146***	0.025	0.130***	0.021
DS(T2) → DS(T3)	0.146***	0.025	0.151***	0.026
IU(T1) → IU(T2)	0.310***	0.018	0.236***	0.015
IU(T2) → IU(T3)	0.310***	0.018	0.218***	0.014
Cross-lagged paths				
CF(T1) → DS(T2)	−0.182***	0.025	−0.084***	0.012
CF(T2) → DS(T3)	−0.182***	0.025	−0.109***	0.015
CF(T1) → IU(T2)	0.016***	0.001	0.106***	0.010
CF(T2) → IU(T3)	0.016***	0.001	0.093***	0.009
IU(T1) → CF(T2)	0.629***	0.078	0.056***	0.007
IU(T2) → CF(T3)	0.629***	0.078	0.082***	0.010
DS(T1) → CF(T2)	−0.046***	0.008	−0.071***	0.011
DS(T2) → CF(T3)	−0.046***	0.008	−0.088***	0.015
DS(T1) → IU(T2)	−0.003***	0.001	−0.034***	0.011
DS(T2) → IU(T3)	−0.003***	0.001	−0.027***	0.008
IU(T1) → DS(T2)	−0.450***	0.156	−0.023***	0.008
IU(T2) → DS(T3)	−0.450***	0.156	−0.032***	0.011
(Residual) correlations				
CF(T1) ↔ DS(T1)	−1.055***	0.167	−0.119***	0.018
CF(T1) ↔ IU(T1)	0.068***	0.008	0.127***	0.014
DS(T1) ↔ IU(T1)	−0.033**	0.014	−0.033**	0.014
CF(T2) ↔ DS(T2)	−2.250***	0.237	−0.192***	0.021
CF(T2) ↔ IU(T2)	0.095***	0.011	0.117***	0.014
DS(T2) ↔ IU(T2)	−0.076***	0.018	−0.055***	0.013
CF(T3) ↔ DS(T3)	−1.638***	0.167	−0.169***	0.017
CF(T3) ↔ IU(T3)	0.090***	0.014	0.097***	0.014
DS(T3) ↔ IU(T3)	−0.081***	0.024	−0.048***	0.014

Baseline N = 9,610; bootstrap replications = 500. β = standardized path coefficients; β* = unstandardized path coefficients; SE, standard error; CF, cognitive function; DS, depressive symptoms; IU, Internet use; RI, random intercept; T1/T3 = time one and time 3. ***p < 0.001, **p < 0.05, *p < 0.1.

Table 4 presents the mediation analysis results from Model 5i. Bootstrap estimates with 95% CIs show internet use at T2 (2018) significantly mediated both pathways: (1) between T1 (2015) cognitive function and T3 (2020) depressive symptoms (Indirect effect: standardized path coefficients *β* = −0.003, p = 0.005), accounting for 8.58% of the total effect (indirect/ total = −0.003/-0.035); and (2) between T1 depressive symptoms and T3 cognitive function (Indirect effect:*β* = −0.003, p = 0.003), representing 9.69% of the total effect (−0.003/-0.031). The percentages were calculated using MacKinnon’s proportion mediated method (Proportion Mediated (%) = (Indirect Effect/ Total Effect) × 100). These findings remained robust in sensitivity analyses controlling for COVID-19 related variables (self-isolation duration and pandemic-related fear or anxiety; see Supplementary Appendix A, Supplementary Section 4).

**TABLE 4:** Direct and indirect effects in Model 5i. (China, 2015–2020).

Model pathway	Effect	β	SE	*P* value	95% CI
Overall sample	(Baseline 2015: N = 9,610)				
pathway from CF T1 to DS T3	Total effect	−0.035	0.005	<0.001	−0.045, −0.025
pathway from CF T1 to CF T2 to DS T3	Specific indirect 1	−0.019	0.003	<0.001	−0.025, −0.012
pathway from CF T1 to DS T2 to DS T3	Specific indirect 2	−0.013	0.003	<0.001	−0.018, −0.008
pathway from CF T1 to IU T2 to DS T3	Indirect effect	**−0.003**	**0.001**	**0.005**	**−0.006, -0.001**
pathway from DS T1 to CF T3	Total effect	−0.031	0.006	<0.001	−0.042, −0.020
pathway from DS T1 to CF T2 to CF T3	Specific indirect 1	−0.017	0.004	<0.001	−0.023, −0.011
pathway from DS T1 to DS T2 to CF T3	Specific indirect 2	−0.012	0.002	<0.001	−0.017, −0.006
pathway from DS T1 to IU T2 to CF T3	Indirect effect	**−0.003**	**0.001**	**0.003**	**−0.005, -0.001**
Age subgroups					
Age: 45–64	(Baseline 2015: N = 7,073)				
pathway from CF T1 to DS T3	Total effect	−0.048	0.006	<0.001	−0.059, −0.037
pathway from CF T1 to IU T2 to DS T3	Indirect effect	**−0.003**	**0.001**	**0.018**	**−0.006, -0.001**
pathway from DS T1 to CF T3	Total effect	−0.053	0.006	<0.001	−0.057, −0.032
pathway from DS T1 to IU T2 to CF T3	Indirect effect	**−0.002**	**0.001**	**0.009**	**−0.004, -0.001**
Age: 65 and over	(Baseline 2015: N = 2,537)				
pathway from CF T1 to DS T3	Total effect	−0.057	0.012	<0.001	−0.080, −0.033
pathway from CF T1 to IU T2 to DS T3	Indirect effect	**−0.003**	**0.001**	**0.020**	**−0.006, -0.001**
pathway from DS T1 to CF T3	Total effect	−0.028	0.012	0.023	−0.051, −0.004
pathway from DS T1 to IU T2 to CF T3	Indirect effect	−0.001	0.001	0.177	−0.003, 0.001

All effects are standardized, CF, Cognitive function; DS, depressive symptoms; IU, internet use; β, effect: standardized path coefficients, SE, standard error. T1-T3, Time one and 3, Statistically significant mediation effects (p < 0.05) are indicated in bold, Proportion Mediated (%) = (Indirect Effect/ Total Effect)×100.

Considering the controversy surrounding current findings regrading the impact of internet use on cognitive function or depressive symptoms among different age populations [21, 28], we conducted age-stratified analyses (45–64 vs ≥ 65 years) using Model 5i. Table 4 also reveals that internet use mediated the cognition-depression pathway in two age subgroups; particularly, the magnitude of this mediation was slightly greater in middle-aged adults (age45-64: 6.30%, −0.003/-0.048, p = 0.018) than in older adults (age≥65: 5.30%, −0.003/-0.057; p = 0.020). Complete model parameters and RI-CLPM path coefficient comparisons are available in Supplementary Appendix A, Supplementary Tables S5, S6.

## Discussion

This study provides novel evidence from the first large-scale, nationally representative longitudinal investigation examining the dynamic interrelationships among cognitive function, depressive symptoms, and internet use in Chinese adults aged 45 and older over a 5-year period. Our RI-CLPM analyses demonstrated a robust bidirectional association between depression and cognition (all p < 0.05), supporting a potentially cyclical relationship between these domains. Importantly, we identified internet use as a significant mediator in this association. Age-stratified analyses revealed that the mediation effects were generally stronger in middle-aged adults (45–64 years) than in older adults (≥65 years), although this difference was statistically significant only for the cognition-depression link. These findings advance our understanding of the complex psychocognitive mechanisms in middle-aged and older adults, while highlighting the need for further research to elucidate the observed age-dependent variations in mediation effects.

Firstly, to comprehensively assess cognitive function, we employed a three-dimensional scale encompassing orientation/calculation, episodic memory, and visual construction. Our analysis of a nationally representative longitudinal dataset revealed a bidirectional causal relationship between cognitive function and depressive symptoms, consistent with prior research conducted in the USA and Europe [34, 41]. Notably, our results demonstrated a stronger negative association between prior cognitive function and subsequent depressive symptoms than *vice versa*. After accounting for between-person differences, our model revealed a connection between changes in depression and cognition at the within-person level. This association might stem from severe depression impairing self-regulation, which could lead to unhealthy behaviors that increase the risk of cognitive decline [41]. Furthermore, evidence from cognitive task studies, biological mechanism investigations, and randomized controlled trials supports the link between depressive symptoms and cognitive impairment [7, 18, 47]. While acknowledging this potential bidirectional relationship, our study further examined how other factors influence their trajectories over time. Importantly, the significant reciprocal relationship between cognition and depression persisted even after controlling for covariates.

Secondly, prior research has indicated that the reciprocal relationship between cognition and depression likely involves mediating pathways encompassing multiple mechanisms, including social isolation [48], social participation [49], physical activity [50], and sleep quality [19]. Our findings provide valuable insights into the mechanisms driving the reciprocal relationship between cognition and depression. While internet use showed statistically significant but modest mediation effects (indirect effect: *β* = −0.003), these findings still hold importance. The effect sizes are comparable to other known modifiable risk factors in aging research. Importantly, internet users demonstrated better subsequent cognitive function and fewer depressive symptoms, suggesting meaningful clinical implications. These results align with the cognitive reserve theory, adults with better baseline cognition may benefit more from internet use through greater digital skill acquisition and selective engagement in stimulating online activities [21]. The relatively small effects may reflect our binary internet use measure, which likely underestimates the benefits of more intensive or high-quality digital engagement. Specifically, internet use offers access to social and emotional support, which may reduce social isolation and enhance overall wellbeing, thereby alleviating depressive symptoms [23]. Moreover, individuals with lower baseline cognitive function may be less likely to adopt internet use or engage in online social interactions, limiting their access to potential benefits and potentially exacerbating depressive symptoms. Conversely, we also observed internet use mediating the pathway between depression and cognitive function. This might occur because individuals experiencing more severe depression tend to withdraw socially, resulting in reduced internet use for social engagement [28]. Consequently, they may miss out on the cognitive benefits associated with internet use, including cognitive stimulation, enhanced social interaction, and access to health-related information, when compared to their more digitally engaged peers [22]. By contrast, individuals with milder depressive symptoms may be more likely to leverage digital tools to increase their social engagement, which may provide beneficial cognitive stimulation and potentially offer protection against subsequent cognitive decline [21, 51].

Furthermore, similar to established treatments for depression and cognitive impairment (e.g., pharmacotherapy, psychotherapy, neurostimulation, and exercise interventions), research on the beneficial effects of internet use faces several limitations. These include substantial heterogeneity in digital intervention quality and insufficient evidence from diverse socioeconomic populations [44]. For instance, several studies have raised concerns that inappropriate internet use may worsen depressive symptoms. Therefore, while promoting internet use among middle-aged and older adults, health professionals should guide them toward responsible usage to prevent over-dependence and potential negative health consequences [28]. Additionally, internet experience varies significantly across populations due to factors such as age, gender, education, geographic location, and socioeconomic status [23]. For example, For example, compared to younger adults, older adults may derive fewer benefits in terms of social network expansion and interpersonal relationship improvement through internet use, which are crucial for mental health resilience [24]. In light of these findings, recent studies suggest that implementing timely interventions when early changes are observed can help mitigate dementia-related cognitive decline and improve mental health outcomes [12].

Although the observed mediation effects of internet use were modest in magnitude, this likely reflects the complex, multifactorial etiology linking cognitive decline and depression in older adults. At the biological level, shared neuropathological processes (e.g., vascular changes and neuroinflammation) may drive both cognitive impairment and depressive symptoms regardless of internet use [11]. Socially, factors like loneliness, reduced social networks, and socioeconomic disadvantages that are often less modifiable through digital means may exert stronger direct effects on mental health [22]. Behaviorally, physical inactivity and sensory impairments could mediate the relationship while remaining relatively unaffected by internet engagement [50]. These competing pathways help contextualize why digital interventions alone capture only a portion of the observed association. Nevertheless, the population-level impact of scalable digital interventions may offset their individually modest effects, particularly given the high prevalence of subthreshold cognitive and depressive symptoms in aging populations, the progressive nature of cognitive decline where early small benefits may yield disproportionate long-term advantages [34], and the unique accessibility benefits for underserved groups (e.g., rural or mobility-impaired elders) who face systemic barriers to traditional care. Future interventions should adopt multimodal approaches that combine internet-based tools with established protective factors (e.g., physical activity programs and hearing correction), develop personalization algorithms targeting users’ specific risk profiles (e.g., those with vascular risks versus social isolation), and prioritize implementation research to optimize real-world effectiveness beyond efficacy studies [52].

Finally, our age-stratified analyses demonstrated that while middle-aged adults (45–64 years) generally exhibited stronger mediation effects of internet use in the cognition-depression pathway compared to older adults (≥65 years), with most comparisons reaching statistical significance (p < 0.05), some pathways showed marginally significant differences (0.05 < p < 0.1) that warrant cautious interpretation. These findings preliminarily suggest middle-aged adults may derive greater cognitive-mental health benefits from internet use, potentially making them more responsive to technology-based interventions. These exploratory findings align with existing research on age-related digital engagement [23] and underscore the importance of developing digital literacy [32]. The observed age-group differences highlight the need for age-appropriate digital support strategies, particularly for older adults who may benefit from tailored approaches to optimize internet use benefits. Further research should prioritize replication in larger samples to confirm these age-dependent effects, investigate platform-specific benefits, and examine how digital access disparities may moderate the mediation effects, in order to inform more equitable digital health policies that account for age-related variations in technology’s psychological impacts [32, 51].

This study demonstrates several important strengths. First, this nationally representative, longitudinal study confirms a potential vicious cycle between cognition and depression and identifies internet use as a mediating factor. These findings fill a gap in the literature, enriching the theoretical understanding of this link. By exploring internet use in healthy aging, our study demonstrates the potential of digital tools to alleviate cognitive decline and depressive symptoms. Second, by employing the RI-CLPM, we differentiate within-person from between-person associations, strengthening the reliability of our results. Third, our subgroup analyses identify distinct mediation effects of internet use between middle-aged (45–64 years) and older adults (≥65 years), providing empirical support for developing age-specific interventions. Lastly, our findings contribute to advancing healthy aging practices in middle-income countries, particularly through technology interventions aimed at improving the mental health and cognitive abilities of middle-aged and older adults.

### Limitations

Several limitations warrant consideration when interpreting our findings. First, potential bias may arise from non-random participant attrition, potentially leading to effect underestimation. To mitigate this, we conducted attrition bias checks (Supplementary Appendix SA, Supplementary Table S7) and used full information maximum likelihood estimation. Additionally, unmeasured time-varying confounders (e.g., income fluctuations, family support changes, undiagnosed neuropathology) could affect the observed relationships. Second, our binary measure of internet use might mask meaningful variation in usage patterns that differentially affect cognition-depression links. This limitation prevents analysis of specific online activities or usage intensity, possibly obscuring non-linear relationships (e.g., U-shaped effects where moderate use is most beneficial). Given the rapid evolution of digital technologies and their varied applications, accelerated by the COVID-19 pandemic, this simplified measure may not fully capture the complexity of internet engagement’s relationship with cognitive and mental health outcomes. While acknowledging the inherent constraints of our longitudinal design, it’s important to consider that our reported effect sizes may represent a conservative estimate of the true temporal relationships. Furthermore, the observed mediation effects may be influenced by the chosen measurement intervals and the level of detail in assessing digital behavior. Therefore, future studies should incorporate validated multidimensional measures of internet use, capturing duration, purpose, and platform specificity. They should employ higher-frequency assessments to better capture dynamic processes. Measurement burst designs should be utilized to separate transient from sustained effects. Activity-specific analytical frameworks should be applied to identify differential impacts of various online behaviors. Finally, proactive retention strategies should be implemented (e.g., mixed-mode follow-ups, incentives, caregiver tracking for cognitively vulnerable subgroups) to minimize attrition bias.

### Conclusions

In summary, we analyzed 5 years of longitudinal data from the CHARLS survey, focusing on Chinese middle-aged and older adults, to examine the bidirectional causal relationship between cognitive function and depressive symptoms and the mediating role of internet use. We also compared the strength of this mediating effect between middle-aged and older adults. These findings are especially significant for middle-aged and older adults, who may need to reduce face-to-face social interactions because of physical health issues or age-related limitations, and thus rely on alternative approaches to maintain cognitive and mental health. Given the aging global population, elucidating the pathways between depression and cognitive decline is crucial to interrupting the potential vicious cycle. Multidisciplinary interventions targeting both the reduction of depression and the delay of cognitive deterioration in these populations should be prioritized.

## Data Availability

The raw data used in this study are freely available from the China Health and Retirement Longitudinal Study (CHARLS; http://charls.pku.edu.cn/en), a nationally representative longitudinal survey of population in China organized by Peking University National School of Development. The analytical methods and statistic codes from this study will be made available to other researchers on request.

## Abbreviations

CHARLS, China Health and Retirement Longitudinal Study; mMMSE, Modified Mini-Mental State Examination; CESD-10, Center for Epidemiologic Studies Depression Scale; RI-CLPM, Random Intercept Cross-Lagged Panel Model; IADL, Instrument Activities of daily living.
