# Supplementary material for: Mediating Role of Internet Use in Cognitive-Depressive Pathways: A Random Intercept Cross-Lagged Panel Modeling Approach
Source: Int J Public Health. 2025 Oct 21;70:1608478. doi: 10.3389/ijph.2025.1608478 (PMC12583110; doi:10.3389/ijph.2025.1608478)
Supplement: Supplementary file 1 [file Supplementaryfile2.doc]

Model 4i

Cognition- depression

THE MODEL ESTIMATION TERMINATED NORMALLY

MODEL FIT INFORMATION

Number of Free Parameters 74

Loglikelihood

H0 Value -102442.268

H1 Value -102273.831

Information Criteria

Akaike (AIC) 205032.536

Bayesian (BIC) 205538.824

Sample-Size Adjusted BIC 205303.670

(n* = (n + 2) / 24)

Chi-Square Test of Model Fit

Value 336.874

Degrees of Freedom 109

P-Value 0.0000

RMSEA (Root Mean Square Error Of Approximation)

Estimate 0.017

90 Percent C.I. 0.015 0.020

Probability RMSEA <= .05 1.000

CFI/TLI

CFI 0.971

TLI 0.955

Chi-Square Test of Model Fit for the Baseline Model

Value 8110.084

Degrees of Freedom 171

P-Value 0.0000

SRMR (Standardized Root Mean Square Residual)

Value 0.026

MODEL RESULTS

Two-Tailed

Estimate S.E. Est./S.E. P-Value

RIX BY

X1 1.000 0.000 999.000 999.000

X2 1.000 0.000 999.000 999.000

X3 1.000 0.000 999.000 999.000

RIY BY

Y1 1.000 0.000 999.000 999.000

Y2 1.000 0.000 999.000 999.000

Y3 1.000 0.000 999.000 999.000

WX1 BY

X1 1.000 0.000 999.000 999.000

WX2 BY

X2 1.000 0.000 999.000 999.000

WX3 BY

X3 1.000 0.000 999.000 999.000

WY1 BY

Y1 1.000 0.000 999.000 999.000

WY2 BY

Y2 1.000 0.000 999.000 999.000

WY3 BY

Y3 1.000 0.000 999.000 999.000

WX2 ON

WX1 0.195 0.022 8.914 0.000

WY1 -0.019 0.009 -2.052 0.040

WY2 ON

WY1 0.146 0.024 6.133 0.000

WX1 -0.080 0.029 -2.756 0.006

WX3 ON

WX2 0.195 0.022 8.914 0.000

WY2 -0.019 0.009 -2.052 0.040

WY3 ON

WY2 0.146 0.024 6.133 0.000

WX2 -0.080 0.029 -2.756 0.006

RIX ON

SEX -0.016 0.072 -0.221 0.825

EDU 1.831 0.049 37.091 0.000

RIY ON

SEX -1.261 0.132 -9.564 0.000

EDU -0.947 0.085 -11.084 0.000

WX2 ON

AG2 -0.051 0.005 -9.264 0.000

CX2 0.717 0.086 8.350 0.000

MAR2 0.511 0.147 3.465 0.001

SMOK2 -0.474 0.095 -4.961 0.000

DK2 0.168 0.084 2.013 0.044

TPA2 -0.010 0.073 -0.138 0.891

CM2 0.000 0.024 0.018 0.985

IAM2 -0.174 0.032 -5.383 0.000

WY2 ON

AG2 -0.072 0.009 -8.273 0.000

CX2 -0.881 0.130 -6.785 0.000

MAR2 -1.044 0.241 -4.339 0.000

SMOK2 0.302 0.143 2.111 0.035

DK2 -0.438 0.130 -3.378 0.001

TPA2 0.244 0.113 2.159 0.031

CM2 0.468 0.040 11.698 0.000

IAM2 0.624 0.049 12.771 0.000

WX3 ON

AG3 -0.038 0.005 -7.409 0.000

CX3 0.547 0.070 7.848 0.000

MAR3 0.365 0.114 3.198 0.001

SMOK3 -0.045 0.079 -0.567 0.571

DK3 0.254 0.068 3.751 0.000

IAM3 -0.168 0.024 -6.991 0.000

TPA3 0.103 0.062 1.660 0.097

CM3 0.009 0.018 0.519 0.604

WY3 ON

AG3 -0.013 0.008 -1.553 0.121

CX3 -0.882 0.127 -6.955 0.000

MAR3 -0.838 0.228 -3.670 0.000

SMOK3 -0.129 0.152 -0.848 0.397

DK3 -0.517 0.127 -4.065 0.000

IAM3 0.577 0.045 12.874 0.000

TPA3 0.344 0.102 3.371 0.001

CM3 0.399 0.036 11.222 0.000

WX1 ON

AG1 -0.043 0.004 -10.071 0.000

CX1 0.673 0.071 9.432 0.000

MAR1 0.432 0.126 3.427 0.001

SMOK1 -0.149 0.076 -1.951 0.051

DK1 0.165 0.068 2.405 0.016

TPA1 -0.101 0.039 -2.609 0.009

IAM1 -0.165 0.026 -6.365 0.000

CM1 0.036 0.024 1.536 0.125

WY1 ON

AG1 -0.043 0.008 -5.356 0.000

CX1 -0.868 0.129 -6.705 0.000

MAR1 -1.590 0.264 -6.033 0.000

SMOK1 0.423 0.139 3.038 0.002

DK1 -0.080 0.130 -0.615 0.538

TPA1 0.087 0.075 1.172 0.241

IAM1 0.688 0.051 13.375 0.000

CM1 0.676 0.046 14.766 0.000

RIX WITH

RIY -1.209 0.166 -7.274 0.000

WX1 0.000 0.000 999.000 999.000

WY1 0.000 0.000 999.000 999.000

WX1 WITH

WY1 -0.395 0.166 -2.378 0.017

RIY 0.000 0.000 999.000 999.000

WX2 WITH

WY2 -1.727 0.260 -6.634 0.000

WX3 WITH

WY3 -1.240 0.168 -7.397 0.000

RIY WITH

WY1 0.000 0.000 999.000 999.000

Intercepts

X1 9.406 0.344 27.327 0.000

X2 10.661 0.458 23.264 0.000

X3 10.065 0.430 23.424 0.000

Y1 13.286 0.649 20.463 0.000

Y2 15.807 0.731 21.631 0.000

Y3 12.648 0.723 17.496 0.000

Residual Variances

X1 0.000 0.000 999.000 999.000

X2 0.000 0.000 999.000 999.000

X3 0.000 0.000 999.000 999.000

Y1 0.000 0.000 999.000 999.000

Y2 0.000 0.000 999.000 999.000

Y3 0.000 0.000 999.000 999.000

RIX 3.099 0.128 24.186 0.000

RIY 11.142 0.475 23.471 0.000

WX1 4.401 0.118 37.335 0.000

WX2 6.838 0.190 36.014 0.000

WX3 5.179 0.118 43.841 0.000

WY1 15.973 0.507 31.495 0.000

WY2 20.000 0.627 31.873 0.000

WY3 17.438 0.468 37.233 0.000

STANDARDIZED MODEL RESULTS

STDYX Standardization

Two-Tailed

Estimate S.E. Est./S.E. P-Value

RIX BY

X1 0.680 0.009 72.191 0.000

X2 0.590 0.010 57.785 0.000

X3 0.632 0.010 65.214 0.000

RIY BY

Y1 0.623 0.011 55.539 0.000

Y2 0.574 0.013 45.822 0.000

Y3 0.588 0.012 49.685 0.000

WX1 BY

X1 0.698 0.009 79.161 0.000

WX2 BY

X2 0.774 0.008 100.411 0.000

WX3 BY

X3 0.737 0.008 92.043 0.000

WY1 BY

Y1 0.773 0.009 85.437 0.000

WY2 BY

Y2 0.808 0.009 90.854 0.000

WY3 BY

Y3 0.793 0.009 89.938 0.000

WX2 ON

WX1 0.153 0.017 9.250 0.000

WY1 -0.029 0.014 -2.037 0.042

WY2 ON

WY1 0.129 0.020 6.328 0.000

WX1 -0.036 0.013 -2.743 0.006

WX3 ON

WX2 0.219 0.025 8.634 0.000

WY2 -0.036 0.018 -2.048 0.041

WY3 ON

WY2 0.153 0.025 6.001 0.000

WX2 -0.048 0.017 -2.746 0.006

RIX ON

SEX -0.004 0.017 -0.221 0.825

EDU 0.562 0.014 41.034 0.000

RIY ON

SEX -0.180 0.019 -9.553 0.000

EDU -0.178 0.016 -11.236 0.000

WX2 ON

AG2 -0.154 0.017 -9.246 0.000

CX2 0.117 0.014 8.392 0.000

MAR2 0.057 0.016 3.465 0.001

SMOK2 -0.078 0.016 -4.961 0.000

DK2 0.029 0.015 2.013 0.044

TPA2 -0.002 0.015 -0.138 0.891

CM2 0.000 0.016 0.018 0.985

IAM2 -0.121 0.022 -5.402 0.000

WY2 ON

AG2 -0.125 0.015 -8.303 0.000

CX2 -0.082 0.012 -6.716 0.000

MAR2 -0.066 0.015 -4.322 0.000

SMOK2 0.028 0.013 2.107 0.035

DK2 -0.044 0.013 -3.402 0.001

TPA2 0.029 0.013 2.169 0.030

CM2 0.177 0.016 11.314 0.000

IAM2 0.248 0.019 12.959 0.000

WX3 ON

AG3 -0.129 0.017 -7.437 0.000

CX3 0.106 0.013 7.918 0.000

MAR3 0.049 0.015 3.199 0.001

SMOK3 -0.008 0.014 -0.567 0.570

DK3 0.050 0.013 3.744 0.000

IAM3 -0.150 0.022 -6.905 0.000

TPA3 0.025 0.015 1.666 0.096

CM3 0.008 0.015 0.519 0.604

WY3 ON

AG3 -0.024 0.015 -1.550 0.121

CX3 -0.091 0.013 -6.935 0.000

MAR3 -0.059 0.016 -3.681 0.000

SMOK3 -0.012 0.015 -0.847 0.397

DK3 -0.054 0.013 -4.057 0.000

IAM3 0.274 0.021 13.120 0.000

TPA3 0.045 0.013 3.369 0.001

CM3 0.174 0.016 11.017 0.000

WX1 ON

AG1 -0.167 0.016 -10.147 0.000

CX1 0.143 0.015 9.636 0.000

MAR1 0.055 0.016 3.439 0.001

SMOK1 -0.032 0.016 -1.952 0.051

DK1 0.037 0.016 2.397 0.017

TPA1 -0.036 0.014 -2.614 0.009

IAM1 -0.115 0.018 -6.375 0.000

CM1 0.024 0.016 1.533 0.125

WY1 ON

AG1 -0.085 0.016 -5.334 0.000

CX1 -0.093 0.014 -6.801 0.000

MAR1 -0.102 0.017 -6.038 0.000

SMOK1 0.046 0.015 3.048 0.002

DK1 -0.009 0.015 -0.615 0.539

TPA1 0.016 0.013 1.174 0.240

IAM1 0.243 0.017 14.436 0.000

CM1 0.224 0.015 15.008 0.000

RIX WITH

RIY -0.206 0.027 -7.517 0.000

WX1 0.000 0.000 0.000 1.000

WY1 0.000 0.000 0.000 1.000

WX1 WITH

WY1 -0.047 0.020 -2.407 0.016

RIY 0.000 0.000 0.000 1.000

WX2 WITH

WY2 -0.148 0.022 -6.674 0.000

WX3 WITH

WY3 -0.130 0.017 -7.515 0.000

RIY WITH

WY1 0.000 0.000 0.000 1.000

Intercepts

X1 3.004 0.114 26.280 0.000

X2 2.956 0.130 22.789 0.000

X3 2.991 0.131 22.836 0.000

Y1 2.383 0.115 20.693 0.000

Y2 2.613 0.118 22.183 0.000

Y3 2.142 0.121 17.700 0.000

Residual Variances

X1 0.000 0.000 0.000 1.000

X2 0.000 0.000 0.000 1.000

X3 0.000 0.000 0.000 1.000

Y1 0.000 0.000 0.000 1.000

Y2 0.000 0.000 0.000 1.000

Y3 0.000 0.000 0.000 1.000

RIX 0.685 0.015 46.127 0.000

RIY 0.923 0.009 98.131 0.000

WX1 0.921 0.009 103.496 0.000

WX2 0.878 0.012 73.987 0.000

WX3 0.842 0.015 56.707 0.000

WY1 0.861 0.011 78.069 0.000

WY2 0.837 0.013 63.082 0.000

WY3 0.795 0.015 52.482 0.000

R-SQUARE

Observed Two-Tailed

Variable Estimate S.E. Est./S.E. P-Value

X1 1.000 0.000 999.000 999.000

X2 1.000 0.000 999.000 999.000

X3 1.000 0.000 999.000 999.000

Y1 1.000 0.000 999.000 999.000

Y2 1.000 0.000 999.000 999.000

Y3 1.000 0.000 999.000 999.000

Latent Two-Tailed

Variable Estimate S.E. Est./S.E. P-Value

RIX 0.315 0.015 21.245 0.000

RIY 0.077 0.009 8.182 0.000

WX1 0.079 0.009 8.832 0.000

WX2 0.122 0.012 10.290 0.000

WX3 0.158 0.015 10.675 0.000

WY1 0.139 0.011 12.641 0.000

WY2 0.163 0.013 12.283 0.000

WY3 0.205 0.015 13.501 0.000

CONFIDENCE INTERVALS OF MODEL RESULTS

Lower .5% Lower 2.5% Lower 5% Estimate Upper 5% Upper 2.5% Upper .5%

RIX BY

X1 1.000 1.000 1.000 1.000 1.000 1.000 1.000

X2 1.000 1.000 1.000 1.000 1.000 1.000 1.000

X3 1.000 1.000 1.000 1.000 1.000 1.000 1.000

RIY BY

Y1 1.000 1.000 1.000 1.000 1.000 1.000 1.000

Y2 1.000 1.000 1.000 1.000 1.000 1.000 1.000

Y3 1.000 1.000 1.000 1.000 1.000 1.000 1.000

WX1 BY

X1 1.000 1.000 1.000 1.000 1.000 1.000 1.000

WX2 BY

X2 1.000 1.000 1.000 1.000 1.000 1.000 1.000

WX3 BY

X3 1.000 1.000 1.000 1.000 1.000 1.000 1.000

WY1 BY

Y1 1.000 1.000 1.000 1.000 1.000 1.000 1.000

WY2 BY

Y2 1.000 1.000 1.000 1.000 1.000 1.000 1.000

WY3 BY

Y3 1.000 1.000 1.000 1.000 1.000 1.000 1.000

WX2 ON

WX1 0.139 0.152 0.159 0.195 0.231 0.238 0.251

WY1 -0.042 -0.036 -0.033 -0.019 -0.004 -0.001 0.005

WY2 ON

WY1 0.085 0.100 0.107 0.146 0.186 0.193 0.208

WX1 -0.154 -0.137 -0.128 -0.080 -0.032 -0.023 -0.005

WX3 ON

WX2 0.139 0.152 0.159 0.195 0.231 0.238 0.251

WY2 -0.042 -0.036 -0.033 -0.019 -0.004 -0.001 0.005

WY3 ON

WY2 0.085 0.100 0.107 0.146 0.186 0.193 0.208

WX2 -0.154 -0.137 -0.128 -0.080 -0.032 -0.023 -0.005

RIX ON

SEX -0.201 -0.157 -0.134 -0.016 0.102 0.125 0.169

EDU 1.704 1.734 1.750 1.831 1.912 1.928 1.958

RIY ON

SEX -1.601 -1.520 -1.478 -1.261 -1.044 -1.003 -0.921

EDU -1.166 -1.114 -1.087 -0.947 -0.806 -0.779 -0.727

WX2 ON

AG2 -0.065 -0.061 -0.060 -0.051 -0.042 -0.040 -0.037

CX2 0.496 0.549 0.576 0.717 0.858 0.885 0.938

MAR2 0.131 0.222 0.268 0.511 0.753 0.800 0.890

SMOK2 -0.720 -0.661 -0.631 -0.474 -0.317 -0.287 -0.228

DK2 -0.047 0.004 0.031 0.168 0.306 0.332 0.384

TPA2 -0.198 -0.153 -0.130 -0.010 0.110 0.133 0.178

CM2 -0.060 -0.046 -0.038 0.000 0.039 0.047 0.061

IAM2 -0.257 -0.237 -0.227 -0.174 -0.121 -0.110 -0.091

WY2 ON

AG2 -0.095 -0.089 -0.086 -0.072 -0.058 -0.055 -0.050

CX2 -1.216 -1.136 -1.095 -0.881 -0.668 -0.627 -0.547

MAR2 -1.664 -1.516 -1.440 -1.044 -0.648 -0.572 -0.424

SMOK2 -0.066 0.022 0.067 0.302 0.537 0.582 0.670

DK2 -0.771 -0.691 -0.651 -0.438 -0.224 -0.184 -0.104

TPA2 -0.047 0.022 0.058 0.244 0.429 0.465 0.534

CM2 0.365 0.390 0.402 0.468 0.534 0.546 0.571

IAM2 0.498 0.528 0.544 0.624 0.705 0.720 0.750

WX3 ON

AG3 -0.051 -0.048 -0.046 -0.038 -0.029 -0.028 -0.025

CX3 0.367 0.410 0.432 0.547 0.661 0.683 0.726

MAR3 0.071 0.141 0.177 0.365 0.553 0.589 0.660

SMOK3 -0.248 -0.200 -0.175 -0.045 0.085 0.110 0.159

DK3 0.080 0.121 0.143 0.254 0.366 0.387 0.429

IAM3 -0.229 -0.215 -0.207 -0.168 -0.128 -0.121 -0.106

TPA3 -0.057 -0.019 0.001 0.103 0.205 0.225 0.263

CM3 -0.037 -0.026 -0.020 0.009 0.039 0.045 0.056

WY3 ON

AG3 -0.035 -0.030 -0.027 -0.013 0.001 0.003 0.009

CX3 -1.208 -1.130 -1.090 -0.882 -0.673 -0.633 -0.555

MAR3 -1.426 -1.285 -1.213 -0.838 -0.462 -0.390 -0.250

SMOK3 -0.520 -0.426 -0.379 -0.129 0.121 0.169 0.262

DK3 -0.845 -0.767 -0.727 -0.517 -0.308 -0.268 -0.190

IAM3 0.462 0.489 0.503 0.577 0.651 0.665 0.693

TPA3 0.081 0.144 0.176 0.344 0.512 0.544 0.607

CM3 0.307 0.329 0.340 0.399 0.457 0.468 0.490

WX1 ON

AG1 -0.054 -0.051 -0.050 -0.043 -0.036 -0.035 -0.032

CX1 0.489 0.533 0.555 0.673 0.790 0.813 0.857

MAR1 0.107 0.185 0.225 0.432 0.640 0.680 0.757

SMOK1 -0.345 -0.298 -0.274 -0.149 -0.023 0.001 0.048

DK1 -0.012 0.030 0.052 0.165 0.277 0.299 0.341

TPA1 -0.201 -0.178 -0.165 -0.101 -0.037 -0.025 -0.001

IAM1 -0.232 -0.216 -0.208 -0.165 -0.123 -0.115 -0.099

CM1 -0.025 -0.010 -0.003 0.036 0.075 0.083 0.097

WY1 ON

AG1 -0.064 -0.059 -0.056 -0.043 -0.030 -0.027 -0.022

CX1 -1.201 -1.121 -1.081 -0.868 -0.655 -0.614 -0.534

MAR1 -2.269 -2.107 -2.024 -1.590 -1.157 -1.074 -0.911

SMOK1 0.064 0.150 0.194 0.423 0.652 0.696 0.782

DK1 -0.414 -0.334 -0.294 -0.080 0.134 0.175 0.255

TPA1 -0.105 -0.059 -0.035 0.087 0.210 0.234 0.280

IAM1 0.555 0.587 0.603 0.688 0.773 0.789 0.820

CM1 0.558 0.586 0.600 0.676 0.751 0.765 0.793

RIX WITH

RIY -1.637 -1.535 -1.482 -1.209 -0.936 -0.883 -0.781

WX1 0.000 0.000 0.000 0.000 0.000 0.000 0.000

WY1 0.000 0.000 0.000 0.000 0.000 0.000 0.000

WX1 WITH

WY1 -0.822 -0.720 -0.668 -0.395 -0.122 -0.069 0.033

RIY 0.000 0.000 0.000 0.000 0.000 0.000 0.000

WX2 WITH

WY2 -2.397 -2.237 -2.155 -1.727 -1.298 -1.216 -1.056

WX3 WITH

WY3 -1.671 -1.568 -1.515 -1.240 -0.964 -0.911 -0.808

RIY WITH

WY1 0.000 0.000 0.000 0.000 0.000 0.000 0.000

Intercepts

X1 8.519 8.731 8.839 9.406 9.972 10.080 10.292

X2 9.481 9.763 9.907 10.661 11.415 11.559 11.841

X3 8.958 9.223 9.358 10.065 10.772 10.907 11.172

Y1 11.614 12.014 12.218 13.286 14.354 14.559 14.959

Y2 13.924 14.374 14.604 15.807 17.009 17.239 17.689

Y3 10.786 11.231 11.459 12.648 13.837 14.065 14.510

Residual Variances

X1 0.000 0.000 0.000 0.000 0.000 0.000 0.000

X2 0.000 0.000 0.000 0.000 0.000 0.000 0.000

X3 0.000 0.000 0.000 0.000 0.000 0.000 0.000

Y1 0.000 0.000 0.000 0.000 0.000 0.000 0.000

Y2 0.000 0.000 0.000 0.000 0.000 0.000 0.000

Y3 0.000 0.000 0.000 0.000 0.000 0.000 0.000

RIX 2.769 2.848 2.888 3.099 3.310 3.350 3.429

RIY 9.919 10.212 10.361 11.142 11.923 12.072 12.365

WX1 4.097 4.170 4.207 4.401 4.595 4.632 4.705

WX2 6.349 6.466 6.526 6.838 7.151 7.211 7.328

WX3 4.875 4.948 4.985 5.179 5.374 5.411 5.484

WY1 14.667 14.979 15.139 15.973 16.807 16.967 17.279

WY2 18.383 18.770 18.967 20.000 21.032 21.229 21.616

WY3 16.232 16.520 16.668 17.438 18.209 18.356 18.645

CONFIDENCE INTERVALS OF STANDARDIZED MODEL RESULTS

STDYX Standardization

Lower .5% Lower 2.5% Lower 5% Estimate Upper 5% Upper 2.5% Upper .5%

RIX BY

X1 0.655 0.661 0.664 0.680 0.695 0.698 0.704

X2 0.564 0.570 0.573 0.590 0.607 0.610 0.616

X3 0.607 0.613 0.616 0.632 0.648 0.651 0.657

RIY BY

Y1 0.594 0.601 0.605 0.623 0.642 0.645 0.652

Y2 0.542 0.550 0.554 0.574 0.595 0.599 0.607

Y3 0.558 0.565 0.569 0.588 0.608 0.612 0.619

WX1 BY

X1 0.675 0.681 0.684 0.698 0.713 0.715 0.721

WX2 BY

X2 0.754 0.759 0.761 0.774 0.787 0.789 0.794

WX3 BY

X3 0.717 0.722 0.724 0.737 0.750 0.753 0.758

WY1 BY

Y1 0.750 0.755 0.758 0.773 0.788 0.791 0.796

WY2 BY

Y2 0.785 0.791 0.793 0.808 0.823 0.825 0.831

WY3 BY

Y3 0.770 0.776 0.778 0.793 0.807 0.810 0.816

WX2 ON

WX1 0.110 0.120 0.126 0.153 0.180 0.185 0.195

WY1 -0.065 -0.056 -0.052 -0.029 -0.006 -0.001 0.008

WY2 ON

WY1 0.076 0.089 0.095 0.129 0.162 0.169 0.181

WX1 -0.069 -0.061 -0.057 -0.036 -0.014 -0.010 -0.002

WX3 ON

WX2 0.154 0.170 0.178 0.219 0.261 0.269 0.285

WY2 -0.082 -0.071 -0.066 -0.036 -0.007 -0.002 0.009

WY3 ON

WY2 0.087 0.103 0.111 0.153 0.195 0.203 0.218

WX2 -0.092 -0.082 -0.076 -0.048 -0.019 -0.014 -0.003

RIX ON

SEX -0.047 -0.037 -0.031 -0.004 0.024 0.029 0.040

EDU 0.527 0.535 0.540 0.562 0.585 0.589 0.598

RIY ON

SEX -0.229 -0.217 -0.211 -0.180 -0.149 -0.143 -0.132

EDU -0.219 -0.209 -0.204 -0.178 -0.152 -0.147 -0.137

WX2 ON

AG2 -0.196 -0.186 -0.181 -0.154 -0.126 -0.121 -0.111

CX2 0.081 0.090 0.094 0.117 0.140 0.145 0.153

MAR2 0.015 0.025 0.030 0.057 0.084 0.089 0.099

SMOK2 -0.119 -0.109 -0.104 -0.078 -0.052 -0.047 -0.038

DK2 -0.008 0.001 0.005 0.029 0.054 0.058 0.067

TPA2 -0.041 -0.032 -0.027 -0.002 0.023 0.028 0.037

CM2 -0.040 -0.030 -0.025 0.000 0.026 0.031 0.041

IAM2 -0.179 -0.165 -0.158 -0.121 -0.084 -0.077 -0.063

WY2 ON

AG2 -0.164 -0.154 -0.150 -0.125 -0.100 -0.095 -0.086

CX2 -0.114 -0.106 -0.103 -0.082 -0.062 -0.058 -0.051

MAR2 -0.106 -0.097 -0.092 -0.066 -0.041 -0.036 -0.027

SMOK2 -0.006 0.002 0.006 0.028 0.051 0.055 0.063

DK2 -0.077 -0.069 -0.065 -0.044 -0.023 -0.019 -0.011

TPA2 -0.005 0.003 0.007 0.029 0.051 0.055 0.063

CM2 0.137 0.146 0.151 0.177 0.203 0.208 0.218

IAM2 0.199 0.211 0.217 0.248 0.280 0.286 0.297

WX3 ON

AG3 -0.174 -0.163 -0.158 -0.129 -0.101 -0.095 -0.085

CX3 0.072 0.080 0.084 0.106 0.129 0.133 0.141

MAR3 0.009 0.019 0.024 0.049 0.074 0.079 0.088

SMOK3 -0.045 -0.036 -0.032 -0.008 0.016 0.020 0.029

DK3 0.016 0.024 0.028 0.050 0.072 0.077 0.085

IAM3 -0.206 -0.193 -0.186 -0.150 -0.114 -0.107 -0.094

TPA3 -0.014 -0.004 0.000 0.025 0.050 0.055 0.064

CM3 -0.031 -0.022 -0.017 0.008 0.032 0.037 0.046

WY3 ON

AG3 -0.063 -0.054 -0.049 -0.024 0.001 0.006 0.016

CX3 -0.125 -0.117 -0.113 -0.091 -0.069 -0.065 -0.057

MAR3 -0.101 -0.091 -0.086 -0.059 -0.033 -0.028 -0.018

SMOK3 -0.050 -0.041 -0.037 -0.012 0.012 0.016 0.025

DK3 -0.089 -0.081 -0.076 -0.054 -0.032 -0.028 -0.020

IAM3 0.220 0.233 0.239 0.274 0.308 0.314 0.327

TPA3 0.011 0.019 0.023 0.045 0.067 0.071 0.079

CM3 0.134 0.143 0.148 0.174 0.200 0.205 0.215

WX1 ON

AG1 -0.209 -0.199 -0.194 -0.167 -0.140 -0.134 -0.124

CX1 0.105 0.114 0.118 0.143 0.167 0.172 0.181

MAR1 0.014 0.024 0.029 0.055 0.081 0.086 0.096

SMOK1 -0.073 -0.063 -0.058 -0.032 -0.005 0.000 0.010

DK1 -0.003 0.007 0.012 0.037 0.063 0.068 0.077

TPA1 -0.072 -0.063 -0.059 -0.036 -0.013 -0.009 -0.001

IAM1 -0.162 -0.151 -0.145 -0.115 -0.086 -0.080 -0.069

CM1 -0.016 -0.007 -0.002 0.024 0.049 0.054 0.064

WY1 ON

AG1 -0.126 -0.116 -0.111 -0.085 -0.059 -0.054 -0.044

CX1 -0.129 -0.120 -0.116 -0.093 -0.071 -0.066 -0.058

MAR1 -0.146 -0.135 -0.130 -0.102 -0.074 -0.069 -0.059

SMOK1 0.007 0.016 0.021 0.046 0.070 0.075 0.084

DK1 -0.047 -0.038 -0.034 -0.009 0.015 0.020 0.029

TPA1 -0.019 -0.011 -0.006 0.016 0.038 0.042 0.051

IAM1 0.200 0.210 0.215 0.243 0.271 0.276 0.287

CM1 0.186 0.195 0.200 0.224 0.249 0.254 0.263

RIX WITH

RIY -0.276 -0.259 -0.251 -0.206 -0.161 -0.152 -0.135

WX1 0.000 0.000 0.000 0.000 0.000 0.000 0.000

WY1 0.000 0.000 0.000 0.000 0.000 0.000 0.000

WX1 WITH

WY1 -0.097 -0.085 -0.079 -0.047 -0.015 -0.009 0.003

RIY 0.000 0.000 0.000 0.000 0.000 0.000 0.000

WX2 WITH

WY2 -0.205 -0.191 -0.184 -0.148 -0.111 -0.104 -0.091

WX3 WITH

WY3 -0.175 -0.164 -0.159 -0.130 -0.102 -0.096 -0.086

RIY WITH

WY1 0.000 0.000 0.000 0.000 0.000 0.000 0.000

Intercepts

X1 2.710 2.780 2.816 3.004 3.192 3.228 3.299

X2 2.622 2.702 2.743 2.956 3.169 3.210 3.290

X3 2.654 2.734 2.776 2.991 3.207 3.248 3.328

Y1 2.087 2.158 2.194 2.383 2.573 2.609 2.680

Y2 2.309 2.382 2.419 2.613 2.806 2.844 2.916

Y3 1.830 1.905 1.943 2.142 2.341 2.379 2.454

Residual Variances

X1 0.000 0.000 0.000 0.000 0.000 0.000 0.000

X2 0.000 0.000 0.000 0.000 0.000 0.000 0.000

X3 0.000 0.000 0.000 0.000 0.000 0.000 0.000

Y1 0.000 0.000 0.000 0.000 0.000 0.000 0.000

Y2 0.000 0.000 0.000 0.000 0.000 0.000 0.000

Y3 0.000 0.000 0.000 0.000 0.000 0.000 0.000

RIX 0.646 0.656 0.660 0.685 0.709 0.714 0.723

RIY 0.899 0.905 0.908 0.923 0.939 0.941 0.947

WX1 0.898 0.904 0.907 0.921 0.936 0.939 0.944

WX2 0.847 0.855 0.858 0.878 0.897 0.901 0.908

WX3 0.803 0.812 0.817 0.842 0.866 0.871 0.880

WY1 0.832 0.839 0.843 0.861 0.879 0.882 0.889

WY2 0.803 0.811 0.815 0.837 0.859 0.863 0.871

WY3 0.756 0.766 0.770 0.795 0.820 0.825 0.834

TECHNICAL 1 OUTPUT

Use View Diagram under the Diagram menu in the Mplus Editor to view the diagram.

If running Mplus from the Mplus Diagrammer, the diagram opens automatically.

Diagram output

t:\mplus\shuj\ri-clpam-cx.dgm

Beginning Time: 16:31:33

Ending Time: 16:36:18

Elapsed Time: 00:04:45

//model 5i

HE MODEL ESTIMATION TERMINATED NORMALLY

MODEL FIT INFORMATION

Number of Free Parameters 117

Loglikelihood

H0 Value -108375.721

H1 Value -107993.749

Information Criteria

Akaike (AIC) 216985.441

Bayesian (BIC) 217785.924

Sample-Size Adjusted BIC 217414.126

(n* = (n + 2) / 24)

Chi-Square Test of Model Fit

Value 763.943

Degrees of Freedom 171

P-Value 0.0000

RMSEA (Root Mean Square Error Of Approximation)

Estimate 0.022

90 Percent C.I. 0.021 0.024

Probability RMSEA <= .05 1.000

CFI/TLI

CFI 0.958

TLI 0.934

Chi-Square Test of Model Fit for the Baseline Model

Value 14406.716

Degrees of Freedom 270

P-Value 0.0000

SRMR (Standardized Root Mean Square Residual)

Value 0.025

MODEL RESULTS

Two-Tailed

Estimate S.E. Est./S.E. P-Value

RIX BY

X1 1.000 0.000 999.000 999.000

X2 1.000 0.000 999.000 999.000

X3 1.000 0.000 999.000 999.000

RIINT BY

INT1 1.000 0.000 999.000 999.000

INT2 1.000 0.000 999.000 999.000

INT3 1.000 0.000 999.000 999.000

RIY BY

Y1 1.000 0.000 999.000 999.000

Y2 1.000 0.000 999.000 999.000

Y3 1.000 0.000 999.000 999.000

WX1 BY

X1 1.000 0.000 999.000 999.000

WX2 BY

X2 1.000 0.000 999.000 999.000

WX3 BY

X3 1.000 0.000 999.000 999.000

WINT1 BY

INT1 1.000 0.000 999.000 999.000

WINT2 BY

INT2 1.000 0.000 999.000 999.000

WINT3 BY

INT3 1.000 0.000 999.000 999.000

WY1 BY

Y1 1.000 0.000 999.000 999.000

WY2 BY

Y2 1.000 0.000 999.000 999.000

WY3 BY

Y3 1.000 0.000 999.000 999.000

WX2 ON

WX1 0.215 0.022 9.610 0.000

WY1 -0.046 0.008 -5.779 0.000

WINT1 0.629 0.078 8.052 0.000

WY2 ON

WY1 0.146 0.025 5.927 0.000

WX1 -0.182 0.025 -7.201 0.000

WINT1 -0.450 0.156 -2.878 0.004

WINT2 ON

WINT1 0.310 0.018 16.765 0.000

WX1 0.016 0.001 10.473 0.000

WY1 -0.003 0.001 -3.191 0.001

WX3 ON

WX2 0.215 0.022 9.610 0.000

WY2 -0.046 0.008 -5.779 0.000

WINT2 0.629 0.078 8.052 0.000

WY3 ON

WY2 0.146 0.025 5.927 0.000

WX2 -0.182 0.025 -7.201 0.000

WINT2 -0.450 0.156 -2.878 0.004

WINT3 ON

WINT2 0.310 0.018 16.765 0.000

WX2 0.016 0.001 10.473 0.000

WY2 -0.003 0.001 -3.191 0.001

RIX ON

SEX 0.014 0.072 0.195 0.845

EDU 1.780 0.049 36.024 0.000

RIY ON

SEX -1.300 0.131 -9.904 0.000

EDU -0.878 0.084 -10.413 0.000

RIINT ON

SEX -0.009 0.007 -1.205 0.228

EDU 0.093 0.004 21.804 0.000

WX2 ON

AG2 -0.047 0.005 -8.666 0.000

CX2 0.687 0.086 8.009 0.000

MAR2 0.489 0.146 3.345 0.001

SMOK2 -0.478 0.095 -5.006 0.000

DK2 0.146 0.083 1.757 0.079

TPA2 -0.020 0.073 -0.272 0.785

CM2 0.007 0.024 0.316 0.752

IAM2 -0.175 0.032 -5.517 0.000

WY2 ON

AG2 -0.078 0.009 -8.931 0.000

CX2 -0.851 0.130 -6.543 0.000

MAR2 -1.041 0.238 -4.369 0.000

SMOK2 0.307 0.142 2.159 0.031

DK2 -0.409 0.128 -3.183 0.001

TPA2 0.248 0.112 2.207 0.027

CM2 0.471 0.040 11.775 0.000

IAM2 0.626 0.049 12.830 0.000

WINT2 ON

AG2 -0.005 0.000 -11.008 0.000

CX2 0.097 0.010 9.768 0.000

MAR2 -0.003 0.012 -0.238 0.812

SMOK2 -0.003 0.009 -0.286 0.775

DK2 0.041 0.009 4.697 0.000

TPA2 -0.010 0.006 -1.523 0.128

CM2 -0.001 0.002 -0.273 0.785

IAM2 -0.004 0.001 -3.522 0.000

WX3 ON

AG3 -0.034 0.005 -6.589 0.000

CX3 0.489 0.069 7.125 0.000

MAR3 0.330 0.114 2.887 0.004

SMOK3 -0.036 0.079 -0.450 0.653

DK3 0.218 0.068 3.220 0.001

IAM3 -0.162 0.024 -6.691 0.000

TPA3 0.102 0.062 1.630 0.103

CM3 0.017 0.018 0.955 0.340

WY3 ON

AG3 -0.022 0.008 -2.670 0.008

CX3 -0.829 0.128 -6.500 0.000

MAR3 -0.796 0.227 -3.505 0.000

SMOK3 -0.164 0.153 -1.075 0.282

DK3 -0.487 0.128 -3.814 0.000

IAM3 0.570 0.045 12.672 0.000

TPA3 0.348 0.102 3.397 0.001

CM3 0.399 0.036 11.203 0.000

WINT3 ON

AG3 -0.020 0.001 -32.495 0.000

CX3 0.120 0.011 11.467 0.000

MAR3 -0.002 0.014 -0.135 0.893

SMOK3 -0.006 0.012 -0.506 0.613

DK3 0.047 0.011 4.380 0.000

IAM3 -0.008 0.002 -4.218 0.000

TPA3 0.003 0.008 0.441 0.660

CM3 0.010 0.002 3.941 0.000

WX1 ON

AG1 -0.043 0.004 -10.155 0.000

CX1 0.779 0.071 10.925 0.000

MAR1 0.432 0.127 3.416 0.001

SMOK1 -0.177 0.077 -2.293 0.022

DK1 0.155 0.069 2.231 0.026

TPA1 -0.115 0.040 -2.902 0.004

IAM1 -0.184 0.026 -7.099 0.000

CM1 0.029 0.024 1.230 0.219

WY1 ON

AG1 -0.042 0.008 -5.200 0.000

CX1 -0.998 0.128 -7.824 0.000

MAR1 -1.605 0.265 -6.066 0.000

SMOK1 0.448 0.139 3.212 0.001

DK1 -0.063 0.131 -0.477 0.633

TPA1 0.105 0.075 1.397 0.162

IAM1 0.707 0.052 13.664 0.000

CM1 0.676 0.046 14.803 0.000

WINT1 ON

AG1 -0.004 0.000 -11.350 0.000

CX1 0.117 0.009 13.504 0.000

MAR1 -0.025 0.011 -2.325 0.020

SMOK1 -0.013 0.008 -1.629 0.103

DK1 0.043 0.008 5.682 0.000

TPA1 -0.005 0.004 -1.266 0.206

IAM1 -0.001 0.001 -0.888 0.375

CM1 -0.003 0.002 -1.136 0.256

WX1 WITH

WY1 -1.055 0.167 -6.314 0.000

WINT1 0.068 0.008 8.541 0.000

RIX 0.000 0.000 999.000 999.000

RIY 0.000 0.000 999.000 999.000

RIINT 0.000 0.000 999.000 999.000

WY1 WITH

WINT1 -0.033 0.014 -2.427 0.015

RIX 0.000 0.000 999.000 999.000

RIY 0.000 0.000 999.000 999.000

WX2 WITH

WY2 -2.250 0.237 -9.479 0.000

WINT2 0.095 0.011 8.453 0.000

WY2 WITH

WINT2 -0.076 0.018 -4.186 0.000

WX3 WITH

WY3 -1.638 0.167 -9.797 0.000

WINT3 0.090 0.014 6.655 0.000

WY3 WITH

WINT3 -0.081 0.024 -3.329 0.001

RIINT WITH

WINT1 0.000 0.000 999.000 999.000

Intercepts

X1 9.436 0.345 27.327 0.000

X2 10.566 0.458 23.069 0.000

X3 9.996 0.431 23.198 0.000

Y1 13.228 0.651 20.322 0.000

Y2 15.834 0.732 21.634 0.000

Y3 12.724 0.724 17.563 0.000

INT1 -0.017 0.028 -0.610 0.542

INT2 0.184 0.041 4.509 0.000

INT3 1.413 0.051 27.576 0.000

Residual Variances

X1 0.000 0.000 999.000 999.000

X2 0.000 0.000 999.000 999.000

X3 0.000 0.000 999.000 999.000

Y1 0.000 0.000 999.000 999.000

Y2 0.000 0.000 999.000 999.000

Y3 0.000 0.000 999.000 999.000

INT1 0.000 0.000 999.000 999.000

INT2 0.000 0.000 999.000 999.000

INT3 0.000 0.000 999.000 999.000

RIX 2.710 0.130 20.770 0.000

RIINT 0.009 0.002 5.133 0.000

RIY 10.587 0.486 21.772 0.000

WX1 4.747 0.130 36.392 0.000

WX2 6.917 0.186 37.288 0.000

WX3 5.301 0.122 43.612 0.000

WINT1 0.060 0.002 25.944 0.000

WINT2 0.095 0.003 37.060 0.000

WINT3 0.162 0.002 77.351 0.000

WY1 16.464 0.515 31.981 0.000

WY2 19.939 0.629 31.703 0.000

WY3 17.627 0.471 37.391 0.000

STANDARDIZED MODEL RESULTS

STDYX Standardization

Two-Tailed

Estimate S.E. Est./S.E. P-Value

RIX BY

X1 0.645 0.011 61.166 0.000

X2 0.562 0.011 52.237 0.000

X3 0.600 0.011 55.966 0.000

RIINT BY

INT1 0.395 0.028 14.172 0.000

INT2 0.311 0.023 13.460 0.000

INT3 0.225 0.016 13.759 0.000

RIY BY

Y1 0.608 0.012 51.566 0.000

Y2 0.562 0.013 43.154 0.000

Y3 0.573 0.012 46.202 0.000

WX1 BY

X1 0.728 0.009 79.024 0.000

WX2 BY

X2 0.793 0.008 102.553 0.000

WX3 BY

X3 0.762 0.008 91.574 0.000

WINT1 BY

INT1 0.900 0.012 77.172 0.000

WINT2 BY

INT2 0.932 0.008 123.573 0.000

WINT3 BY

INT3 0.956 0.004 237.020 0.000

WY1 BY

Y1 0.786 0.009 86.166 0.000

WY2 BY

Y2 0.816 0.009 91.270 0.000

WY3 BY

Y3 0.804 0.009 90.770 0.000

WX2 ON

WX1 0.172 0.018 9.805 0.000

WY1 -0.071 0.013 -5.642 0.000

WINT1 0.056 0.007 7.722 0.000

WY2 ON

WY1 0.130 0.021 6.095 0.000

WX1 -0.084 0.012 -6.950 0.000

WINT1 -0.023 0.008 -2.870 0.004

WINT2 ON

WINT1 0.236 0.015 15.531 0.000

WX1 0.106 0.010 10.564 0.000

WY1 -0.034 0.011 -3.194 0.001

WX3 ON

WX2 0.239 0.025 9.391 0.000

WY2 -0.088 0.015 -5.777 0.000

WINT2 0.082 0.010 7.970 0.000

WY3 ON

WY2 0.151 0.026 5.807 0.000

WX2 -0.109 0.015 -7.157 0.000

WINT2 -0.032 0.011 -2.877 0.004

WINT3 ON

WINT2 0.218 0.014 15.860 0.000

WX2 0.093 0.009 10.331 0.000

WY2 -0.027 0.008 -3.185 0.001

RIX ON

SEX 0.003 0.018 0.195 0.845

EDU 0.577 0.015 39.257 0.000

RIY ON

SEX -0.190 0.019 -9.884 0.000

EDU -0.169 0.016 -10.494 0.000

RIINT ON

SEX -0.040 0.034 -1.177 0.239

EDU 0.543 0.044 12.425 0.000

WX2 ON

AG2 -0.140 0.016 -8.679 0.000

CX2 0.110 0.014 8.049 0.000

MAR2 0.053 0.016 3.346 0.001

SMOK2 -0.077 0.015 -5.003 0.000

DK2 0.025 0.014 1.757 0.079

TPA2 -0.004 0.015 -0.273 0.785

CM2 0.005 0.015 0.316 0.752

IAM2 -0.119 0.022 -5.518 0.000

WY2 ON

AG2 -0.135 0.015 -8.919 0.000

CX2 -0.079 0.012 -6.466 0.000

MAR2 -0.066 0.015 -4.351 0.000

SMOK2 0.029 0.013 2.153 0.031

DK2 -0.041 0.013 -3.206 0.001

TPA2 0.029 0.013 2.217 0.027

CM2 0.177 0.016 11.369 0.000

IAM2 0.247 0.019 12.948 0.000

WINT2 ON

AG2 -0.132 0.012 -11.236 0.000

CX2 0.132 0.013 10.035 0.000

MAR2 -0.003 0.011 -0.239 0.811

SMOK2 -0.003 0.012 -0.286 0.775

DK2 0.059 0.012 4.742 0.000

TPA2 -0.017 0.011 -1.528 0.127

CM2 -0.003 0.012 -0.273 0.785

IAM2 -0.025 0.007 -3.592 0.000

WX3 ON

AG3 -0.111 0.017 -6.597 0.000

CX3 0.092 0.013 7.159 0.000

MAR3 0.043 0.015 2.885 0.004

SMOK3 -0.006 0.014 -0.449 0.653

DK3 0.042 0.013 3.211 0.001

IAM3 -0.140 0.021 -6.609 0.000

TPA3 0.024 0.015 1.635 0.102

CM3 0.014 0.014 0.956 0.339

WY3 ON

AG3 -0.040 0.015 -2.654 0.008

CX3 -0.084 0.013 -6.481 0.000

MAR3 -0.055 0.016 -3.514 0.000

SMOK3 -0.016 0.015 -1.075 0.283

DK3 -0.050 0.013 -3.809 0.000

IAM3 0.266 0.021 12.857 0.000

TPA3 0.045 0.013 3.395 0.001

CM3 0.172 0.016 10.995 0.000

WINT3 ON

AG3 -0.359 0.011 -32.147 0.000

CX3 0.123 0.011 11.416 0.000

MAR3 -0.001 0.010 -0.135 0.893

SMOK3 -0.006 0.011 -0.507 0.612

DK3 0.049 0.011 4.377 0.000

IAM3 -0.039 0.009 -4.132 0.000

TPA3 0.004 0.010 0.441 0.659

CM3 0.042 0.011 3.947 0.000

WX1 ON

AG1 -0.162 0.016 -10.216 0.000

CX1 0.159 0.014 11.174 0.000

MAR1 0.053 0.015 3.426 0.001

SMOK1 -0.036 0.016 -2.292 0.022

DK1 0.034 0.015 2.225 0.026

TPA1 -0.039 0.014 -2.910 0.004

IAM1 -0.123 0.017 -7.174 0.000

CM1 0.018 0.015 1.227 0.220

WY1 ON

AG1 -0.081 0.016 -5.182 0.000

CX1 -0.106 0.013 -7.902 0.000

MAR1 -0.101 0.017 -6.065 0.000

SMOK1 0.047 0.015 3.225 0.001

DK1 -0.007 0.015 -0.477 0.634

TPA1 0.019 0.013 1.400 0.161

IAM1 0.246 0.017 14.894 0.000

CM1 0.221 0.015 14.989 0.000

WINT1 ON

AG1 -0.141 0.012 -12.265 0.000

CX1 0.213 0.014 15.127 0.000

MAR1 -0.027 0.012 -2.326 0.020

SMOK1 -0.024 0.015 -1.632 0.103

DK1 0.084 0.015 5.753 0.000

TPA1 -0.015 0.012 -1.267 0.205

IAM1 -0.005 0.006 -0.898 0.369

CM1 -0.014 0.012 -1.142 0.253

WX1 WITH

WY1 -0.119 0.018 -6.639 0.000

WINT1 0.127 0.014 9.094 0.000

RIX 0.000 0.000 0.000 1.000

RIY 0.000 0.000 0.000 1.000

RIINT 0.000 0.000 0.000 1.000

WY1 WITH

WINT1 -0.033 0.014 -2.427 0.015

RIX 0.000 0.000 0.000 1.000

RIY 0.000 0.000 0.000 1.000

WX2 WITH

WY2 -0.192 0.021 -9.207 0.000

WINT2 0.117 0.014 8.429 0.000

WY2 WITH

WINT2 -0.055 0.013 -4.182 0.000

WX3 WITH

WY3 -0.169 0.017 -10.067 0.000

WINT3 0.097 0.014 6.730 0.000

WY3 WITH

WINT3 -0.048 0.014 -3.317 0.001

RIINT WITH

WINT1 0.000 0.000 0.000 1.000

Intercepts

X1 3.018 0.115 26.262 0.000

X2 2.947 0.130 22.624 0.000

X3 2.972 0.132 22.554 0.000

Y1 2.372 0.115 20.576 0.000

Y2 2.628 0.119 22.141 0.000

Y3 2.152 0.121 17.771 0.000

INT1 -0.060 0.099 -0.609 0.542

INT2 0.513 0.113 4.548 0.000

INT3 2.846 0.102 27.769 0.000

Residual Variances

X1 0.000 0.000 0.000 1.000

X2 0.000 0.000 0.000 1.000

X3 0.000 0.000 0.000 1.000

Y1 0.000 0.000 0.000 1.000

Y2 0.000 0.000 0.000 1.000

Y3 0.000 0.000 0.000 1.000

INT1 0.000 0.000 0.000 1.000

INT2 0.000 0.000 0.000 1.000

INT3 0.000 0.000 0.000 1.000

RIX 0.666 0.016 40.866 0.000

RIINT 0.712 0.048 14.765 0.000

RIY 0.922 0.010 94.629 0.000

WX1 0.915 0.009 105.360 0.000

WX2 0.856 0.012 72.076 0.000

WX3 0.807 0.015 54.820 0.000

WINT1 0.926 0.007 130.386 0.000

WINT2 0.847 0.010 88.721 0.000

WINT3 0.721 0.009 84.247 0.000

WY1 0.858 0.011 79.092 0.000

WY2 0.824 0.013 62.287 0.000

WY3 0.780 0.015 52.459 0.000

R-SQUARE

Observed Two-Tailed

Variable Estimate S.E. Est./S.E. P-Value

X1 1.000 0.000 999.000 999.000

X2 1.000 0.000 999.000 999.000

X3 1.000 0.000 999.000 999.000

Y1 1.000 0.000 999.000 999.000

Y2 1.000 0.000 999.000 999.000

Y3 1.000 0.000 999.000 999.000

INT1 1.000 0.000 999.000 999.000

INT2 1.000 0.000 999.000 999.000

INT3 1.000 0.000 999.000 999.000

Latent Two-Tailed

Variable Estimate S.E. Est./S.E. P-Value

RIX 0.334 0.016 20.450 0.000

RIINT 0.288 0.048 5.974 0.000

RIY 0.078 0.010 7.982 0.000

WX1 0.085 0.009 9.726 0.000

WX2 0.144 0.012 12.148 0.000

WX3 0.193 0.015 13.142 0.000

WINT1 0.074 0.007 10.480 0.000

WINT2 0.153 0.010 15.999 0.000

WINT3 0.279 0.009 32.552 0.000

WY1 0.142 0.011 13.097 0.000

WY2 0.176 0.013 13.293 0.000

WY3 0.220 0.015 14.832 0.000

TOTAL, TOTAL INDIRECT, SPECIFIC INDIRECT, AND DIRECT EFFECTS

Two-Tailed

Estimate S.E. Est./S.E. P-Value

Effects from WX1 to WY3

Total -0.073 0.010 -7.040 0.000

Total indirect -0.073 0.010 -7.040 0.000

Specific indirect 1

WY3

WX2

WX1 -0.039 0.007 -5.640 0.000

Specific indirect 2

WY3

WINT2

WX1 -0.007 0.002 -2.830 0.005

Specific indirect 3

WY3

WY2

WX1 -0.027 0.005 -4.976 0.000

Effects from WY1 to WX3

Total -0.018 0.003 -5.599 0.000

Total indirect -0.018 0.003 -5.599 0.000

Specific indirect 1

WX3

WX2

WY1 -0.010 0.002 -5.333 0.000

Specific indirect 2

WX3

WINT2

WY1 -0.002 0.001 -2.978 0.003

Specific indirect 3

WX3

WY2

WY1 -0.007 0.002 -3.921 0.000

STANDARDIZED TOTAL, TOTAL INDIRECT, SPECIFIC INDIRECT, AND DIRECT EFFECTS

STDYX Standardization

Two-Tailed

Estimate S.E. Est./S.E. P-Value

Effects from WX1 to WY3

Total -0.035 0.005 -6.935 0.000

Total indirect -0.035 0.005 -6.935 0.000

Specific indirect 1

WY3

WX2

WX1 -0.019 0.003 -5.454 0.000

Specific indirect 2

WY3

WINT2

WX1 -0.003 0.001 -2.821 0.005

Specific indirect 3

WY3

WY2

WX1 -0.013 0.003 -5.094 0.000

Effects from WY1 to WX3

Total -0.031 0.006 -5.593 0.000

Total indirect -0.031 0.006 -5.593 0.000

Specific indirect 1

WX3

WX2

WY1 -0.017 0.003 -5.439 0.000

Specific indirect 2

WX3

WINT2

WY1 -0.003 0.001 -2.984 0.003

Specific indirect 3

WX3

WY2

WY1 -0.012 0.003 -3.851 0.000

CONFIDENCE INTERVALS OF MODEL RESULTS

Lower .5% Lower 2.5% Lower 5% Estimate Upper 5% Upper 2.5% Upper .5%

RIX BY

X1 1.000 1.000 1.000 1.000 1.000 1.000 1.000

X2 1.000 1.000 1.000 1.000 1.000 1.000 1.000

X3 1.000 1.000 1.000 1.000 1.000 1.000 1.000

RIINT BY

INT1 1.000 1.000 1.000 1.000 1.000 1.000 1.000

INT2 1.000 1.000 1.000 1.000 1.000 1.000 1.000

INT3 1.000 1.000 1.000 1.000 1.000 1.000 1.000

RIY BY

Y1 1.000 1.000 1.000 1.000 1.000 1.000 1.000

Y2 1.000 1.000 1.000 1.000 1.000 1.000 1.000

Y3 1.000 1.000 1.000 1.000 1.000 1.000 1.000

WX1 BY

X1 1.000 1.000 1.000 1.000 1.000 1.000 1.000

WX2 BY

X2 1.000 1.000 1.000 1.000 1.000 1.000 1.000

WX3 BY

X3 1.000 1.000 1.000 1.000 1.000 1.000 1.000

WINT1 BY

INT1 1.000 1.000 1.000 1.000 1.000 1.000 1.000

WINT2 BY

INT2 1.000 1.000 1.000 1.000 1.000 1.000 1.000

WINT3 BY

INT3 1.000 1.000 1.000 1.000 1.000 1.000 1.000

WY1 BY

Y1 1.000 1.000 1.000 1.000 1.000 1.000 1.000

WY2 BY

Y2 1.000 1.000 1.000 1.000 1.000 1.000 1.000

WY3 BY

Y3 1.000 1.000 1.000 1.000 1.000 1.000 1.000

WX2 ON

WX1 0.158 0.171 0.178 0.215 0.252 0.259 0.273

WY1 -0.067 -0.062 -0.059 -0.046 -0.033 -0.030 -0.026

WINT1 0.428 0.476 0.501 0.629 0.758 0.782 0.830

WY2 ON

WY1 0.083 0.098 0.106 0.146 0.187 0.195 0.210

WX1 -0.247 -0.232 -0.224 -0.182 -0.140 -0.132 -0.117

WINT1 -0.852 -0.756 -0.706 -0.450 -0.193 -0.143 -0.047

WINT2 ON

WINT1 0.262 0.274 0.280 0.310 0.340 0.346 0.358

WX1 0.012 0.013 0.013 0.016 0.018 0.018 0.019

WY1 -0.005 -0.004 -0.004 -0.003 -0.001 -0.001 -0.001

WX3 ON

WX2 0.158 0.171 0.178 0.215 0.252 0.259 0.273

WY2 -0.067 -0.062 -0.059 -0.046 -0.033 -0.030 -0.026

WINT2 0.428 0.476 0.501 0.629 0.758 0.782 0.830

WY3 ON

WY2 0.083 0.098 0.106 0.146 0.187 0.195 0.210

WX2 -0.247 -0.232 -0.224 -0.182 -0.140 -0.132 -0.117

WINT2 -0.852 -0.756 -0.706 -0.450 -0.193 -0.143 -0.047

WINT3 ON

WINT2 0.262 0.274 0.280 0.310 0.340 0.346 0.358

WX2 0.012 0.013 0.013 0.016 0.018 0.018 0.019

WY2 -0.005 -0.004 -0.004 -0.003 -0.001 -0.001 -0.001

RIX ON

SEX -0.172 -0.128 -0.105 0.014 0.133 0.156 0.201

EDU 1.653 1.684 1.699 1.780 1.862 1.877 1.908

RIY ON

SEX -1.638 -1.557 -1.515 -1.300 -1.084 -1.042 -0.962

EDU -1.095 -1.043 -1.017 -0.878 -0.739 -0.713 -0.661

RIINT ON

SEX -0.028 -0.024 -0.021 -0.009 0.003 0.006 0.010

EDU 0.082 0.085 0.086 0.093 0.100 0.101 0.104

WX2 ON

AG2 -0.061 -0.058 -0.056 -0.047 -0.038 -0.036 -0.033

CX2 0.466 0.519 0.546 0.687 0.828 0.855 0.908

MAR2 0.112 0.202 0.248 0.489 0.729 0.775 0.865

SMOK2 -0.723 -0.665 -0.635 -0.478 -0.321 -0.291 -0.232

DK2 -0.068 -0.017 0.009 0.146 0.283 0.309 0.360

TPA2 -0.209 -0.164 -0.140 -0.020 0.101 0.124 0.169

CM2 -0.054 -0.039 -0.032 0.007 0.047 0.054 0.069

IAM2 -0.256 -0.237 -0.227 -0.175 -0.123 -0.113 -0.093

WY2 ON

AG2 -0.101 -0.096 -0.093 -0.078 -0.064 -0.061 -0.056

CX2 -1.186 -1.105 -1.065 -0.851 -0.637 -0.596 -0.516

MAR2 -1.655 -1.508 -1.433 -1.041 -0.649 -0.574 -0.427

SMOK2 -0.059 0.028 0.073 0.307 0.541 0.586 0.674

DK2 -0.740 -0.661 -0.620 -0.409 -0.198 -0.157 -0.078

TPA2 -0.041 0.028 0.063 0.248 0.432 0.468 0.537

CM2 0.368 0.392 0.405 0.471 0.537 0.549 0.574

IAM2 0.501 0.531 0.546 0.626 0.707 0.722 0.752

WINT2 ON

AG2 -0.006 -0.006 -0.006 -0.005 -0.004 -0.004 -0.004

CX2 0.071 0.077 0.080 0.097 0.113 0.116 0.122

MAR2 -0.033 -0.026 -0.022 -0.003 0.016 0.020 0.027

SMOK2 -0.025 -0.020 -0.017 -0.003 0.012 0.015 0.020

DK2 0.018 0.024 0.026 0.041 0.055 0.057 0.063

TPA2 -0.026 -0.022 -0.020 -0.010 0.001 0.003 0.007

CM2 -0.006 -0.005 -0.004 -0.001 0.003 0.004 0.005

IAM2 -0.007 -0.007 -0.006 -0.004 -0.002 -0.002 -0.001

WX3 ON

AG3 -0.047 -0.044 -0.042 -0.034 -0.025 -0.024 -0.021

CX3 0.312 0.354 0.376 0.489 0.601 0.623 0.665

MAR3 0.036 0.106 0.142 0.330 0.519 0.555 0.625

SMOK3 -0.240 -0.191 -0.166 -0.036 0.095 0.120 0.168

DK3 0.044 0.085 0.107 0.218 0.329 0.351 0.392

IAM3 -0.224 -0.209 -0.201 -0.162 -0.122 -0.114 -0.099

TPA3 -0.059 -0.021 -0.001 0.102 0.204 0.224 0.263

CM3 -0.029 -0.018 -0.012 0.017 0.047 0.053 0.064

WY3 ON

AG3 -0.044 -0.039 -0.036 -0.022 -0.009 -0.006 -0.001

CX3 -1.158 -1.079 -1.039 -0.829 -0.619 -0.579 -0.501

MAR3 -1.381 -1.241 -1.169 -0.796 -0.422 -0.351 -0.211

SMOK3 -0.557 -0.463 -0.415 -0.164 0.087 0.135 0.229

DK3 -0.816 -0.737 -0.697 -0.487 -0.277 -0.237 -0.158

IAM3 0.454 0.482 0.496 0.570 0.644 0.658 0.686

TPA3 0.084 0.147 0.179 0.348 0.516 0.548 0.612

CM3 0.307 0.329 0.340 0.399 0.457 0.468 0.490

WINT3 ON

AG3 -0.022 -0.021 -0.021 -0.020 -0.019 -0.019 -0.019

CX3 0.093 0.100 0.103 0.120 0.138 0.141 0.148

MAR3 -0.038 -0.030 -0.025 -0.002 0.021 0.026 0.035

SMOK3 -0.037 -0.029 -0.026 -0.006 0.014 0.017 0.025

DK3 0.020 0.026 0.030 0.047 0.065 0.069 0.075

IAM3 -0.013 -0.012 -0.011 -0.008 -0.005 -0.004 -0.003

TPA3 -0.017 -0.012 -0.010 0.003 0.017 0.019 0.024

CM3 0.003 0.005 0.006 0.010 0.014 0.015 0.016

WX1 ON

AG1 -0.055 -0.052 -0.051 -0.043 -0.036 -0.035 -0.032

CX1 0.595 0.639 0.662 0.779 0.896 0.919 0.963

MAR1 0.106 0.184 0.224 0.432 0.641 0.681 0.759

SMOK1 -0.375 -0.328 -0.303 -0.177 -0.050 -0.026 0.022

DK1 -0.024 0.019 0.041 0.155 0.269 0.291 0.334

TPA1 -0.217 -0.193 -0.180 -0.115 -0.050 -0.037 -0.013

IAM1 -0.250 -0.234 -0.226 -0.184 -0.141 -0.133 -0.117

CM1 -0.032 -0.017 -0.010 0.029 0.068 0.076 0.090

WY1 ON

AG1 -0.063 -0.058 -0.055 -0.042 -0.029 -0.026 -0.021

CX1 -1.327 -1.248 -1.208 -0.998 -0.788 -0.748 -0.669

MAR1 -2.286 -2.124 -2.040 -1.605 -1.170 -1.086 -0.923

SMOK1 0.089 0.175 0.218 0.448 0.677 0.721 0.807

DK1 -0.401 -0.320 -0.279 -0.063 0.153 0.195 0.276

TPA1 -0.088 -0.042 -0.019 0.105 0.228 0.252 0.298

IAM1 0.574 0.606 0.622 0.707 0.792 0.809 0.841

CM1 0.558 0.586 0.601 0.676 0.751 0.765 0.793

WINT1 ON

AG1 -0.005 -0.005 -0.005 -0.004 -0.004 -0.004 -0.003

CX1 0.094 0.100 0.102 0.117 0.131 0.134 0.139

MAR1 -0.052 -0.046 -0.042 -0.025 -0.007 -0.004 0.003

SMOK1 -0.034 -0.029 -0.026 -0.013 0.000 0.003 0.008

DK1 0.024 0.028 0.031 0.043 0.056 0.058 0.063

TPA1 -0.015 -0.012 -0.011 -0.005 0.001 0.003 0.005

IAM1 -0.003 -0.003 -0.003 -0.001 0.001 0.001 0.002

CM1 -0.008 -0.007 -0.006 -0.003 0.001 0.002 0.003

WX1 WITH

WY1 -1.485 -1.383 -1.330 -1.055 -0.780 -0.728 -0.625

WINT1 0.047 0.052 0.055 0.068 0.081 0.083 0.088

RIX 0.000 0.000 0.000 0.000 0.000 0.000 0.000

RIY 0.000 0.000 0.000 0.000 0.000 0.000 0.000

RIINT 0.000 0.000 0.000 0.000 0.000 0.000 0.000

WY1 WITH

WINT1 -0.068 -0.060 -0.056 -0.033 -0.011 -0.006 0.002

RIX 0.000 0.000 0.000 0.000 0.000 0.000 0.000

RIY 0.000 0.000 0.000 0.000 0.000 0.000 0.000

WX2 WITH

WY2 -2.861 -2.715 -2.640 -2.250 -1.859 -1.784 -1.638

WINT2 0.066 0.073 0.076 0.095 0.113 0.117 0.124

WY2 WITH

WINT2 -0.122 -0.111 -0.105 -0.076 -0.046 -0.040 -0.029

WX3 WITH

WY3 -2.069 -1.966 -1.913 -1.638 -1.363 -1.310 -1.207

WINT3 0.055 0.064 0.068 0.090 0.112 0.117 0.125

WY3 WITH

WINT3 -0.144 -0.129 -0.121 -0.081 -0.041 -0.033 -0.018

RIINT WITH

WINT1 0.000 0.000 0.000 0.000 0.000 0.000 0.000

Intercepts

X1 8.547 8.760 8.868 9.436 10.004 10.113 10.326

X2 9.386 9.668 9.812 10.566 11.319 11.464 11.746

X3 8.886 9.152 9.288 9.996 10.705 10.841 11.106

Y1 11.551 11.952 12.157 13.228 14.299 14.504 14.905

Y2 13.949 14.399 14.630 15.834 17.038 17.268 17.719

Y3 10.858 11.304 11.532 12.724 13.916 14.144 14.590

INT1 -0.089 -0.072 -0.063 -0.017 0.029 0.038 0.055

INT2 0.079 0.104 0.117 0.184 0.251 0.264 0.290

INT3 1.281 1.313 1.329 1.413 1.498 1.514 1.546

Residual Variances

X1 0.000 0.000 0.000 0.000 0.000 0.000 0.000

X2 0.000 0.000 0.000 0.000 0.000 0.000 0.000

X3 0.000 0.000 0.000 0.000 0.000 0.000 0.000

Y1 0.000 0.000 0.000 0.000 0.000 0.000 0.000

Y2 0.000 0.000 0.000 0.000 0.000 0.000 0.000

Y3 0.000 0.000 0.000 0.000 0.000 0.000 0.000

INT1 0.000 0.000 0.000 0.000 0.000 0.000 0.000

INT2 0.000 0.000 0.000 0.000 0.000 0.000 0.000

INT3 0.000 0.000 0.000 0.000 0.000 0.000 0.000

RIX 2.374 2.454 2.495 2.710 2.925 2.966 3.046

RIINT 0.004 0.005 0.006 0.009 0.012 0.012 0.013

RIY 9.335 9.634 9.787 10.587 11.387 11.540 11.840

WX1 4.411 4.491 4.532 4.747 4.962 5.003 5.083

WX2 6.439 6.554 6.612 6.917 7.222 7.281 7.395

WX3 4.988 5.063 5.101 5.301 5.501 5.539 5.614

WINT1 0.054 0.055 0.056 0.060 0.064 0.065 0.066

WINT2 0.088 0.090 0.091 0.095 0.099 0.100 0.101

WINT3 0.157 0.158 0.159 0.162 0.166 0.167 0.168

WY1 15.138 15.455 15.617 16.464 17.311 17.473 17.790

WY2 18.319 18.706 18.905 19.939 20.974 21.172 21.559

WY3 16.413 16.703 16.851 17.627 18.402 18.551 18.841

CONFIDENCE INTERVALS OF STANDARDIZED MODEL RESULTS

STDYX Standardization

Lower .5% Lower 2.5% Lower 5% Estimate Upper 5% Upper 2.5% Upper .5%

RIX BY

X1 0.618 0.624 0.628 0.645 0.662 0.666 0.672

X2 0.535 0.541 0.545 0.562 0.580 0.583 0.590

X3 0.572 0.579 0.582 0.600 0.617 0.621 0.627

RIINT BY

INT1 0.323 0.340 0.349 0.395 0.441 0.450 0.467

INT2 0.252 0.266 0.273 0.311 0.349 0.357 0.371

INT3 0.183 0.193 0.198 0.225 0.252 0.257 0.267

RIY BY

Y1 0.577 0.585 0.588 0.608 0.627 0.631 0.638

Y2 0.529 0.537 0.541 0.562 0.584 0.588 0.596

Y3 0.541 0.549 0.553 0.573 0.593 0.597 0.605

WX1 BY

X1 0.705 0.710 0.713 0.728 0.744 0.746 0.752

WX2 BY

X2 0.773 0.778 0.780 0.793 0.806 0.808 0.813

WX3 BY

X3 0.741 0.746 0.748 0.762 0.776 0.778 0.784

WINT1 BY

INT1 0.870 0.877 0.880 0.900 0.919 0.922 0.930

WINT2 BY

INT2 0.913 0.917 0.920 0.932 0.944 0.947 0.951

WINT3 BY

INT3 0.945 0.948 0.949 0.956 0.962 0.963 0.966

WY1 BY

Y1 0.762 0.768 0.771 0.786 0.801 0.803 0.809

WY2 BY

Y2 0.793 0.799 0.802 0.816 0.831 0.834 0.839

WY3 BY

Y3 0.781 0.787 0.790 0.804 0.819 0.822 0.827

WX2 ON

WX1 0.127 0.138 0.143 0.172 0.201 0.207 0.218

WY1 -0.103 -0.096 -0.092 -0.071 -0.050 -0.046 -0.039

WINT1 0.038 0.042 0.044 0.056 0.068 0.071 0.075

WY2 ON

WY1 0.075 0.088 0.095 0.130 0.165 0.172 0.185

WX1 -0.115 -0.108 -0.104 -0.084 -0.064 -0.060 -0.053

WINT1 -0.044 -0.039 -0.037 -0.023 -0.010 -0.007 -0.002

WINT2 ON

WINT1 0.197 0.206 0.211 0.236 0.261 0.266 0.275

WX1 0.080 0.086 0.089 0.106 0.122 0.125 0.131

WY1 -0.061 -0.055 -0.051 -0.034 -0.016 -0.013 -0.007

WX3 ON

WX2 0.173 0.189 0.197 0.239 0.280 0.288 0.304

WY2 -0.128 -0.118 -0.113 -0.088 -0.063 -0.058 -0.049

WINT2 0.056 0.062 0.065 0.082 0.099 0.102 0.109

WY3 ON

WY2 0.084 0.100 0.108 0.151 0.194 0.202 0.218

WX2 -0.148 -0.139 -0.134 -0.109 -0.084 -0.079 -0.070

WINT2 -0.060 -0.053 -0.050 -0.032 -0.014 -0.010 -0.003

WINT3 ON

WINT2 0.183 0.191 0.196 0.218 0.241 0.245 0.254

WX2 0.070 0.075 0.078 0.093 0.108 0.111 0.116

WY2 -0.049 -0.043 -0.041 -0.027 -0.013 -0.010 -0.005

RIX ON

SEX -0.042 -0.031 -0.026 0.003 0.033 0.038 0.049

EDU 0.539 0.548 0.553 0.577 0.601 0.606 0.615

RIY ON

SEX -0.240 -0.228 -0.222 -0.190 -0.159 -0.153 -0.141

EDU -0.211 -0.201 -0.196 -0.169 -0.143 -0.138 -0.128

RIINT ON

SEX -0.128 -0.107 -0.096 -0.040 0.016 0.027 0.048

EDU 0.431 0.458 0.471 0.543 0.615 0.629 0.656

WX2 ON

AG2 -0.181 -0.171 -0.166 -0.140 -0.113 -0.108 -0.098

CX2 0.075 0.084 0.088 0.110 0.133 0.137 0.146

MAR2 0.012 0.022 0.027 0.053 0.080 0.085 0.095

SMOK2 -0.117 -0.107 -0.103 -0.077 -0.052 -0.047 -0.037

DK2 -0.012 -0.003 0.002 0.025 0.049 0.053 0.062

TPA2 -0.042 -0.033 -0.029 -0.004 0.020 0.025 0.034

CM2 -0.035 -0.025 -0.021 0.005 0.030 0.035 0.045

IAM2 -0.175 -0.162 -0.155 -0.119 -0.084 -0.077 -0.064

WY2 ON

AG2 -0.174 -0.165 -0.160 -0.135 -0.110 -0.105 -0.096

CX2 -0.110 -0.103 -0.099 -0.079 -0.059 -0.055 -0.048

MAR2 -0.105 -0.095 -0.091 -0.066 -0.041 -0.036 -0.027

SMOK2 -0.006 0.003 0.007 0.029 0.051 0.055 0.063

DK2 -0.073 -0.065 -0.061 -0.041 -0.020 -0.016 -0.008

TPA2 -0.005 0.003 0.008 0.029 0.051 0.055 0.063

CM2 0.137 0.147 0.152 0.177 0.203 0.208 0.217

IAM2 0.198 0.210 0.216 0.247 0.279 0.285 0.297

WINT2 ON

AG2 -0.162 -0.155 -0.151 -0.132 -0.112 -0.109 -0.101

CX2 0.098 0.106 0.110 0.132 0.154 0.158 0.166

MAR2 -0.031 -0.024 -0.020 -0.003 0.015 0.019 0.025

SMOK2 -0.034 -0.027 -0.023 -0.003 0.016 0.020 0.027

DK2 0.027 0.035 0.039 0.059 0.080 0.084 0.091

TPA2 -0.045 -0.039 -0.035 -0.017 0.001 0.005 0.012

CM2 -0.035 -0.028 -0.024 -0.003 0.017 0.021 0.028

IAM2 -0.043 -0.038 -0.036 -0.025 -0.013 -0.011 -0.007

WX3 ON

AG3 -0.155 -0.144 -0.139 -0.111 -0.083 -0.078 -0.068

CX3 0.059 0.067 0.071 0.092 0.113 0.117 0.125

MAR3 0.005 0.014 0.018 0.043 0.067 0.072 0.081

SMOK3 -0.042 -0.034 -0.029 -0.006 0.017 0.021 0.030

DK3 0.008 0.016 0.020 0.042 0.063 0.067 0.075

IAM3 -0.195 -0.181 -0.175 -0.140 -0.105 -0.098 -0.085

TPA3 -0.014 -0.005 0.000 0.024 0.049 0.053 0.062

CM3 -0.023 -0.014 -0.010 0.014 0.037 0.042 0.051

WY3 ON

AG3 -0.079 -0.070 -0.065 -0.040 -0.015 -0.010 -0.001

CX3 -0.118 -0.110 -0.106 -0.084 -0.063 -0.059 -0.051

MAR3 -0.096 -0.086 -0.081 -0.055 -0.029 -0.024 -0.015

SMOK3 -0.053 -0.044 -0.040 -0.016 0.008 0.013 0.022

DK3 -0.084 -0.076 -0.072 -0.050 -0.029 -0.024 -0.016

IAM3 0.213 0.225 0.232 0.266 0.300 0.307 0.319

TPA3 0.011 0.019 0.023 0.045 0.066 0.070 0.078

CM3 0.131 0.141 0.146 0.172 0.197 0.202 0.212

WINT3 ON

AG3 -0.388 -0.381 -0.377 -0.359 -0.341 -0.337 -0.330

CX3 0.095 0.102 0.105 0.123 0.140 0.144 0.150

MAR3 -0.027 -0.021 -0.018 -0.001 0.015 0.018 0.024

SMOK3 -0.035 -0.028 -0.025 -0.006 0.013 0.017 0.024

DK3 0.020 0.027 0.031 0.049 0.068 0.071 0.078

IAM3 -0.063 -0.057 -0.054 -0.039 -0.023 -0.020 -0.015

TPA3 -0.022 -0.015 -0.012 0.004 0.021 0.024 0.031

CM3 0.015 0.021 0.025 0.042 0.060 0.063 0.070

WX1 ON

AG1 -0.202 -0.193 -0.188 -0.162 -0.136 -0.131 -0.121

CX1 0.122 0.131 0.135 0.159 0.182 0.186 0.195

MAR1 0.013 0.022 0.027 0.053 0.078 0.083 0.092

SMOK1 -0.077 -0.067 -0.062 -0.036 -0.010 -0.005 0.004

DK1 -0.005 0.004 0.009 0.034 0.058 0.063 0.072

TPA1 -0.074 -0.066 -0.062 -0.039 -0.017 -0.013 -0.005

IAM1 -0.167 -0.156 -0.151 -0.123 -0.095 -0.089 -0.079

CM1 -0.020 -0.011 -0.006 0.018 0.043 0.047 0.057

WY1 ON

AG1 -0.121 -0.112 -0.107 -0.081 -0.055 -0.050 -0.041

CX1 -0.140 -0.132 -0.128 -0.106 -0.084 -0.079 -0.071

MAR1 -0.144 -0.134 -0.129 -0.101 -0.074 -0.069 -0.058

SMOK1 0.010 0.019 0.023 0.047 0.072 0.076 0.085

DK1 -0.045 -0.036 -0.031 -0.007 0.017 0.022 0.031

TPA1 -0.016 -0.007 -0.003 0.019 0.041 0.045 0.053

IAM1 0.203 0.214 0.219 0.246 0.273 0.278 0.288

CM1 0.183 0.192 0.196 0.221 0.245 0.250 0.259

WINT1 ON

AG1 -0.171 -0.164 -0.160 -0.141 -0.122 -0.119 -0.112

CX1 0.176 0.185 0.189 0.213 0.236 0.240 0.249

MAR1 -0.057 -0.050 -0.046 -0.027 -0.008 -0.004 0.003

SMOK1 -0.062 -0.053 -0.048 -0.024 0.000 0.005 0.014

DK1 0.046 0.055 0.060 0.084 0.107 0.112 0.121

TPA1 -0.045 -0.038 -0.034 -0.015 0.004 0.008 0.015

IAM1 -0.020 -0.017 -0.015 -0.005 0.004 0.006 0.010

CM1 -0.046 -0.039 -0.035 -0.014 0.006 0.010 0.018

WX1 WITH

WY1 -0.166 -0.155 -0.149 -0.119 -0.090 -0.084 -0.073

WINT1 0.091 0.099 0.104 0.127 0.149 0.154 0.162

RIX 0.000 0.000 0.000 0.000 0.000 0.000 0.000

RIY 0.000 0.000 0.000 0.000 0.000 0.000 0.000

RIINT 0.000 0.000 0.000 0.000 0.000 0.000 0.000

WY1 WITH

WINT1 -0.069 -0.060 -0.056 -0.033 -0.011 -0.006 0.002

RIX 0.000 0.000 0.000 0.000 0.000 0.000 0.000

RIY 0.000 0.000 0.000 0.000 0.000 0.000 0.000

WX2 WITH

WY2 -0.245 -0.232 -0.226 -0.192 -0.157 -0.151 -0.138

WINT2 0.081 0.090 0.094 0.117 0.140 0.144 0.153

WY2 WITH

WINT2 -0.089 -0.081 -0.077 -0.055 -0.033 -0.029 -0.021

WX3 WITH

WY3 -0.213 -0.202 -0.197 -0.169 -0.142 -0.136 -0.126

WINT3 0.060 0.069 0.073 0.097 0.121 0.125 0.134

WY3 WITH

WINT3 -0.085 -0.076 -0.072 -0.048 -0.024 -0.020 -0.011

RIINT WITH

WINT1 0.000 0.000 0.000 0.000 0.000 0.000 0.000

Intercepts

X1 2.722 2.793 2.829 3.018 3.208 3.244 3.315

X2 2.611 2.691 2.732 2.947 3.161 3.202 3.282

X3 2.633 2.714 2.755 2.972 3.189 3.230 3.311

Y1 2.075 2.146 2.183 2.372 2.562 2.598 2.669

Y2 2.322 2.395 2.433 2.628 2.823 2.861 2.934

Y3 1.840 1.915 1.953 2.152 2.351 2.389 2.464

INT1 -0.316 -0.255 -0.224 -0.060 0.103 0.134 0.195

INT2 0.223 0.292 0.328 0.513 0.699 0.735 0.804

INT3 2.582 2.645 2.677 2.846 3.015 3.047 3.110

Residual Variances

X1 0.000 0.000 0.000 0.000 0.000 0.000 0.000

X2 0.000 0.000 0.000 0.000 0.000 0.000 0.000

X3 0.000 0.000 0.000 0.000 0.000 0.000 0.000

Y1 0.000 0.000 0.000 0.000 0.000 0.000 0.000

Y2 0.000 0.000 0.000 0.000 0.000 0.000 0.000

Y3 0.000 0.000 0.000 0.000 0.000 0.000 0.000

INT1 0.000 0.000 0.000 0.000 0.000 0.000 0.000

INT2 0.000 0.000 0.000 0.000 0.000 0.000 0.000

INT3 0.000 0.000 0.000 0.000 0.000 0.000 0.000

RIX 0.624 0.635 0.640 0.666 0.693 0.698 0.708

RIINT 0.588 0.617 0.633 0.712 0.791 0.806 0.836

RIY 0.897 0.903 0.906 0.922 0.938 0.941 0.947

WX1 0.893 0.898 0.901 0.915 0.930 0.933 0.938

WX2 0.825 0.832 0.836 0.856 0.875 0.879 0.886

WX3 0.769 0.778 0.782 0.807 0.831 0.835 0.845

WINT1 0.907 0.912 0.914 0.926 0.937 0.940 0.944

WINT2 0.823 0.829 0.832 0.847 0.863 0.866 0.872

WINT3 0.699 0.705 0.707 0.721 0.735 0.738 0.743

WY1 0.830 0.837 0.840 0.858 0.876 0.879 0.886

WY2 0.790 0.798 0.802 0.824 0.846 0.850 0.858

WY3 0.741 0.750 0.755 0.780 0.804 0.809 0.818

CONFIDENCE INTERVALS OF TOTAL, TOTAL INDIRECT, SPECIFIC INDIRECT, AND DIRECT EFFECTS

Lower .5% Lower 2.5% Lower 5% Estimate Upper 5% Upper 2.5% Upper .5%

Effects from WX1 to WY3

Total -0.099 -0.093 -0.090 -0.073 -0.056 -0.052 -0.046

Total indirect -0.099 -0.093 -0.090 -0.073 -0.056 -0.052 -0.046

Specific indirect 1

WY3

WX2

WX1 -0.057 -0.053 -0.051 -0.039 -0.028 -0.026 -0.021

Specific indirect 2

WY3

WINT2

WX1 -0.013 -0.012 -0.011 -0.007 -0.003 -0.002 -0.001

Specific indirect 3

WY3

WY2

WX1 -0.040 -0.037 -0.035 -0.027 -0.018 -0.016 -0.013

Effects from WY1 to WX3

Total -0.027 -0.025 -0.024 -0.018 -0.013 -0.012 -0.010

Total indirect -0.027 -0.025 -0.024 -0.018 -0.013 -0.012 -0.010

Specific indirect 1

WX3

WX2

WY1 -0.015 -0.014 -0.013 -0.010 -0.007 -0.006 -0.005

Specific indirect 2

WX3

WINT2

WY1 -0.003 -0.003 -0.003 -0.002 -0.001 -0.001 0.000

Specific indirect 3

WX3

WY2

WY1 -0.011 -0.010 -0.010 -0.007 -0.004 -0.003 -0.002

CONFIDENCE INTERVALS OF STANDARDIZED TOTAL, TOTAL INDIRECT, SPECIFIC INDIRECT, AND DIRECT EFFECTS

STDYX Standardization

Lower .5% Lower 2.5% Lower 5% Estimate Upper 5% Upper 2.5% Upper .5%

Effects from WX1 to WY3

Total -0.048 -0.045 -0.043 -0.035 -0.027 -0.025 -0.022

Total indirect -0.048 -0.045 -0.043 -0.035 -0.027 -0.025 -0.022

Specific indirect 1

WY3

WX2

WX1 -0.028 -0.025 -0.024 -0.019 -0.013 -0.012 -0.010

Specific indirect 2

WY3

WINT2

WX1 -0.006 -0.006 -0.005 -0.003 -0.001 -0.001 0.000

Specific indirect 3

WY3

WY2

WX1 -0.019 -0.018 -0.017 -0.013 -0.009 -0.008 -0.006

Effects from WY1 to WX3

Total -0.046 -0.042 -0.040 -0.031 -0.022 -0.020 -0.017

Total indirect -0.046 -0.042 -0.040 -0.031 -0.022 -0.020 -0.017

Specific indirect 1

WX3

WX2

WY1 -0.025 -0.023 -0.022 -0.017 -0.012 -0.011 -0.009

Specific indirect 2

WX3

WINT2

WY1 -0.005 -0.005 -0.004 -0.003 -0.001 -0.001 0.000

Specific indirect 3

WX3

WY2

WY1 -0.019 -0.017 -0.016 -0.012 -0.007 -0.006 -0.004

TECHNICAL 1 OUTPUT

DIAGRAM INFORMATION

Use View Diagram under the Diagram menu in the Mplus Editor to view the diagram.

If running Mplus from the Mplus Diagrammer, the diagram opens automatically.

Diagram output

t:\mplus\shuj\xcx1.dgm

Beginning Time: 16:30:55

Ending Time: 16:39:57

Elapsed Time: 00:09:02

MUTHEN & MUTHEN

3463 Stoner Ave.

Los Angeles, CA 90066

Tel: (310) 391-9971

Fax: (310) 391-8971

Web: www.StatModel.com

Support: Support@StatModel.com

Copyright (c) 1998-2019 Muthen & Muthen

Model 5i 45-64,65+

(age45-64)subgroup

DATA:

file is RICX45.dat;

FORMAT IS FREE;

TYPE IS INDIVIDUAL;

VARIABLE:

Names = id1 x1-x3 y1-y3 cm1-cm3 sex edu cx1-cx3 mar1-mar3 smok1-smok3

iam1-iam3 ia1-ia3 dk1-dk3 soa1-soa3 int1-int3 tpa1-tpa3 ag1-ag3 ;

USEVARIABLES = x1-x3 y1-y3 cm1-cm3 sex edu cx1-cx3 mar1-mar3 smok1-smok3

iam1-iam3 dk1-dk3 int1-int3 tpa1-tpa3 ag1-ag3;

MISSING = ALL(-1);

Analysis:model= NOCOV;

COVERAGE=0.00001;

bootstrap=500

ESTIMATOR=MLM;

MODEL:

RIx BY x1@1 x2@1 x3@1;

RIint BY int1@1 int2@1 int3@1;

RIy BY y1@1 y2@1 y3@1;

wx1 BY x1@1;

wx2 BY x2@1;

wx3 BY x3@1;

wint1 BY int1@1;

wint2 BY int2@1;

wint3 BY int3@1;

wy1 BY y1@1;

wy2 BY y2@1;

wy3 BY y3@1;

x1-X3@0;

int1-int3@0;

y1-y3@0;

!控制变量 时间不变变量 on sex edu;

RIx on sex edu;

RIY on sex edu;

RIint on sex edu;

wx2 on ag2 cx2 mar2 smok2 dk2 tpa2 cm2 iam2;

wy2 on ag2 cx2 mar2 smok2 dk2 tpa2 cm2 iam2;

wint2 on ag2 cx2 mar2 smok2 dk2 tpa2 cm2 iam2;

wx3 on ag3 cx3 mar3 smok3 dk3 iam3 tpa3 cm3;

wy3 on ag3 cx3 mar3 smok3 dk3 iam3 tpa3 cm3;

wint3 on ag3 cx3 mar3 smok3 dk3 iam3 tpa3 cm3;

wx1 on ag1 cx1 mar1 smok1 dk1 tpa1 iam1 cm1;

wy1 on ag1 cx1 mar1 smok1 dk1 tpa1 iam1 cm1;

wint1 on ag1 cx1 mar1 smok1 dk1 tpa1 iam1 cm1;

wx2 on wx1(a1);

wx3 on wx2(a1);

wint2 on wint1(b1);

wint3 on wint2(b1);

wy2 on wy1(c1);

wy3 on wy2(c1);

wy2 on wx1 (d1);

wy3 on wx2 (d1);

wx2 on wy1 (e1);

wx3 on wy2 (e1);

wx2 on wint1(t1); wint2 on wx1(t2);

wx3 on wint2(t1); wint3 on wx2(t2);

wy2 on wint1(t3); wint2 on wy1(t4);

wy3 on wint2(t3); wint3 on wy2(t4);

wx1 WITH wy1;

wx1 WITH wint1;

wy1 WITH wint1;

wx2 WITH wy2;

wx2 WITH wint2;

wy2 WITH wint2;

wx3 WITH wy3;

wx3 WITH wint3;

wy3 WITH wint3;

RIx WITH wx1@0 wy1@0;

RIy WITH wx1@0 wy1@0;

RIint WITH wx1@0 wint1@0;

Model indirect: wy3 IND wx1;

wx3 IND wy1;

OUTPUT: STDYX SAMPSTAT CINTERVAL

Age 45-64 group , Reuslts:

THE MODEL ESTIMATION TERMINATED NORMALLY

MODEL FIT INFORMATION

Number of Free Parameters 117

Loglikelihood

H0 Value -107391.032

H1 Value -106723.111

Information Criteria

Akaike (AIC) 215016.064

Bayesian (BIC) 215822.580

Sample-Size Adjusted BIC 215450.780

(n* = (n + 2) / 24)

Chi-Square Test of Model Fit

Value 1335.841

Degrees of Freedom 171

P-Value 0.0000

RMSEA (Root Mean Square Error Of Approximation)

Estimate 0.031

90 Percent C.I. 0.029 0.032

Probability RMSEA <= .05 1.000

CFI/TLI

CFI 0.948

TLI 0.917

Chi-Square Test of Model Fit for the Baseline Model

Value 22492.303

Degrees of Freedom 270

P-Value 0.0000

SRMR (Standardized Root Mean Square Residual)

Value 0.029

MODEL RESULTS

Two-Tailed

Estimate S.E. Est./S.E. P-Value

RIX BY

X1 1.000 0.000 999.000 999.000

X2 1.000 0.000 999.000 999.000

X3 1.000 0.000 999.000 999.000

RIINT BY

INT1 1.000 0.000 999.000 999.000

INT2 1.000 0.000 999.000 999.000

INT3 1.000 0.000 999.000 999.000

RIY BY

Y1 1.000 0.000 999.000 999.000

Y2 1.000 0.000 999.000 999.000

Y3 1.000 0.000 999.000 999.000

WX1 BY

X1 1.000 0.000 999.000 999.000

WX2 BY

X2 1.000 0.000 999.000 999.000

WX3 BY

X3 1.000 0.000 999.000 999.000

WINT1 BY

INT1 1.000 0.000 999.000 999.000

WINT2 BY

INT2 1.000 0.000 999.000 999.000

WINT3 BY

INT3 1.000 0.000 999.000 999.000

WY1 BY

Y1 1.000 0.000 999.000 999.000

WY2 BY

Y2 1.000 0.000 999.000 999.000

WY3 BY

Y3 1.000 0.000 999.000 999.000

WX2 ON

WX1 0.305 0.020 15.221 0.000

WY1 -0.060 0.008 -7.913 0.000

WINT1 0.653 0.083 7.914 0.000

WY2 ON

WY1 0.187 0.024 7.728 0.000

WX1 -0.200 0.026 -7.578 0.000

WINT1 -0.371 0.156 -2.372 0.018

WINT2 ON

WINT1 0.293 0.018 16.501 0.000

WX1 0.019 0.001 13.038 0.000

WY1 -0.002 0.001 -2.668 0.008

WX3 ON

WX2 0.305 0.020 15.221 0.000

WY2 -0.060 0.008 -7.913 0.000

WINT2 0.653 0.083 7.914 0.000

WY3 ON

WY2 0.187 0.024 7.728 0.000

WX2 -0.200 0.026 -7.578 0.000

WINT2 -0.371 0.156 -2.372 0.018

WINT3 ON

WINT2 0.293 0.018 16.501 0.000

WX2 0.019 0.001 13.038 0.000

WY2 -0.002 0.001 -2.668 0.008

RIX ON

SEX 0.115 0.077 1.490 0.136

EDU 2.135 0.050 43.088 0.000

RIY ON

SEX -1.508 0.133 -11.322 0.000

EDU -0.849 0.086 -9.866 0.000

RIINT ON

SEX -0.011 0.008 -1.422 0.155

EDU 0.093 0.004 22.925 0.000

WX2 ON

AG2 -0.081 0.008 -10.239 0.000

CX2 0.765 0.093 8.182 0.000

MAR2 0.568 0.171 3.321 0.001

SMOK2 -0.287 0.096 -2.997 0.003

DK2 0.181 0.090 2.024 0.043

TPA2 -0.063 0.080 -0.796 0.426

CM2 0.033 0.024 1.367 0.172

IAM2 -0.301 0.035 -8.716 0.000

WY2 ON

AG2 -0.084 0.012 -6.782 0.000

CX2 -0.842 0.140 -5.993 0.000

MAR2 -1.595 0.275 -5.795 0.000

SMOK2 0.240 0.152 1.579 0.114

DK2 -0.337 0.138 -2.449 0.014

TPA2 0.277 0.114 2.438 0.015

CM2 0.502 0.044 11.476 0.000

IAM2 0.697 0.048 14.389 0.000

WINT2 ON

AG2 -0.007 0.001 -9.340 0.000

CX2 0.101 0.010 10.508 0.000

MAR2 -0.007 0.012 -0.569 0.570

SMOK2 -0.003 0.009 -0.348 0.728

DK2 0.046 0.008 5.551 0.000

TPA2 -0.010 0.006 -1.800 0.072

CM2 -0.003 0.002 -1.352 0.176

IAM2 -0.004 0.001 -4.109 0.000

WX3 ON

AG3 -0.037 0.007 -5.331 0.000

CX3 0.552 0.071 7.821 0.000

MAR3 0.185 0.130 1.422 0.155

SMOK3 -0.032 0.086 -0.368 0.713

DK3 0.233 0.072 3.214 0.001

IAM3 -0.121 0.026 -4.752 0.000

TPA3 0.073 0.061 1.207 0.227

CM3 0.030 0.018 1.621 0.105

WY3 ON

AG3 -0.031 0.013 -2.481 0.013

CX3 -0.628 0.127 -4.953 0.000

MAR3 -1.184 0.257 -4.603 0.000

SMOK3 -0.010 0.145 -0.069 0.945

DK3 -0.585 0.131 -4.465 0.000

IAM3 0.601 0.041 14.828 0.000

TPA3 0.477 0.100 4.797 0.000

CM3 0.446 0.037 12.147 0.000

WINT3 ON

AG3 -0.024 0.001 -24.583 0.000

CX3 0.123 0.011 10.992 0.000

MAR3 0.014 0.017 0.828 0.407

SMOK3 -0.001 0.012 -0.114 0.909

DK3 0.063 0.011 5.829 0.000

IAM3 -0.010 0.002 -5.335 0.000

TPA3 0.012 0.008 1.492 0.136

CM3 0.012 0.003 4.545 0.000

WX1 ON

AG1 -0.045 0.007 -6.620 0.000

CX1 0.752 0.080 9.374 0.000

MAR1 0.489 0.181 2.699 0.007

SMOK1 -0.220 0.089 -2.467 0.014

DK1 0.114 0.079 1.445 0.148

TPA1 -0.129 0.046 -2.829 0.005

IAM1 -0.173 0.036 -4.803 0.000

CM1 0.020 0.028 0.704 0.482

WY1 ON

AG1 -0.052 0.012 -4.232 0.000

CX1 -0.935 0.142 -6.586 0.000

MAR1 -2.199 0.316 -6.969 0.000

SMOK1 0.569 0.147 3.862 0.000

DK1 -0.292 0.134 -2.179 0.029

TPA1 0.120 0.074 1.621 0.105

IAM1 0.821 0.053 15.422 0.000

CM1 0.772 0.056 13.907 0.000

WINT1 ON

AG1 -0.004 0.001 -7.324 0.000

CX1 0.122 0.009 13.321 0.000

MAR1 -0.033 0.012 -2.663 0.008

SMOK1 -0.020 0.008 -2.398 0.016

DK1 0.044 0.008 5.783 0.000

TPA1 0.000 0.003 -0.137 0.891

IAM1 0.000 0.001 -0.245 0.807

CM1 -0.004 0.002 -1.894 0.058

WX1 WITH

WY1 -1.399 0.205 -6.828 0.000

WINT1 0.078 0.008 9.745 0.000

RIX 0.000 0.000 999.000 999.000

RIY 0.000 0.000 999.000 999.000

RIINT 0.000 0.000 999.000 999.000

WY1 WITH

WINT1 -0.039 0.013 -2.968 0.003

RIX 0.000 0.000 999.000 999.000

RIY 0.000 0.000 999.000 999.000

WX2 WITH

WY2 -2.575 0.276 -9.341 0.000

WINT2 0.092 0.011 8.248 0.000

WY2 WITH

WINT2 -0.061 0.018 -3.455 0.001

WX3 WITH

WY3 -1.830 0.181 -10.105 0.000

WINT3 0.114 0.014 7.894 0.000

WY3 WITH

WINT3 -0.083 0.024 -3.404 0.001

RIINT WITH

WINT1 0.000 0.000 999.000 999.000

Intercepts

X1 8.376 0.448 18.678 0.000

X2 10.843 0.568 19.087 0.000

X3 9.296 0.489 19.013 0.000

Y1 14.349 0.809 17.740 0.000

Y2 17.021 0.887 19.188 0.000

Y3 13.214 0.895 14.772 0.000

INT1 -0.016 0.038 -0.431 0.667

INT2 0.258 0.051 5.096 0.000

INT3 1.570 0.071 22.135 0.000

Residual Variances

X1 0.000 0.000 999.000 999.000

X2 0.000 0.000 999.000 999.000

X3 0.000 0.000 999.000 999.000

Y1 0.000 0.000 999.000 999.000

Y2 0.000 0.000 999.000 999.000

Y3 0.000 0.000 999.000 999.000

INT1 0.000 0.000 999.000 999.000

INT2 0.000 0.000 999.000 999.000

INT3 0.000 0.000 999.000 999.000

RIX 2.752 0.167 16.507 0.000

RIINT 0.007 0.002 4.661 0.000

RIY 10.229 0.547 18.693 0.000

WX1 4.915 0.160 30.707 0.000

WX2 7.742 0.198 39.199 0.000

WX3 5.907 0.149 39.718 0.000

WINT1 0.058 0.002 23.996 0.000

WINT2 0.093 0.002 39.185 0.000

WINT3 0.174 0.002 85.694 0.000

WY1 18.795 0.604 31.131 0.000

WY2 21.903 0.667 32.822 0.000

WY3 18.900 0.502 37.639 0.000

STANDARDIZED MODEL RESULTS

STDYX Standardization

Two-Tailed

Estimate S.E. Est./S.E. P-Value

RIX BY

X1 0.686 0.012 57.842 0.000

X2 0.568 0.011 50.236 0.000

X3 0.609 0.012 49.794 0.000

RIINT BY

INT1 0.387 0.026 14.621 0.000

INT2 0.302 0.022 14.059 0.000

INT3 0.217 0.015 14.274 0.000

RIY BY

Y1 0.572 0.014 42.096 0.000

Y2 0.532 0.014 37.558 0.000

Y3 0.547 0.014 39.455 0.000

WX1 BY

X1 0.689 0.011 61.910 0.000

WX2 BY

X2 0.774 0.008 92.091 0.000

WX3 BY

X3 0.745 0.010 77.020 0.000

WINT1 BY

INT1 0.902 0.011 82.901 0.000

WINT2 BY

INT2 0.933 0.007 135.700 0.000

WINT3 BY

INT3 0.957 0.004 265.248 0.000

WY1 BY

Y1 0.806 0.010 84.214 0.000

WY2 BY

Y2 0.830 0.009 89.623 0.000

WY3 BY

Y3 0.816 0.009 86.835 0.000

WX2 ON

WX1 0.225 0.015 14.702 0.000

WY1 -0.093 0.012 -7.780 0.000

WINT1 0.053 0.007 7.499 0.000

WY2 ON

WY1 0.169 0.021 7.888 0.000

WX1 -0.087 0.012 -7.284 0.000

WINT1 -0.018 0.007 -2.359 0.018

WINT2 ON

WINT1 0.222 0.015 15.138 0.000

WX1 0.133 0.010 13.453 0.000

WY1 -0.029 0.011 -2.676 0.007

WX3 ON

WX2 0.340 0.022 15.399 0.000

WY2 -0.114 0.014 -7.863 0.000

WINT2 0.077 0.010 7.697 0.000

WY3 ON

WY2 0.195 0.026 7.602 0.000

WX2 -0.123 0.017 -7.411 0.000

WINT2 -0.024 0.010 -2.368 0.018

WINT3 ON

WINT2 0.205 0.013 15.752 0.000

WX2 0.126 0.010 12.836 0.000

WY2 -0.022 0.008 -2.676 0.007

RIX ON

SEX 0.025 0.017 1.494 0.135

EDU 0.679 0.014 49.755 0.000

RIY ON

SEX -0.223 0.019 -11.503 0.000

EDU -0.182 0.018 -9.962 0.000

RIINT ON

SEX -0.051 0.037 -1.383 0.167

EDU 0.627 0.048 13.027 0.000

WX2 ON

AG2 -0.141 0.014 -10.278 0.000

CX2 0.108 0.013 8.242 0.000

MAR2 0.049 0.015 3.306 0.001

SMOK2 -0.042 0.014 -2.988 0.003

DK2 0.028 0.014 2.023 0.043

TPA2 -0.012 0.015 -0.797 0.426

CM2 0.019 0.014 1.365 0.172

IAM2 -0.194 0.022 -8.731 0.000

WY2 ON

AG2 -0.086 0.013 -6.775 0.000

CX2 -0.070 0.012 -5.976 0.000

MAR2 -0.081 0.014 -5.686 0.000

SMOK2 0.021 0.013 1.580 0.114

DK2 -0.031 0.013 -2.451 0.014

TPA2 0.031 0.013 2.445 0.014

CM2 0.170 0.015 11.075 0.000

IAM2 0.264 0.019 14.034 0.000

WINT2 ON

AG2 -0.107 0.011 -9.551 0.000

CX2 0.134 0.012 10.811 0.000

MAR2 -0.006 0.010 -0.569 0.569

SMOK2 -0.005 0.013 -0.348 0.728

DK2 0.066 0.012 5.582 0.000

TPA2 -0.019 0.010 -1.803 0.071

CM2 -0.016 0.012 -1.354 0.176

IAM2 -0.026 0.006 -4.157 0.000

WX3 ON

AG3 -0.072 0.014 -5.265 0.000

CX3 0.094 0.012 7.870 0.000

MAR3 0.019 0.013 1.428 0.153

SMOK3 -0.005 0.014 -0.367 0.713

DK3 0.041 0.013 3.207 0.001

IAM3 -0.092 0.019 -4.714 0.000

TPA3 0.016 0.013 1.211 0.226

CM3 0.021 0.013 1.613 0.107

WY3 ON

AG3 -0.034 0.014 -2.471 0.013

CX3 -0.059 0.012 -4.932 0.000

MAR3 -0.067 0.015 -4.585 0.000

SMOK3 -0.001 0.013 -0.069 0.945

DK3 -0.057 0.013 -4.438 0.000

IAM3 0.252 0.017 14.785 0.000

TPA3 0.059 0.012 4.800 0.000

CM3 0.177 0.015 11.875 0.000

WINT3 ON

AG3 -0.275 0.011 -24.467 0.000

CX3 0.123 0.011 10.906 0.000

MAR3 0.009 0.010 0.830 0.407

SMOK3 -0.001 0.011 -0.114 0.909

DK3 0.065 0.011 5.824 0.000

IAM3 -0.045 0.009 -5.213 0.000

TPA3 0.016 0.011 1.491 0.136

CM3 0.050 0.011 4.526 0.000

WX1 ON

AG1 -0.106 0.016 -6.622 0.000

CX1 0.146 0.015 9.548 0.000

MAR1 0.051 0.019 2.722 0.006

SMOK1 -0.044 0.018 -2.471 0.013

DK1 0.024 0.017 1.441 0.150

TPA1 -0.045 0.016 -2.827 0.005

IAM1 -0.127 0.026 -4.908 0.000

CM1 0.012 0.017 0.704 0.481

WY1 ON

AG1 -0.059 0.014 -4.254 0.000

CX1 -0.087 0.013 -6.701 0.000

MAR1 -0.110 0.016 -6.994 0.000

SMOK1 0.055 0.014 3.863 0.000

DK1 -0.030 0.014 -2.174 0.030

TPA1 0.020 0.012 1.619 0.105

IAM1 0.290 0.017 16.586 0.000

CM1 0.218 0.015 14.152 0.000

WINT1 ON

AG1 -0.093 0.012 -7.631 0.000

CX1 0.216 0.014 14.930 0.000

MAR1 -0.031 0.012 -2.680 0.007

SMOK1 -0.036 0.015 -2.390 0.017

DK1 0.085 0.015 5.868 0.000

TPA1 -0.001 0.011 -0.137 0.891

IAM1 -0.001 0.006 -0.246 0.806

CM1 -0.023 0.012 -1.908 0.056

WX1 WITH

WY1 -0.146 0.020 -7.203 0.000

WINT1 0.146 0.014 10.411 0.000

RIX 0.000 0.000 0.000 1.000

RIY 0.000 0.000 0.000 1.000

RIINT 0.000 0.000 0.000 1.000

WY1 WITH

WINT1 -0.037 0.013 -2.987 0.003

RIX 0.000 0.000 0.000 1.000

RIY 0.000 0.000 0.000 1.000

WX2 WITH

WY2 -0.198 0.022 -9.155 0.000

WINT2 0.109 0.013 8.188 0.000

WY2 WITH

WINT2 -0.042 0.012 -3.456 0.001

WX3 WITH

WY3 -0.173 0.017 -10.326 0.000

WINT3 0.112 0.014 7.927 0.000

WY3 WITH

WINT3 -0.046 0.013 -3.398 0.001

RIINT WITH

WINT1 0.000 0.000 0.000 1.000

Intercepts

X1 2.518 0.141 17.873 0.000

X2 2.701 0.144 18.735 0.000

X3 2.481 0.133 18.655 0.000

Y1 2.425 0.134 18.159 0.000

Y2 2.678 0.138 19.382 0.000

Y3 2.137 0.144 14.869 0.000

INT1 -0.059 0.138 -0.431 0.667

INT2 0.727 0.141 5.143 0.000

INT3 3.174 0.143 22.255 0.000

Residual Variances

X1 0.000 0.000 0.000 1.000

X2 0.000 0.000 0.000 1.000

X3 0.000 0.000 0.000 1.000

Y1 0.000 0.000 0.000 1.000

Y2 0.000 0.000 0.000 1.000

Y3 0.000 0.000 0.000 1.000

INT1 0.000 0.000 0.000 1.000

INT2 0.000 0.000 0.000 1.000

INT3 0.000 0.000 0.000 1.000

RIX 0.529 0.018 29.458 0.000

RIINT 0.624 0.059 10.606 0.000

RIY 0.893 0.012 75.696 0.000

WX1 0.936 0.010 90.993 0.000

WX2 0.802 0.013 61.477 0.000

WX3 0.758 0.015 52.097 0.000

WINT1 0.932 0.007 129.813 0.000

WINT2 0.853 0.009 94.049 0.000

WINT3 0.778 0.009 89.098 0.000

WY1 0.825 0.013 64.865 0.000

WY2 0.786 0.013 58.592 0.000

WY3 0.742 0.014 51.667 0.000

R-SQUARE

Observed Two-Tailed

Variable Estimate S.E. Est./S.E. P-Value

X1 1.000 0.000 999.000 999.000

X2 1.000 0.000 999.000 999.000

X3 1.000 0.000 999.000 999.000

Y1 1.000 0.000 999.000 999.000

Y2 1.000 0.000 999.000 999.000

Y3 1.000 0.000 999.000 999.000

INT1 1.000 0.000 999.000 999.000

INT2 1.000 0.000 999.000 999.000

INT3 1.000 0.000 999.000 999.000

Latent Two-Tailed

Variable Estimate S.E. Est./S.E. P-Value

RIX 0.471 0.018 26.248 0.000

RIINT 0.376 0.059 6.401 0.000

RIY 0.107 0.012 9.036 0.000

WX1 0.064 0.010 6.235 0.000

WX2 0.198 0.013 15.147 0.000

WX3 0.242 0.015 16.599 0.000

WINT1 0.068 0.007 9.413 0.000

WINT2 0.147 0.009 16.236 0.000

WINT3 0.222 0.009 25.412 0.000

WY1 0.175 0.013 13.713 0.000

WY2 0.214 0.013 15.913 0.000

WY3 0.258 0.014 17.979 0.000

TOTAL, TOTAL INDIRECT, SPECIFIC INDIRECT, AND DIRECT EFFECTS

Two-Tailed

Estimate S.E. Est./S.E. P-Value

Effects from WX1 to WY3

Total -0.105 0.012 -8.505 0.000

Total indirect -0.105 0.012 -8.505 0.000

Specific indirect 1

WY3

WX2

WX1 -0.061 0.009 -6.760 0.000

Specific indirect 2

WY3

WINT2

WX1 -0.007 0.003 -2.368 0.018

Specific indirect 3

WY3

WY2

WX1 -0.037 0.005 -6.886 0.000

Effects from WY1 to WX3

Total -0.031 0.004 -8.286 0.000

Total indirect -0.031 0.004 -8.286 0.000

Specific indirect 1

WX3

WX2

WY1 -0.018 0.002 -8.079 0.000

Specific indirect 2

WX3

WINT2

WY1 -0.001 0.001 -2.607 0.009

Specific indirect 3

WX3

WY2

WY1 -0.011 0.002 -5.786 0.000

STANDARDIZED TOTAL, TOTAL INDIRECT, SPECIFIC INDIRECT, AND DIRECT EFFECTS

STDYX Standardization

Two-Tailed

Estimate S.E. Est./S.E. P-Value

Effects from WX1 to WY3

Total -0.048 0.006 -8.298 0.000

Total indirect -0.048 0.006 -8.298 0.000

Specific indirect 1

WY3

WX2

WX1 -0.028 0.004 -6.460 0.000

Specific indirect 2

WY3

WINT2

WX1 -0.003 0.001 -2.360 0.018

Specific indirect 3

WY3

WY2

WX1 -0.017 0.002 -7.114 0.000

Effects from WY1 to WX3

Total -0.053 0.006 -8.161 0.000

Total indirect -0.053 0.006 -8.161 0.000

Specific indirect 1

WX3

WX2

WY1 -0.031 0.004 -8.295 0.000

Specific indirect 2

WX3

WINT2

WY1 -0.002 0.001 -2.609 0.009

Specific indirect 3

WX3

WY2

WY1 -0.019 0.003 -5.559 0.000

CONFIDENCE INTERVALS OF MODEL RESULTS

Lower .5% Lower 2.5% Lower 5% Estimate Upper 5% Upper 2.5% Upper .5%

RIX BY

X1 1.000 1.000 1.000 1.000 1.000 1.000 1.000

X2 1.000 1.000 1.000 1.000 1.000 1.000 1.000

X3 1.000 1.000 1.000 1.000 1.000 1.000 1.000

RIINT BY

INT1 1.000 1.000 1.000 1.000 1.000 1.000 1.000

INT2 1.000 1.000 1.000 1.000 1.000 1.000 1.000

INT3 1.000 1.000 1.000 1.000 1.000 1.000 1.000

RIY BY

Y1 1.000 1.000 1.000 1.000 1.000 1.000 1.000

Y2 1.000 1.000 1.000 1.000 1.000 1.000 1.000

Y3 1.000 1.000 1.000 1.000 1.000 1.000 1.000

WX1 BY

X1 1.000 1.000 1.000 1.000 1.000 1.000 1.000

WX2 BY

X2 1.000 1.000 1.000 1.000 1.000 1.000 1.000

WX3 BY

X3 1.000 1.000 1.000 1.000 1.000 1.000 1.000

WINT1 BY

INT1 1.000 1.000 1.000 1.000 1.000 1.000 1.000

WINT2 BY

INT2 1.000 1.000 1.000 1.000 1.000 1.000 1.000

WINT3 BY

INT3 1.000 1.000 1.000 1.000 1.000 1.000 1.000

WY1 BY

Y1 1.000 1.000 1.000 1.000 1.000 1.000 1.000

WY2 BY

Y2 1.000 1.000 1.000 1.000 1.000 1.000 1.000

WY3 BY

Y3 1.000 1.000 1.000 1.000 1.000 1.000 1.000

WX2 ON

WX1 0.253 0.266 0.272 0.305 0.338 0.344 0.357

WY1 -0.080 -0.075 -0.073 -0.060 -0.048 -0.045 -0.041

WINT1 0.440 0.491 0.517 0.653 0.789 0.815 0.866

WY2 ON

WY1 0.124 0.139 0.147 0.187 0.226 0.234 0.249

WX1 -0.268 -0.252 -0.243 -0.200 -0.156 -0.148 -0.132

WINT1 -0.774 -0.678 -0.628 -0.371 -0.114 -0.064 0.032

WINT2 ON

WINT1 0.248 0.258 0.264 0.293 0.323 0.328 0.339

WX1 0.015 0.016 0.017 0.019 0.022 0.022 0.023

WY1 -0.004 -0.003 -0.003 -0.002 -0.001 -0.001 0.000

WX3 ON

WX2 0.253 0.266 0.272 0.305 0.338 0.344 0.357

WY2 -0.080 -0.075 -0.073 -0.060 -0.048 -0.045 -0.041

WINT2 0.440 0.491 0.517 0.653 0.789 0.815 0.866

WY3 ON

WY2 0.124 0.139 0.147 0.187 0.226 0.234 0.249

WX2 -0.268 -0.252 -0.243 -0.200 -0.156 -0.148 -0.132

WINT2 -0.774 -0.678 -0.628 -0.371 -0.114 -0.064 0.032

WINT3 ON

WINT2 0.248 0.258 0.264 0.293 0.323 0.328 0.339

WX2 0.015 0.016 0.017 0.019 0.022 0.022 0.023

WY2 -0.004 -0.003 -0.003 -0.002 -0.001 -0.001 0.000

RIX ON

SEX -0.084 -0.036 -0.012 0.115 0.242 0.266 0.313

EDU 2.008 2.038 2.054 2.135 2.217 2.232 2.263

RIY ON

SEX -1.851 -1.769 -1.727 -1.508 -1.289 -1.247 -1.165

EDU -1.071 -1.018 -0.991 -0.849 -0.708 -0.681 -0.628

RIINT ON

SEX -0.031 -0.026 -0.024 -0.011 0.002 0.004 0.009

EDU 0.082 0.085 0.086 0.093 0.099 0.101 0.103

WX2 ON

AG2 -0.101 -0.096 -0.094 -0.081 -0.068 -0.065 -0.060

CX2 0.524 0.581 0.611 0.765 0.918 0.948 1.005

MAR2 0.127 0.233 0.287 0.568 0.849 0.903 1.009

SMOK2 -0.534 -0.475 -0.445 -0.287 -0.130 -0.099 -0.040

DK2 -0.049 0.006 0.034 0.181 0.329 0.357 0.412

TPA2 -0.269 -0.220 -0.195 -0.063 0.068 0.093 0.142

CM2 -0.029 -0.014 -0.007 0.033 0.073 0.081 0.095

IAM2 -0.390 -0.368 -0.358 -0.301 -0.244 -0.233 -0.212

WY2 ON

AG2 -0.116 -0.108 -0.104 -0.084 -0.063 -0.060 -0.052

CX2 -1.203 -1.117 -1.073 -0.842 -0.611 -0.566 -0.480

MAR2 -2.304 -2.134 -2.048 -1.595 -1.142 -1.055 -0.886

SMOK2 -0.151 -0.058 -0.010 0.240 0.489 0.537 0.631

DK2 -0.691 -0.607 -0.563 -0.337 -0.111 -0.067 0.017

TPA2 -0.016 0.054 0.090 0.277 0.464 0.499 0.569

CM2 0.389 0.416 0.430 0.502 0.574 0.588 0.615

IAM2 0.572 0.602 0.617 0.697 0.776 0.792 0.822

WINT2 ON

AG2 -0.008 -0.008 -0.008 -0.007 -0.005 -0.005 -0.005

CX2 0.076 0.082 0.085 0.101 0.117 0.120 0.126

MAR2 -0.038 -0.031 -0.027 -0.007 0.013 0.017 0.024

SMOK2 -0.028 -0.022 -0.019 -0.003 0.012 0.015 0.021

DK2 0.024 0.029 0.032 0.046 0.059 0.062 0.067

TPA2 -0.025 -0.022 -0.020 -0.010 -0.001 0.001 0.005

CM2 -0.009 -0.007 -0.007 -0.003 0.001 0.001 0.003

IAM2 -0.007 -0.006 -0.006 -0.004 -0.003 -0.002 -0.002

WX3 ON

AG3 -0.055 -0.050 -0.048 -0.037 -0.025 -0.023 -0.019

CX3 0.370 0.414 0.436 0.552 0.669 0.691 0.734

MAR3 -0.150 -0.070 -0.029 0.185 0.400 0.441 0.521

SMOK3 -0.252 -0.199 -0.172 -0.032 0.109 0.136 0.189

DK3 0.046 0.091 0.114 0.233 0.352 0.374 0.419

IAM3 -0.187 -0.171 -0.163 -0.121 -0.079 -0.071 -0.055

TPA3 -0.083 -0.046 -0.027 0.073 0.173 0.192 0.229

CM3 -0.017 -0.006 0.000 0.030 0.060 0.065 0.077

WY3 ON

AG3 -0.064 -0.056 -0.052 -0.031 -0.011 -0.007 0.001

CX3 -0.955 -0.877 -0.837 -0.628 -0.420 -0.380 -0.302

MAR3 -1.847 -1.688 -1.607 -1.184 -0.761 -0.680 -0.521

SMOK3 -0.382 -0.293 -0.248 -0.010 0.228 0.273 0.362

DK3 -0.923 -0.842 -0.801 -0.585 -0.370 -0.328 -0.248

IAM3 0.496 0.521 0.534 0.601 0.667 0.680 0.705

TPA3 0.221 0.282 0.314 0.477 0.641 0.672 0.734

CM3 0.352 0.374 0.386 0.446 0.507 0.518 0.541

WINT3 ON

AG3 -0.026 -0.026 -0.025 -0.024 -0.022 -0.022 -0.021

CX3 0.094 0.101 0.105 0.123 0.141 0.145 0.152

MAR3 -0.030 -0.020 -0.014 0.014 0.043 0.048 0.059

SMOK3 -0.032 -0.025 -0.021 -0.001 0.018 0.022 0.029

DK3 0.035 0.042 0.045 0.063 0.081 0.085 0.091

IAM3 -0.015 -0.014 -0.013 -0.010 -0.007 -0.006 -0.005

TPA3 -0.009 -0.004 -0.001 0.012 0.026 0.029 0.034

CM3 0.005 0.007 0.008 0.012 0.016 0.017 0.019

WX1 ON

AG1 -0.062 -0.058 -0.056 -0.045 -0.034 -0.032 -0.027

CX1 0.546 0.595 0.620 0.752 0.884 0.910 0.959

MAR1 0.022 0.134 0.191 0.489 0.787 0.844 0.955

SMOK1 -0.450 -0.395 -0.367 -0.220 -0.073 -0.045 0.010

DK1 -0.089 -0.041 -0.016 0.114 0.244 0.269 0.318

TPA1 -0.247 -0.219 -0.205 -0.129 -0.054 -0.040 -0.012

IAM1 -0.266 -0.244 -0.233 -0.173 -0.114 -0.103 -0.080

CM1 -0.053 -0.036 -0.027 0.020 0.066 0.075 0.093

WY1 ON

AG1 -0.083 -0.076 -0.072 -0.052 -0.032 -0.028 -0.020

CX1 -1.301 -1.213 -1.168 -0.935 -0.701 -0.657 -0.569

MAR1 -3.012 -2.817 -2.718 -2.199 -1.680 -1.581 -1.386

SMOK1 0.190 0.280 0.327 0.569 0.812 0.858 0.949

DK1 -0.637 -0.554 -0.512 -0.292 -0.072 -0.029 0.053

TPA1 -0.071 -0.025 -0.002 0.120 0.242 0.265 0.311

IAM1 0.684 0.717 0.734 0.821 0.909 0.926 0.959

CM1 0.629 0.663 0.681 0.772 0.863 0.881 0.915

WINT1 ON

AG1 -0.006 -0.005 -0.005 -0.004 -0.003 -0.003 -0.003

CX1 0.098 0.104 0.107 0.122 0.137 0.139 0.145

MAR1 -0.065 -0.057 -0.053 -0.033 -0.013 -0.009 -0.001

SMOK1 -0.041 -0.036 -0.034 -0.020 -0.006 -0.004 0.001

DK1 0.024 0.029 0.031 0.044 0.056 0.059 0.063

TPA1 -0.009 -0.007 -0.006 0.000 0.005 0.006 0.008

IAM1 -0.003 -0.002 -0.002 0.000 0.001 0.002 0.002

CM1 -0.010 -0.009 -0.008 -0.004 -0.001 0.000 0.002

WX1 WITH

WY1 -1.927 -1.801 -1.736 -1.399 -1.062 -0.997 -0.871

WINT1 0.057 0.062 0.065 0.078 0.091 0.094 0.099

RIX 0.000 0.000 0.000 0.000 0.000 0.000 0.000

RIY 0.000 0.000 0.000 0.000 0.000 0.000 0.000

RIINT 0.000 0.000 0.000 0.000 0.000 0.000 0.000

WY1 WITH

WINT1 -0.073 -0.065 -0.061 -0.039 -0.017 -0.013 -0.005

RIX 0.000 0.000 0.000 0.000 0.000 0.000 0.000

RIY 0.000 0.000 0.000 0.000 0.000 0.000 0.000

WX2 WITH

WY2 -3.286 -3.116 -3.029 -2.575 -2.122 -2.035 -1.865

WINT2 0.064 0.070 0.074 0.092 0.111 0.114 0.121

WY2 WITH

WINT2 -0.106 -0.095 -0.090 -0.061 -0.032 -0.026 -0.015

WX3 WITH

WY3 -2.297 -2.185 -2.128 -1.830 -1.532 -1.475 -1.364

WINT3 0.077 0.085 0.090 0.114 0.137 0.142 0.151

WY3 WITH

WINT3 -0.145 -0.130 -0.123 -0.083 -0.043 -0.035 -0.020

RIINT WITH

WINT1 0.000 0.000 0.000 0.000 0.000 0.000 0.000

Intercepts

X1 7.221 7.497 7.638 8.376 9.114 9.255 9.531

X2 9.379 9.729 9.908 10.843 11.777 11.956 12.306

X3 8.037 8.338 8.492 9.296 10.101 10.255 10.556

Y1 12.266 12.764 13.019 14.349 15.680 15.935 16.433

Y2 14.736 15.282 15.561 17.021 18.480 18.759 19.305

Y3 10.910 11.461 11.743 13.214 14.686 14.968 15.518

INT1 -0.115 -0.091 -0.079 -0.016 0.046 0.058 0.082

INT2 0.127 0.159 0.175 0.258 0.341 0.357 0.388

INT3 1.387 1.431 1.453 1.570 1.686 1.708 1.752

Residual Variances

X1 0.000 0.000 0.000 0.000 0.000 0.000 0.000

X2 0.000 0.000 0.000 0.000 0.000 0.000 0.000

X3 0.000 0.000 0.000 0.000 0.000 0.000 0.000

Y1 0.000 0.000 0.000 0.000 0.000 0.000 0.000

Y2 0.000 0.000 0.000 0.000 0.000 0.000 0.000

Y3 0.000 0.000 0.000 0.000 0.000 0.000 0.000

INT1 0.000 0.000 0.000 0.000 0.000 0.000 0.000

INT2 0.000 0.000 0.000 0.000 0.000 0.000 0.000

INT3 0.000 0.000 0.000 0.000 0.000 0.000 0.000

RIX 2.322 2.425 2.477 2.752 3.026 3.078 3.181

RIINT 0.003 0.004 0.005 0.007 0.010 0.010 0.011

RIY 8.819 9.156 9.328 10.229 11.129 11.301 11.638

WX1 4.503 4.602 4.652 4.915 5.179 5.229 5.328

WX2 7.233 7.355 7.417 7.742 8.067 8.129 8.251

WX3 5.524 5.615 5.662 5.907 6.151 6.198 6.290

WINT1 0.052 0.054 0.054 0.058 0.062 0.063 0.065

WINT2 0.087 0.089 0.089 0.093 0.097 0.098 0.100

WINT3 0.169 0.170 0.171 0.174 0.178 0.178 0.180

WY1 17.240 17.612 17.802 18.795 19.788 19.978 20.350

WY2 20.184 20.595 20.805 21.903 23.001 23.211 23.622

WY3 17.607 17.916 18.074 18.900 19.726 19.884 20.194

CONFIDENCE INTERVALS OF STANDARDIZED MODEL RESULTS

STDYX Standardization

Lower .5% Lower 2.5% Lower 5% Estimate Upper 5% Upper 2.5% Upper .5%

RIX BY

X1 0.655 0.662 0.666 0.686 0.705 0.709 0.716

X2 0.539 0.546 0.550 0.568 0.587 0.591 0.597

X3 0.577 0.585 0.589 0.609 0.629 0.633 0.640

RIINT BY

INT1 0.319 0.335 0.343 0.387 0.430 0.439 0.455

INT2 0.247 0.260 0.267 0.302 0.338 0.344 0.358

INT3 0.178 0.187 0.192 0.217 0.242 0.247 0.256

RIY BY

Y1 0.537 0.545 0.549 0.572 0.594 0.598 0.607

Y2 0.496 0.505 0.509 0.532 0.556 0.560 0.569

Y3 0.512 0.520 0.525 0.547 0.570 0.575 0.583

WX1 BY

X1 0.660 0.667 0.671 0.689 0.707 0.711 0.717

WX2 BY

X2 0.752 0.757 0.760 0.774 0.788 0.790 0.796

WX3 BY

X3 0.720 0.726 0.729 0.745 0.761 0.764 0.770

WINT1 BY

INT1 0.874 0.881 0.884 0.902 0.920 0.924 0.930

WINT2 BY

INT2 0.916 0.920 0.922 0.933 0.945 0.947 0.951

WINT3 BY

INT3 0.948 0.950 0.952 0.957 0.963 0.965 0.967

WY1 BY

Y1 0.782 0.788 0.791 0.806 0.822 0.825 0.831

WY2 BY

Y2 0.807 0.812 0.815 0.830 0.846 0.849 0.854

WY3 BY

Y3 0.792 0.798 0.801 0.816 0.832 0.835 0.841

WX2 ON

WX1 0.186 0.195 0.200 0.225 0.250 0.255 0.264

WY1 -0.123 -0.116 -0.112 -0.093 -0.073 -0.069 -0.062

WINT1 0.035 0.039 0.041 0.053 0.064 0.066 0.071

WY2 ON

WY1 0.114 0.127 0.134 0.169 0.204 0.211 0.224

WX1 -0.117 -0.110 -0.106 -0.087 -0.067 -0.063 -0.056

WINT1 -0.037 -0.032 -0.030 -0.018 -0.005 -0.003 0.002

WINT2 ON

WINT1 0.184 0.193 0.198 0.222 0.246 0.250 0.259

WX1 0.107 0.113 0.117 0.133 0.149 0.152 0.158

WY1 -0.057 -0.050 -0.047 -0.029 -0.011 -0.008 -0.001

WX3 ON

WX2 0.283 0.296 0.303 0.340 0.376 0.383 0.396

WY2 -0.151 -0.142 -0.138 -0.114 -0.090 -0.086 -0.077

WINT2 0.052 0.058 0.061 0.077 0.094 0.097 0.103

WY3 ON

WY2 0.129 0.145 0.153 0.195 0.237 0.245 0.261

WX2 -0.166 -0.155 -0.150 -0.123 -0.096 -0.090 -0.080

WINT2 -0.051 -0.044 -0.041 -0.024 -0.007 -0.004 0.002

WINT3 ON

WINT2 0.172 0.180 0.184 0.205 0.226 0.231 0.239

WX2 0.101 0.107 0.110 0.126 0.142 0.145 0.151

WY2 -0.044 -0.039 -0.036 -0.022 -0.009 -0.006 -0.001

RIX ON

SEX -0.018 -0.008 -0.003 0.025 0.053 0.058 0.069

EDU 0.643 0.652 0.656 0.679 0.701 0.705 0.714

RIY ON

SEX -0.273 -0.261 -0.255 -0.223 -0.191 -0.185 -0.173

EDU -0.229 -0.218 -0.212 -0.182 -0.152 -0.146 -0.135

RIINT ON

SEX -0.146 -0.123 -0.112 -0.051 0.010 0.021 0.044

EDU 0.503 0.532 0.547 0.627 0.706 0.721 0.750

WX2 ON

AG2 -0.177 -0.168 -0.164 -0.141 -0.119 -0.114 -0.106

CX2 0.074 0.082 0.086 0.108 0.129 0.133 0.142

MAR2 0.011 0.020 0.025 0.049 0.074 0.078 0.087

SMOK2 -0.078 -0.070 -0.065 -0.042 -0.019 -0.014 -0.006

DK2 -0.008 0.001 0.005 0.028 0.051 0.055 0.064

TPA2 -0.051 -0.042 -0.037 -0.012 0.013 0.018 0.027

CM2 -0.017 -0.008 -0.004 0.019 0.042 0.047 0.055

IAM2 -0.251 -0.237 -0.230 -0.194 -0.157 -0.150 -0.136

WY2 ON

AG2 -0.119 -0.111 -0.107 -0.086 -0.065 -0.061 -0.054

CX2 -0.100 -0.093 -0.089 -0.070 -0.051 -0.047 -0.040

MAR2 -0.118 -0.109 -0.105 -0.081 -0.058 -0.053 -0.044

SMOK2 -0.013 -0.005 -0.001 0.021 0.042 0.046 0.054

DK2 -0.063 -0.055 -0.052 -0.031 -0.010 -0.006 0.002

TPA2 -0.002 0.006 0.010 0.031 0.052 0.056 0.063

CM2 0.131 0.140 0.145 0.170 0.196 0.201 0.210

IAM2 0.215 0.227 0.233 0.264 0.295 0.301 0.312

WINT2 ON

AG2 -0.136 -0.129 -0.126 -0.107 -0.089 -0.085 -0.078

CX2 0.102 0.109 0.113 0.134 0.154 0.158 0.166

MAR2 -0.031 -0.025 -0.022 -0.006 0.011 0.014 0.020

SMOK2 -0.038 -0.030 -0.026 -0.005 0.017 0.021 0.029

DK2 0.036 0.043 0.047 0.066 0.086 0.090 0.097

TPA2 -0.045 -0.039 -0.035 -0.019 -0.002 0.002 0.008

CM2 -0.047 -0.039 -0.036 -0.016 0.003 0.007 0.015

IAM2 -0.042 -0.038 -0.036 -0.026 -0.016 -0.014 -0.010

WX3 ON

AG3 -0.108 -0.099 -0.095 -0.072 -0.050 -0.045 -0.037

CX3 0.063 0.070 0.074 0.094 0.113 0.117 0.124

MAR3 -0.015 -0.007 -0.003 0.019 0.041 0.045 0.054

SMOK3 -0.041 -0.032 -0.028 -0.005 0.018 0.022 0.030

DK3 0.008 0.016 0.020 0.041 0.061 0.065 0.073

IAM3 -0.142 -0.130 -0.124 -0.092 -0.060 -0.054 -0.042

TPA3 -0.018 -0.010 -0.006 0.016 0.039 0.043 0.051

CM3 -0.013 -0.005 0.000 0.021 0.043 0.047 0.055

WY3 ON

AG3 -0.070 -0.061 -0.057 -0.034 -0.011 -0.007 0.001

CX3 -0.090 -0.082 -0.078 -0.059 -0.039 -0.035 -0.028

MAR3 -0.105 -0.096 -0.092 -0.067 -0.043 -0.039 -0.030

SMOK3 -0.034 -0.026 -0.022 -0.001 0.020 0.024 0.032

DK3 -0.089 -0.082 -0.077 -0.057 -0.036 -0.032 -0.024

IAM3 0.208 0.218 0.224 0.252 0.280 0.285 0.296

TPA3 0.027 0.035 0.039 0.059 0.079 0.083 0.091

CM3 0.138 0.147 0.152 0.177 0.201 0.206 0.215

WINT3 ON

AG3 -0.305 -0.298 -0.294 -0.275 -0.257 -0.253 -0.246

CX3 0.094 0.101 0.104 0.123 0.141 0.145 0.152

MAR3 -0.018 -0.012 -0.009 0.009 0.026 0.029 0.036

SMOK3 -0.030 -0.023 -0.020 -0.001 0.017 0.021 0.028

DK3 0.036 0.043 0.047 0.065 0.084 0.087 0.094

IAM3 -0.068 -0.062 -0.060 -0.045 -0.031 -0.028 -0.023

TPA3 -0.012 -0.005 -0.002 0.016 0.034 0.038 0.045

CM3 0.022 0.028 0.032 0.050 0.068 0.072 0.079

WX1 ON

AG1 -0.147 -0.138 -0.133 -0.106 -0.080 -0.075 -0.065

CX1 0.106 0.116 0.121 0.146 0.171 0.176 0.185

MAR1 0.003 0.014 0.020 0.051 0.081 0.087 0.099

SMOK1 -0.090 -0.079 -0.073 -0.044 -0.015 -0.009 0.002

DK1 -0.019 -0.009 -0.003 0.024 0.052 0.057 0.068

TPA1 -0.086 -0.076 -0.071 -0.045 -0.019 -0.014 -0.004

IAM1 -0.194 -0.178 -0.170 -0.127 -0.085 -0.076 -0.060

CM1 -0.031 -0.021 -0.016 0.012 0.039 0.044 0.054

WY1 ON

AG1 -0.095 -0.086 -0.082 -0.059 -0.036 -0.032 -0.023

CX1 -0.120 -0.112 -0.108 -0.087 -0.066 -0.062 -0.054

MAR1 -0.150 -0.140 -0.135 -0.110 -0.084 -0.079 -0.069

SMOK1 0.018 0.027 0.031 0.055 0.078 0.082 0.091

DK1 -0.065 -0.057 -0.052 -0.030 -0.007 -0.003 0.006

TPA1 -0.012 -0.004 0.000 0.020 0.040 0.044 0.052

IAM1 0.245 0.255 0.261 0.290 0.318 0.324 0.335

CM1 0.178 0.188 0.193 0.218 0.243 0.248 0.258

WINT1 ON

AG1 -0.124 -0.117 -0.113 -0.093 -0.073 -0.069 -0.061

CX1 0.178 0.187 0.192 0.216 0.239 0.244 0.253

MAR1 -0.061 -0.054 -0.051 -0.031 -0.012 -0.008 -0.001

SMOK1 -0.076 -0.066 -0.062 -0.036 -0.011 -0.007 0.003

DK1 0.048 0.057 0.061 0.085 0.109 0.114 0.123

TPA1 -0.029 -0.022 -0.019 -0.001 0.016 0.020 0.026

IAM1 -0.017 -0.013 -0.011 -0.001 0.008 0.010 0.014

CM1 -0.054 -0.047 -0.043 -0.023 -0.003 0.001 0.008

WX1 WITH

WY1 -0.198 -0.185 -0.179 -0.146 -0.112 -0.106 -0.094

WINT1 0.110 0.118 0.123 0.146 0.169 0.173 0.182

RIX 0.000 0.000 0.000 0.000 0.000 0.000 0.000

RIY 0.000 0.000 0.000 0.000 0.000 0.000 0.000

RIINT 0.000 0.000 0.000 0.000 0.000 0.000 0.000

WY1 WITH

WINT1 -0.070 -0.062 -0.058 -0.037 -0.017 -0.013 -0.005

RIX 0.000 0.000 0.000 0.000 0.000 0.000 0.000

RIY 0.000 0.000 0.000 0.000 0.000 0.000 0.000

WX2 WITH

WY2 -0.253 -0.240 -0.233 -0.198 -0.162 -0.155 -0.142

WINT2 0.074 0.083 0.087 0.109 0.131 0.135 0.143

WY2 WITH

WINT2 -0.074 -0.066 -0.063 -0.042 -0.022 -0.018 -0.011

WX3 WITH

WY3 -0.216 -0.206 -0.201 -0.173 -0.146 -0.140 -0.130

WINT3 0.076 0.084 0.089 0.112 0.135 0.140 0.148

WY3 WITH

WINT3 -0.080 -0.072 -0.068 -0.046 -0.024 -0.019 -0.011

RIINT WITH

WINT1 0.000 0.000 0.000 0.000 0.000 0.000 0.000

Intercepts

X1 2.155 2.242 2.286 2.518 2.749 2.794 2.880

X2 2.330 2.419 2.464 2.701 2.939 2.984 3.073

X3 2.139 2.221 2.263 2.481 2.700 2.742 2.824

Y1 2.081 2.163 2.205 2.425 2.644 2.686 2.769

Y2 2.322 2.407 2.451 2.678 2.906 2.949 3.034

Y3 1.767 1.856 1.901 2.137 2.374 2.419 2.508

INT1 -0.414 -0.329 -0.286 -0.059 0.167 0.211 0.296

INT2 0.363 0.450 0.494 0.727 0.960 1.004 1.091

INT3 2.807 2.895 2.940 3.174 3.409 3.454 3.542

Residual Variances

X1 0.000 0.000 0.000 0.000 0.000 0.000 0.000

X2 0.000 0.000 0.000 0.000 0.000 0.000 0.000

X3 0.000 0.000 0.000 0.000 0.000 0.000 0.000

Y1 0.000 0.000 0.000 0.000 0.000 0.000 0.000

Y2 0.000 0.000 0.000 0.000 0.000 0.000 0.000

Y3 0.000 0.000 0.000 0.000 0.000 0.000 0.000

INT1 0.000 0.000 0.000 0.000 0.000 0.000 0.000

INT2 0.000 0.000 0.000 0.000 0.000 0.000 0.000

INT3 0.000 0.000 0.000 0.000 0.000 0.000 0.000

RIX 0.483 0.494 0.499 0.529 0.558 0.564 0.575

RIINT 0.472 0.508 0.527 0.624 0.720 0.739 0.775

RIY 0.863 0.870 0.874 0.893 0.913 0.916 0.924

WX1 0.909 0.916 0.919 0.936 0.953 0.956 0.962

WX2 0.769 0.777 0.781 0.802 0.824 0.828 0.836

WX3 0.721 0.730 0.734 0.758 0.782 0.787 0.796

WINT1 0.914 0.918 0.921 0.932 0.944 0.946 0.951

WINT2 0.829 0.835 0.838 0.853 0.868 0.871 0.876

WINT3 0.756 0.761 0.764 0.778 0.792 0.795 0.801

WY1 0.793 0.801 0.805 0.825 0.846 0.850 0.858

WY2 0.752 0.760 0.764 0.786 0.808 0.813 0.821

WY3 0.705 0.714 0.718 0.742 0.765 0.770 0.779

CONFIDENCE INTERVALS OF TOTAL, TOTAL INDIRECT, SPECIFIC INDIRECT, AND DIRECT EFFECTS

Lower .5% Lower 2.5% Lower 5% Estimate Upper 5% Upper 2.5% Upper .5%

Effects from WX1 to WY3

Total -0.137 -0.130 -0.126 -0.105 -0.085 -0.081 -0.073

Total indirect -0.137 -0.130 -0.126 -0.105 -0.085 -0.081 -0.073

Specific indirect 1

WY3

WX2

WX1 -0.084 -0.079 -0.076 -0.061 -0.046 -0.043 -0.038

Specific indirect 2

WY3

WINT2

WX1 -0.015 -0.013 -0.012 -0.007 -0.002 -0.001 0.001

Specific indirect 3

WY3

WY2

WX1 -0.051 -0.048 -0.046 -0.037 -0.028 -0.027 -0.023

Effects from WY1 to WX3

Total -0.041 -0.038 -0.037 -0.031 -0.025 -0.024 -0.021

Total indirect -0.041 -0.038 -0.037 -0.031 -0.025 -0.024 -0.021

Specific indirect 1

WX3

WX2

WY1 -0.024 -0.023 -0.022 -0.018 -0.015 -0.014 -0.013

Specific indirect 2

WX3

WINT2

WY1 -0.003 -0.002 -0.002 -0.001 0.000 0.000 0.000

Specific indirect 3

WX3

WY2

WY1 -0.016 -0.015 -0.014 -0.011 -0.008 -0.007 -0.006

CONFIDENCE INTERVALS OF STANDARDIZED TOTAL, TOTAL INDIRECT, SPECIFIC INDIRECT, AND DIRECT EFFECTS

STDYX Standardization

Lower .5% Lower 2.5% Lower 5% Estimate Upper 5% Upper 2.5% Upper .5%

Effects from WX1 to WY3

Total -0.063 -0.059 -0.057 -0.048 -0.038 -0.037 -0.033

Total indirect -0.063 -0.059 -0.057 -0.048 -0.038 -0.037 -0.033

Specific indirect 1

WY3

WX2

WX1 -0.039 -0.036 -0.035 -0.028 -0.021 -0.019 -0.017

Specific indirect 2

WY3

WINT2

WX1 -0.007 -0.006 -0.005 -0.003 -0.001 -0.001 0.000

Specific indirect 3

WY3

WY2

WX1 -0.023 -0.022 -0.021 -0.017 -0.013 -0.012 -0.011

Effects from WY1 to WX3

Total -0.070 -0.066 -0.064 -0.053 -0.042 -0.040 -0.036

Total indirect -0.070 -0.066 -0.064 -0.053 -0.042 -0.040 -0.036

Specific indirect 1

WX3

WX2

WY1 -0.041 -0.039 -0.038 -0.031 -0.025 -0.024 -0.022

Specific indirect 2

WX3

WINT2

WY1 -0.004 -0.004 -0.004 -0.002 -0.001 -0.001 0.000

Specific indirect 3

WX3

WY2

WY1 -0.028 -0.026 -0.025 -0.019 -0.014 -0.012 -0.010

DIAGRAM INFORMATION

Use View Diagram under the Diagram menu in the Mplus Editor to view the diagram.

If running Mplus from the Mplus Diagrammer, the diagram opens automatically.

Diagram output

t:\mplus\shuj\测试3.dgm

Beginning Time: 15:48:16

Ending Time: 15:56:51

Elapsed Time: 00:08:35

Age 65 and over group , Reuslts:

(age65+)subgroup:

THE MODEL ESTIMATION TERMINATED NORMALLY

MODEL FIT INFORMATION

Number of Free Parameters 117

Loglikelihood

H0 Value -30056.517

H1 Value -29804.503

Information Criteria

Akaike (AIC) 60347.034

Bayesian (BIC) 61030.626

Sample-Size Adjusted BIC 60658.886

(n* = (n + 2) / 24)

Chi-Square Test of Model Fit

Value 504.028

Degrees of Freedom 171

P-Value 0.0000

RMSEA (Root Mean Square Error Of Approximation)

Estimate 0.028

90 Percent C.I. 0.025 0.030

Probability RMSEA <= .05 1.000

CFI/TLI

CFI 0.955

TLI 0.928

Chi-Square Test of Model Fit for the Baseline Model

Value 7604.463

Degrees of Freedom 270

P-Value 0.0000

SRMR (Standardized Root Mean Square Residual)

Value 0.028

MODEL RESULTS

Two-Tailed

Estimate S.E. Est./S.E. P-Value

RIX BY

X1 1.000 0.000 999.000 999.000

X2 1.000 0.000 999.000 999.000

X3 1.000 0.000 999.000 999.000

RIINT BY

INT1 1.000 0.000 999.000 999.000

INT2 1.000 0.000 999.000 999.000

INT3 1.000 0.000 999.000 999.000

RIY BY

Y1 1.000 0.000 999.000 999.000

Y2 1.000 0.000 999.000 999.000

Y3 1.000 0.000 999.000 999.000

WX1 BY

X1 1.000 0.000 999.000 999.000

WX2 BY

X2 1.000 0.000 999.000 999.000

WX3 BY

X3 1.000 0.000 999.000 999.000

WINT1 BY

INT1 1.000 0.000 999.000 999.000

WINT2 BY

INT2 1.000 0.000 999.000 999.000

WINT3 BY

INT3 1.000 0.000 999.000 999.000

WY1 BY

Y1 1.000 0.000 999.000 999.000

WY2 BY

Y2 1.000 0.000 999.000 999.000

WY3 BY

Y3 1.000 0.000 999.000 999.000

WX2 ON

WX1 0.379 0.038 9.904 0.000

WY1 -0.035 0.016 -2.200 0.028

WINT1 0.540 0.261 2.069 0.039

WY2 ON

WY1 0.098 0.041 2.388 0.017

WX1 -0.244 0.052 -4.720 0.000

WINT1 -1.195 0.465 -2.571 0.010

WINT2 ON

WINT1 0.582 0.059 9.803 0.000

WX1 0.006 0.002 4.055 0.000

WY1 -0.001 0.001 -1.807 0.071

WX3 ON

WX2 0.379 0.038 9.904 0.000

WY2 -0.035 0.016 -2.200 0.028

WINT2 0.540 0.261 2.069 0.039

WY3 ON

WY2 0.098 0.041 2.388 0.017

WX2 -0.244 0.052 -4.720 0.000

WINT2 -1.195 0.465 -2.571 0.010

WINT3 ON

WINT2 0.582 0.059 9.803 0.000

WX2 0.006 0.002 4.055 0.000

WY2 -0.001 0.001 -1.807 0.071

RIX ON

SEX 0.550 0.150 3.672 0.000

EDU 2.296 0.102 22.514 0.000

RIY ON

SEX -1.184 0.232 -5.105 0.000

EDU -0.436 0.150 -2.903 0.004

RIINT ON

SEX 0.002 0.006 0.377 0.706

EDU 0.030 0.005 5.823 0.000

WX2 ON

AG2 -0.076 0.020 -3.720 0.000

CX2 1.093 0.173 6.318 0.000

MAR2 0.623 0.209 2.979 0.003

SMOK2 -0.509 0.200 -2.550 0.011

DK2 0.034 0.173 0.199 0.842

TPA2 0.066 0.153 0.432 0.666

CM2 0.037 0.041 0.906 0.365

IAM2 -0.212 0.040 -5.325 0.000

WY2 ON

AG2 -0.075 0.028 -2.654 0.008

CX2 -1.298 0.258 -5.030 0.000

MAR2 -0.561 0.289 -1.944 0.052

SMOK2 0.398 0.271 1.468 0.142

DK2 -0.836 0.240 -3.482 0.000

TPA2 0.289 0.216 1.337 0.181

CM2 0.489 0.068 7.183 0.000

IAM2 0.428 0.058 7.334 0.000

WINT2 ON

AG2 -0.001 0.001 -0.977 0.329

CX2 0.069 0.012 5.679 0.000

MAR2 0.000 0.008 0.010 0.992

SMOK2 0.000 0.008 0.005 0.996

DK2 0.026 0.009 2.773 0.006

TPA2 0.003 0.005 0.515 0.607

CM2 0.003 0.002 1.548 0.122

IAM2 -0.001 0.001 -1.784 0.074

WX3 ON

AG3 -0.051 0.017 -3.043 0.002

CX3 0.482 0.152 3.182 0.001

MAR3 0.372 0.162 2.296 0.022

SMOK3 0.001 0.161 0.008 0.994

DK3 0.352 0.140 2.507 0.012

IAM3 -0.178 0.030 -5.908 0.000

TPA3 0.287 0.116 2.469 0.014

CM3 0.028 0.030 0.912 0.362

WY3 ON

AG3 -0.129 0.028 -4.607 0.000

CX3 -1.286 0.269 -4.773 0.000

MAR3 -0.852 0.287 -2.968 0.003

SMOK3 -0.232 0.289 -0.803 0.422

DK3 -0.411 0.235 -1.749 0.080

IAM3 0.441 0.050 8.837 0.000

TPA3 0.306 0.202 1.515 0.130

CM3 0.412 0.063 6.500 0.000

WINT3 ON

AG3 -0.005 0.001 -4.376 0.000

CX3 0.113 0.016 7.038 0.000

MAR3 0.012 0.012 0.987 0.323

SMOK3 0.025 0.015 1.708 0.088

DK3 0.031 0.014 2.243 0.025

IAM3 -0.005 0.001 -4.470 0.000

TPA3 -0.011 0.009 -1.154 0.249

CM3 0.007 0.003 2.268 0.023

WX1 ON

AG1 -0.102 0.015 -6.925 0.000

CX1 0.974 0.153 6.369 0.000

MAR1 0.343 0.189 1.816 0.069

SMOK1 -0.343 0.166 -2.069 0.039

DK1 -0.019 0.141 -0.134 0.893

TPA1 -0.089 0.086 -1.031 0.302

IAM1 -0.238 0.034 -7.083 0.000

CM1 0.004 0.043 0.093 0.926

WY1 ON

AG1 -0.098 0.025 -4.004 0.000

CX1 -1.786 0.244 -7.316 0.000

MAR1 -1.612 0.307 -5.258 0.000

SMOK1 0.000 0.275 -0.001 0.999

DK1 0.033 0.231 0.142 0.887

TPA1 0.048 0.148 0.322 0.747

IAM1 0.617 0.055 11.224 0.000

CM1 0.750 0.075 10.039 0.000

WINT1 ON

AG1 0.000 0.001 -0.516 0.606

CX1 0.039 0.008 4.837 0.000

MAR1 0.000 0.006 0.031 0.976

SMOK1 -0.006 0.007 -0.859 0.390

DK1 0.016 0.007 2.266 0.023

TPA1 0.000 0.003 0.007 0.995

IAM1 0.000 0.000 -0.558 0.577

CM1 0.002 0.002 0.707 0.480

WX1 WITH

WY1 -1.542 0.416 -3.704 0.000

WINT1 0.012 0.006 2.019 0.043

RIX 0.000 0.000 999.000 999.000

RIY 0.000 0.000 999.000 999.000

RIINT 0.000 0.000 999.000 999.000

WY1 WITH

WINT1 -0.022 0.010 -2.102 0.036

RIX 0.000 0.000 999.000 999.000

RIY 0.000 0.000 999.000 999.000

WX2 WITH

WY2 -1.748 0.516 -3.386 0.001

WINT2 0.034 0.011 3.167 0.002

WY2 WITH

WINT2 -0.043 0.015 -2.927 0.003

WX3 WITH

WY3 -1.794 0.369 -4.858 0.000

WINT3 0.072 0.015 4.764 0.000

WY3 WITH

WINT3 -0.066 0.028 -2.362 0.018

RIINT WITH

WINT1 0.000 0.000 999.000 999.000

Intercepts

X1 11.931 1.120 10.657 0.000

X2 11.704 1.663 7.039 0.000

X3 10.385 1.420 7.316 0.000

Y1 17.678 1.873 9.437 0.000

Y2 14.560 2.266 6.426 0.000

Y3 19.626 2.279 8.613 0.000

INT1 -0.076 0.045 -1.703 0.089

INT2 -0.068 0.061 -1.111 0.266

INT3 0.285 0.102 2.798 0.005

Residual Variances

X1 0.000 0.000 999.000 999.000

X2 0.000 0.000 999.000 999.000

X3 0.000 0.000 999.000 999.000

Y1 0.000 0.000 999.000 999.000

Y2 0.000 0.000 999.000 999.000

Y3 0.000 0.000 999.000 999.000

INT1 0.000 0.000 999.000 999.000

INT2 0.000 0.000 999.000 999.000

INT3 0.000 0.000 999.000 999.000

RIX 3.663 0.339 10.795 0.000

RIINT 0.005 0.002 2.124 0.034

RIY 12.317 0.962 12.807 0.000

WX1 4.630 0.335 13.821 0.000

WX2 8.337 0.385 21.674 0.000

WX3 6.112 0.260 23.526 0.000

WINT1 0.013 0.003 5.181 0.000

WINT2 0.029 0.003 9.797 0.000

WINT3 0.085 0.004 20.681 0.000

WY1 18.616 1.053 17.673 0.000

WY2 20.567 1.087 18.929 0.000

WY3 19.700 0.949 20.762 0.000

STANDARDIZED MODEL RESULTS

STDYX Standardization

Two-Tailed

Estimate S.E. Est./S.E. P-Value

RIX BY

X1 0.698 0.021 33.413 0.000

X2 0.576 0.020 29.010 0.000

X3 0.600 0.021 28.994 0.000

RIINT BY

INT1 0.520 0.113 4.600 0.000

INT2 0.348 0.081 4.287 0.000

INT3 0.212 0.049 4.301 0.000

RIY BY

Y1 0.578 0.022 26.081 0.000

Y2 0.563 0.022 26.104 0.000

Y3 0.559 0.021 26.067 0.000

WX1 BY

X1 0.651 0.021 31.475 0.000

WX2 BY

X2 0.759 0.015 51.301 0.000

WX3 BY

X3 0.736 0.017 44.531 0.000

WINT1 BY

INT1 0.845 0.063 13.333 0.000

WINT2 BY

INT2 0.927 0.029 31.855 0.000

WINT3 BY

INT3 0.969 0.011 91.218 0.000

WY1 BY

Y1 0.802 0.016 49.243 0.000

WY2 BY

Y2 0.814 0.015 53.587 0.000

WY3 BY

Y3 0.815 0.015 54.794 0.000

WX2 ON

WX1 0.269 0.029 9.144 0.000

WY1 -0.052 0.024 -2.144 0.032

WINT1 0.019 0.009 2.080 0.038

WY2 ON

WY1 0.094 0.039 2.387 0.017

WX1 -0.113 0.025 -4.512 0.000

WINT1 -0.027 0.011 -2.428 0.015

WINT2 ON

WINT1 0.355 0.049 7.215 0.000

WX1 0.077 0.019 4.097 0.000

WY1 -0.034 0.018 -1.844 0.065

WX3 ON

WX2 0.407 0.041 10.020 0.000

WY2 -0.058 0.027 -2.172 0.030

WINT2 0.033 0.016 2.081 0.037

WY3 ON

WY2 0.097 0.041 2.367 0.018

WX2 -0.158 0.033 -4.792 0.000

WINT2 -0.044 0.017 -2.593 0.010

WINT3 ON

WINT2 0.339 0.041 8.234 0.000

WX2 0.064 0.015 4.123 0.000

WY2 -0.021 0.011 -1.821 0.069

RIX ON

SEX 0.107 0.029 3.719 0.000

EDU 0.623 0.024 25.612 0.000

RIY ON

SEX -0.165 0.032 -5.113 0.000

EDU -0.085 0.029 -2.918 0.004

RIINT ON

SEX 0.017 0.055 0.304 0.761

EDU 0.297 0.138 2.155 0.031

WX2 ON

AG2 -0.116 0.032 -3.687 0.000

CX2 0.139 0.022 6.369 0.000

MAR2 0.081 0.027 2.995 0.003

SMOK2 -0.066 0.026 -2.548 0.011

DK2 0.005 0.024 0.198 0.843

TPA2 0.011 0.027 0.432 0.666

CM2 0.022 0.024 0.909 0.363

IAM2 -0.224 0.040 -5.537 0.000

WY2 ON

AG2 -0.075 0.029 -2.624 0.009

CX2 -0.107 0.022 -4.975 0.000

MAR2 -0.048 0.025 -1.937 0.053

SMOK2 0.034 0.023 1.471 0.141

DK2 -0.075 0.021 -3.480 0.001

TPA2 0.033 0.025 1.337 0.181

CM2 0.186 0.027 6.928 0.000

IAM2 0.294 0.039 7.594 0.000

WINT2 ON

AG2 -0.017 0.017 -0.987 0.324

CX2 0.155 0.025 6.283 0.000

MAR2 0.000 0.019 0.010 0.992

SMOK2 0.000 0.018 0.005 0.996

DK2 0.064 0.022 2.858 0.004

TPA2 0.008 0.016 0.515 0.606

CM2 0.034 0.022 1.560 0.119

IAM2 -0.017 0.009 -1.835 0.067

WX3 ON

AG3 -0.082 0.027 -3.020 0.003

CX3 0.070 0.022 3.153 0.002

MAR3 0.054 0.024 2.272 0.023

SMOK3 0.000 0.022 0.008 0.994

DK3 0.052 0.021 2.495 0.013

IAM3 -0.228 0.038 -5.991 0.000

TPA3 0.057 0.023 2.488 0.013

CM3 0.019 0.020 0.916 0.360

WY3 ON

AG3 -0.127 0.028 -4.557 0.000

CX3 -0.112 0.024 -4.712 0.000

MAR3 -0.074 0.025 -2.973 0.003

SMOK3 -0.019 0.024 -0.804 0.422

DK3 -0.036 0.021 -1.754 0.079

IAM3 0.339 0.036 9.499 0.000

TPA3 0.036 0.024 1.525 0.127

CM3 0.167 0.026 6.366 0.000

WINT3 ON

AG3 -0.081 0.018 -4.495 0.000

CX3 0.158 0.022 7.214 0.000

MAR3 0.017 0.017 0.989 0.323

SMOK3 0.033 0.020 1.706 0.088

DK3 0.043 0.019 2.229 0.026

IAM3 -0.059 0.013 -4.463 0.000

TPA3 -0.020 0.017 -1.156 0.248

CM3 0.046 0.020 2.266 0.023

WX1 ON

AG1 -0.219 0.031 -7.062 0.000

CX1 0.179 0.028 6.324 0.000

MAR1 0.058 0.032 1.821 0.069

SMOK1 -0.064 0.031 -2.069 0.039

DK1 -0.004 0.028 -0.134 0.893

TPA1 -0.027 0.026 -1.032 0.302

IAM1 -0.275 0.036 -7.649 0.000

CM1 0.003 0.028 0.094 0.925

WY1 ON

AG1 -0.102 0.025 -4.059 0.000

CX1 -0.158 0.021 -7.466 0.000

MAR1 -0.131 0.025 -5.321 0.000

SMOK1 0.000 0.025 -0.001 0.999

DK1 0.003 0.022 0.142 0.887

TPA1 0.007 0.022 0.322 0.747

IAM1 0.343 0.027 12.501 0.000

CM1 0.238 0.023 10.146 0.000

WINT1 ON

AG1 -0.013 0.025 -0.515 0.607

CX1 0.148 0.025 6.007 0.000

MAR1 0.001 0.020 0.031 0.976

SMOK1 -0.023 0.027 -0.851 0.395

DK1 0.063 0.027 2.341 0.019

TPA1 0.000 0.021 0.007 0.995

IAM1 -0.006 0.010 -0.557 0.577

CM1 0.020 0.028 0.717 0.473

WX1 WITH

WY1 -0.166 0.042 -3.927 0.000

WINT1 0.050 0.024 2.073 0.038

RIX 0.000 0.000 0.000 1.000

RIY 0.000 0.000 0.000 1.000

RIINT 0.000 0.000 0.000 1.000

WY1 WITH

WINT1 -0.044 0.020 -2.153 0.031

RIX 0.000 0.000 0.000 1.000

RIY 0.000 0.000 0.000 1.000

WX2 WITH

WY2 -0.133 0.040 -3.323 0.001

WINT2 0.069 0.021 3.216 0.001

WY2 WITH

WINT2 -0.056 0.019 -2.947 0.003

WX3 WITH

WY3 -0.163 0.033 -5.005 0.000

WINT3 0.100 0.021 4.860 0.000

WY3 WITH

WINT3 -0.051 0.022 -2.362 0.018

RIINT WITH

WINT1 0.000 0.000 0.000 1.000

Intercepts

X1 3.239 0.302 10.718 0.000

X2 2.624 0.372 7.047 0.000

X3 2.426 0.329 7.382 0.000

Y1 2.846 0.295 9.646 0.000

Y2 2.283 0.352 6.478 0.000

Y3 3.054 0.349 8.742 0.000

INT1 -0.552 0.316 -1.747 0.081

INT2 -0.330 0.298 -1.106 0.269

INT3 0.844 0.298 2.831 0.005

Residual Variances

X1 0.000 0.000 0.000 1.000

X2 0.000 0.000 0.000 1.000

X3 0.000 0.000 0.000 1.000

Y1 0.000 0.000 0.000 1.000

Y2 0.000 0.000 0.000 1.000

Y3 0.000 0.000 0.000 1.000

INT1 0.000 0.000 0.000 1.000

INT2 0.000 0.000 0.000 1.000

INT3 0.000 0.000 0.000 1.000

RIX 0.555 0.030 18.678 0.000

RIINT 0.908 0.171 5.324 0.000

RIY 0.956 0.013 72.902 0.000

WX1 0.804 0.028 28.636 0.000

WX2 0.728 0.028 26.290 0.000

WX3 0.615 0.031 19.808 0.000

WINT1 0.973 0.010 98.969 0.000

WINT2 0.806 0.034 23.586 0.000

WINT3 0.790 0.028 28.057 0.000

WY1 0.751 0.022 34.051 0.000

WY2 0.764 0.025 30.241 0.000

WY3 0.718 0.027 26.986 0.000

R-SQUARE

Observed Two-Tailed

Variable Estimate S.E. Est./S.E. P-Value

X1 1.000 0.000 999.000 999.000

X2 1.000 0.000 999.000 999.000

X3 1.000 0.000 999.000 999.000

Y1 1.000 0.000 999.000 999.000

Y2 1.000 0.000 999.000 999.000

Y3 1.000 0.000 999.000 999.000

INT1 1.000 0.000 999.000 999.000

INT2 1.000 0.000 999.000 999.000

INT3 1.000 0.000 999.000 999.000

Latent Two-Tailed

Variable Estimate S.E. Est./S.E. P-Value

RIX 0.445 0.030 14.988 0.000

RIINT 0.092 108.791 0.001 0.999

RIY 0.044 0.013 3.336 0.001

WX1 0.196 0.028 6.985 0.000

WX2 0.272 0.028 9.845 0.000

WX3 0.385 0.031 12.382 0.000

WINT1 0.027 0.010 2.780 0.005

WINT2 0.194 0.034 5.692 0.000

WINT3 0.210 0.028 7.450 0.000

WY1 0.249 0.022 11.320 0.000

WY2 0.236 0.025 9.340 0.000

WY3 0.282 0.027 10.605 0.000

TOTAL, TOTAL INDIRECT, SPECIFIC INDIRECT, AND DIRECT EFFECTS

Two-Tailed

Estimate S.E. Est./S.E. P-Value

Effects from WX1 to WY3

Total -0.124 0.025 -4.887 0.000

Total indirect -0.124 0.025 -4.887 0.000

Specific indirect 1

WY3

WX2

WX1 -0.093 0.021 -4.321 0.000

Specific indirect 2

WY3

WINT2

WX1 -0.007 0.003 -2.339 0.019

Specific indirect 3

WY3

WY2

WX1 -0.024 0.010 -2.329 0.020

Effects from WY1 to WX3

Total -0.017 0.007 -2.328 0.020

Total indirect -0.017 0.007 -2.328 0.020

Specific indirect 1

WX3

WX2

WY1 -0.013 0.006 -2.303 0.021

Specific indirect 2

WX3

WINT2

WY1 -0.001 0.001 -1.356 0.175

Specific indirect 3

WX3

WY2

WY1 -0.003 0.002 -1.540 0.124

STANDARDIZED TOTAL, TOTAL INDIRECT, SPECIFIC INDIRECT, AND DIRECT EFFECTS

STDYX Standardization

Two-Tailed

Estimate S.E. Est./S.E. P-Value

Effects from WX1 to WY3

Total -0.057 0.012 -4.762 0.000

Total indirect -0.057 0.012 -4.762 0.000

Specific indirect 1

WY3

WX2

WX1 -0.042 0.010 -4.102 0.000

Specific indirect 2

WY3

WINT2

WX1 -0.003 0.001 -2.328 0.020

Specific indirect 3

WY3

WY2

WX1 -0.011 0.005 -2.399 0.016

Effects from WY1 to WX3

Total -0.028 0.012 -2.278 0.023

Total indirect -0.028 0.012 -2.278 0.023

Specific indirect 1

WX3

WX2

WY1 -0.021 0.009 -2.282 0.022

Specific indirect 2

WX3

WINT2

WY1 -0.001 0.001 -1.352 0.177

Specific indirect 3

WX3

WY2

WY1 -0.005 0.004 -1.484 0.138

CONFIDENCE INTERVALS OF MODEL RESULTS

Lower .5% Lower 2.5% Lower 5% Estimate Upper 5% Upper 2.5% Upper .5%

RIX BY

X1 1.000 1.000 1.000 1.000 1.000 1.000 1.000

X2 1.000 1.000 1.000 1.000 1.000 1.000 1.000

X3 1.000 1.000 1.000 1.000 1.000 1.000 1.000

RIINT BY

INT1 1.000 1.000 1.000 1.000 1.000 1.000 1.000

INT2 1.000 1.000 1.000 1.000 1.000 1.000 1.000

INT3 1.000 1.000 1.000 1.000 1.000 1.000 1.000

RIY BY

Y1 1.000 1.000 1.000 1.000 1.000 1.000 1.000

Y2 1.000 1.000 1.000 1.000 1.000 1.000 1.000

Y3 1.000 1.000 1.000 1.000 1.000 1.000 1.000

WX1 BY

X1 1.000 1.000 1.000 1.000 1.000 1.000 1.000

WX2 BY

X2 1.000 1.000 1.000 1.000 1.000 1.000 1.000

WX3 BY

X3 1.000 1.000 1.000 1.000 1.000 1.000 1.000

WINT1 BY

INT1 1.000 1.000 1.000 1.000 1.000 1.000 1.000

WINT2 BY

INT2 1.000 1.000 1.000 1.000 1.000 1.000 1.000

WINT3 BY

INT3 1.000 1.000 1.000 1.000 1.000 1.000 1.000

WY1 BY

Y1 1.000 1.000 1.000 1.000 1.000 1.000 1.000

WY2 BY

Y2 1.000 1.000 1.000 1.000 1.000 1.000 1.000

WY3 BY

Y3 1.000 1.000 1.000 1.000 1.000 1.000 1.000

WX2 ON

WX1 0.281 0.304 0.316 0.379 0.442 0.454 0.478

WY1 -0.076 -0.066 -0.061 -0.035 -0.009 -0.004 0.006

WINT1 -0.132 0.029 0.111 0.540 0.970 1.052 1.213

WY2 ON

WY1 -0.008 0.018 0.031 0.098 0.166 0.179 0.204

WX1 -0.377 -0.345 -0.329 -0.244 -0.159 -0.143 -0.111

WINT1 -2.392 -2.106 -1.959 -1.195 -0.430 -0.284 0.002

WINT2 ON

WINT1 0.429 0.465 0.484 0.582 0.679 0.698 0.734

WX1 0.002 0.003 0.004 0.006 0.009 0.009 0.010

WY1 -0.003 -0.003 -0.002 -0.001 0.000 0.000 0.001

WX3 ON

WX2 0.281 0.304 0.316 0.379 0.442 0.454 0.478

WY2 -0.076 -0.066 -0.061 -0.035 -0.009 -0.004 0.006

WINT2 -0.132 0.029 0.111 0.540 0.970 1.052 1.213

WY3 ON

WY2 -0.008 0.018 0.031 0.098 0.166 0.179 0.204

WX2 -0.377 -0.345 -0.329 -0.244 -0.159 -0.143 -0.111

WINT2 -2.392 -2.106 -1.959 -1.195 -0.430 -0.284 0.002

WINT3 ON

WINT2 0.429 0.465 0.484 0.582 0.679 0.698 0.734

WX2 0.002 0.003 0.004 0.006 0.009 0.009 0.010

WY2 -0.003 -0.003 -0.002 -0.001 0.000 0.000 0.001

RIX ON

SEX 0.164 0.257 0.304 0.550 0.797 0.844 0.936

EDU 2.033 2.096 2.128 2.296 2.464 2.496 2.559

RIY ON

SEX -1.781 -1.638 -1.565 -1.184 -0.802 -0.729 -0.586

EDU -0.823 -0.730 -0.683 -0.436 -0.189 -0.142 -0.049

RIINT ON

SEX -0.014 -0.010 -0.008 0.002 0.013 0.015 0.019

EDU 0.017 0.020 0.022 0.030 0.039 0.041 0.044

WX2 ON

AG2 -0.129 -0.116 -0.110 -0.076 -0.042 -0.036 -0.023

CX2 0.647 0.754 0.808 1.093 1.377 1.432 1.538

MAR2 0.084 0.213 0.279 0.623 0.966 1.032 1.161

SMOK2 -1.023 -0.900 -0.838 -0.509 -0.181 -0.118 0.005

DK2 -0.411 -0.305 -0.250 0.034 0.319 0.374 0.480

TPA2 -0.328 -0.234 -0.186 0.066 0.318 0.366 0.460

CM2 -0.068 -0.043 -0.030 0.037 0.104 0.117 0.142

IAM2 -0.315 -0.290 -0.278 -0.212 -0.147 -0.134 -0.109

WY2 ON

AG2 -0.148 -0.131 -0.122 -0.075 -0.029 -0.020 -0.002

CX2 -1.962 -1.803 -1.722 -1.298 -0.873 -0.792 -0.633

MAR2 -1.304 -1.127 -1.036 -0.561 -0.086 0.004 0.182

SMOK2 -0.301 -0.134 -0.048 0.398 0.845 0.930 1.097

DK2 -1.454 -1.306 -1.230 -0.836 -0.441 -0.365 -0.217

TPA2 -0.268 -0.135 -0.067 0.289 0.645 0.713 0.846

CM2 0.313 0.355 0.377 0.489 0.600 0.622 0.664

IAM2 0.277 0.313 0.332 0.428 0.523 0.542 0.578

WINT2 ON

AG2 -0.002 -0.002 -0.002 -0.001 0.000 0.001 0.001

CX2 0.038 0.045 0.049 0.069 0.089 0.093 0.100

MAR2 -0.022 -0.016 -0.014 0.000 0.014 0.017 0.022

SMOK2 -0.021 -0.016 -0.013 0.000 0.013 0.016 0.021

DK2 0.002 0.008 0.011 0.026 0.042 0.045 0.051

TPA2 -0.011 -0.008 -0.006 0.003 0.011 0.013 0.016

CM2 -0.002 -0.001 0.000 0.003 0.007 0.007 0.009

IAM2 -0.002 -0.002 -0.002 -0.001 0.000 0.000 0.000

WX3 ON

AG3 -0.093 -0.083 -0.078 -0.051 -0.023 -0.018 -0.008

CX3 0.092 0.185 0.233 0.482 0.732 0.779 0.873

MAR3 -0.045 0.054 0.105 0.372 0.638 0.689 0.789

SMOK3 -0.413 -0.314 -0.263 0.001 0.266 0.316 0.415

DK3 -0.010 0.077 0.121 0.352 0.583 0.627 0.714

IAM3 -0.256 -0.237 -0.228 -0.178 -0.128 -0.119 -0.100

TPA3 -0.012 0.059 0.096 0.287 0.478 0.515 0.586

CM3 -0.051 -0.032 -0.022 0.028 0.078 0.088 0.106

WY3 ON

AG3 -0.202 -0.185 -0.176 -0.129 -0.083 -0.074 -0.057

CX3 -1.979 -1.814 -1.729 -1.286 -0.843 -0.758 -0.592

MAR3 -1.591 -1.415 -1.324 -0.852 -0.380 -0.289 -0.113

SMOK3 -0.978 -0.800 -0.708 -0.232 0.244 0.335 0.513

DK3 -1.017 -0.872 -0.798 -0.411 -0.025 0.050 0.194

IAM3 0.312 0.343 0.359 0.441 0.523 0.539 0.569

TPA3 -0.215 -0.090 -0.026 0.306 0.639 0.703 0.828

CM3 0.249 0.288 0.308 0.412 0.517 0.537 0.576

WINT3 ON

AG3 -0.008 -0.007 -0.007 -0.005 -0.003 -0.003 -0.002

CX3 0.072 0.082 0.087 0.113 0.140 0.145 0.155

MAR3 -0.020 -0.012 -0.008 0.012 0.033 0.037 0.044

SMOK3 -0.013 -0.004 0.001 0.025 0.050 0.055 0.064

DK3 -0.005 0.004 0.008 0.031 0.053 0.057 0.066

IAM3 -0.008 -0.007 -0.007 -0.005 -0.003 -0.003 -0.002

TPA3 -0.034 -0.029 -0.026 -0.011 0.005 0.007 0.013

CM3 -0.001 0.001 0.002 0.007 0.012 0.013 0.015

WX1 ON

AG1 -0.140 -0.131 -0.126 -0.102 -0.078 -0.073 -0.064

CX1 0.580 0.675 0.723 0.974 1.226 1.274 1.369

MAR1 -0.144 -0.027 0.032 0.343 0.655 0.714 0.831

SMOK1 -0.770 -0.668 -0.616 -0.343 -0.070 -0.018 0.084

DK1 -0.381 -0.294 -0.250 -0.019 0.212 0.257 0.343

TPA1 -0.311 -0.258 -0.231 -0.089 0.053 0.080 0.133

IAM1 -0.325 -0.304 -0.294 -0.238 -0.183 -0.172 -0.152

CM1 -0.106 -0.080 -0.066 0.004 0.074 0.087 0.114

WY1 ON

AG1 -0.161 -0.146 -0.139 -0.098 -0.058 -0.050 -0.035

CX1 -2.415 -2.264 -2.187 -1.786 -1.384 -1.307 -1.157

MAR1 -2.402 -2.213 -2.116 -1.612 -1.108 -1.011 -0.822

SMOK1 -0.708 -0.539 -0.452 0.000 0.451 0.538 0.707

DK1 -0.562 -0.420 -0.347 0.033 0.413 0.485 0.628

TPA1 -0.334 -0.243 -0.196 0.048 0.292 0.338 0.430

IAM1 0.475 0.509 0.527 0.617 0.707 0.725 0.759

CM1 0.558 0.604 0.627 0.750 0.873 0.897 0.943

WINT1 ON

AG1 -0.002 -0.001 -0.001 0.000 0.001 0.001 0.001

CX1 0.018 0.023 0.026 0.039 0.052 0.055 0.060

MAR1 -0.015 -0.011 -0.009 0.000 0.010 0.012 0.015

SMOK1 -0.024 -0.019 -0.017 -0.006 0.005 0.008 0.012

DK1 -0.002 0.002 0.004 0.016 0.027 0.029 0.033

TPA1 -0.009 -0.007 -0.005 0.000 0.006 0.007 0.009

IAM1 -0.001 -0.001 -0.001 0.000 0.000 0.001 0.001

CM1 -0.004 -0.003 -0.002 0.002 0.005 0.006 0.007

WX1 WITH

WY1 -2.615 -2.359 -2.227 -1.542 -0.857 -0.726 -0.470

WINT1 -0.003 0.000 0.002 0.012 0.022 0.024 0.028

RIX 0.000 0.000 0.000 0.000 0.000 0.000 0.000

RIY 0.000 0.000 0.000 0.000 0.000 0.000 0.000

RIINT 0.000 0.000 0.000 0.000 0.000 0.000 0.000

WY1 WITH

WINT1 -0.048 -0.042 -0.039 -0.022 -0.005 -0.001 0.005

RIX 0.000 0.000 0.000 0.000 0.000 0.000 0.000

RIY 0.000 0.000 0.000 0.000 0.000 0.000 0.000

WX2 WITH

WY2 -3.078 -2.760 -2.597 -1.748 -0.899 -0.736 -0.418

WINT2 0.006 0.013 0.016 0.034 0.052 0.055 0.062

WY2 WITH

WINT2 -0.081 -0.072 -0.068 -0.043 -0.019 -0.014 -0.005

WX3 WITH

WY3 -2.745 -2.517 -2.401 -1.794 -1.186 -1.070 -0.843

WINT3 0.033 0.042 0.047 0.072 0.097 0.102 0.111

WY3 WITH

WINT3 -0.139 -0.121 -0.113 -0.066 -0.020 -0.011 0.006

RIINT WITH

WINT1 0.000 0.000 0.000 0.000 0.000 0.000 0.000

Intercepts

X1 9.048 9.737 10.090 11.931 13.773 14.126 14.815

X2 7.421 8.445 8.969 11.704 14.439 14.963 15.987

X3 6.728 7.603 8.050 10.385 12.720 13.167 14.041

Y1 12.852 14.006 14.596 17.678 20.759 21.349 22.503

Y2 8.723 10.119 10.833 14.560 18.287 19.001 20.396

Y3 13.757 15.160 15.878 19.626 23.374 24.092 25.495

INT1 -0.191 -0.164 -0.150 -0.076 -0.003 0.012 0.039

INT2 -0.225 -0.188 -0.168 -0.068 0.033 0.052 0.089

INT3 0.023 0.085 0.118 0.285 0.453 0.485 0.548

Residual Variances

X1 0.000 0.000 0.000 0.000 0.000 0.000 0.000

X2 0.000 0.000 0.000 0.000 0.000 0.000 0.000

X3 0.000 0.000 0.000 0.000 0.000 0.000 0.000

Y1 0.000 0.000 0.000 0.000 0.000 0.000 0.000

Y2 0.000 0.000 0.000 0.000 0.000 0.000 0.000

Y3 0.000 0.000 0.000 0.000 0.000 0.000 0.000

INT1 0.000 0.000 0.000 0.000 0.000 0.000 0.000

INT2 0.000 0.000 0.000 0.000 0.000 0.000 0.000

INT3 0.000 0.000 0.000 0.000 0.000 0.000 0.000

RIX 2.789 2.998 3.105 3.663 4.221 4.328 4.537

RIINT -0.001 0.000 0.001 0.005 0.008 0.009 0.010

RIY 9.840 10.432 10.735 12.317 13.899 14.202 14.794

WX1 3.767 3.974 4.079 4.630 5.181 5.287 5.493

WX2 7.346 7.583 7.704 8.337 8.969 9.091 9.327

WX3 5.443 5.603 5.685 6.112 6.540 6.622 6.782

WINT1 0.007 0.008 0.009 0.013 0.017 0.018 0.020

WINT2 0.022 0.023 0.024 0.029 0.034 0.035 0.037

WINT3 0.074 0.077 0.078 0.085 0.092 0.093 0.095

WY1 15.903 16.552 16.883 18.616 20.349 20.681 21.329

WY2 17.769 18.438 18.780 20.567 22.355 22.697 23.366

WY3 17.256 17.841 18.140 19.700 21.261 21.560 22.145

CONFIDENCE INTERVALS OF STANDARDIZED MODEL RESULTS

STDYX Standardization

Lower .5% Lower 2.5% Lower 5% Estimate Upper 5% Upper 2.5% Upper .5%

RIX BY

X1 0.644 0.657 0.663 0.698 0.732 0.738 0.751

X2 0.525 0.537 0.543 0.576 0.609 0.615 0.627

X3 0.547 0.560 0.566 0.600 0.634 0.641 0.654

RIINT BY

INT1 0.229 0.298 0.334 0.520 0.706 0.741 0.811

INT2 0.139 0.189 0.214 0.348 0.481 0.507 0.557

INT3 0.085 0.115 0.131 0.212 0.293 0.309 0.339

RIY BY

Y1 0.521 0.534 0.541 0.578 0.614 0.621 0.635

Y2 0.507 0.521 0.527 0.563 0.598 0.605 0.618

Y3 0.503 0.517 0.523 0.559 0.594 0.601 0.614

WX1 BY

X1 0.598 0.611 0.617 0.651 0.686 0.692 0.705

WX2 BY

X2 0.721 0.730 0.734 0.759 0.783 0.788 0.797

WX3 BY

X3 0.694 0.704 0.709 0.736 0.764 0.769 0.779

WINT1 BY

INT1 0.682 0.721 0.741 0.845 0.949 0.969 1.008

WINT2 BY

INT2 0.852 0.870 0.879 0.927 0.974 0.984 1.002

WINT3 BY

INT3 0.942 0.948 0.952 0.969 0.987 0.990 0.996

WY1 BY

Y1 0.760 0.770 0.775 0.802 0.829 0.834 0.844

WY2 BY

Y2 0.774 0.784 0.789 0.814 0.839 0.843 0.853

WY3 BY

Y3 0.777 0.786 0.791 0.815 0.840 0.844 0.854

WX2 ON

WX1 0.193 0.211 0.220 0.269 0.317 0.326 0.345

WY1 -0.114 -0.099 -0.091 -0.052 -0.012 -0.004 0.010

WINT1 -0.004 0.001 0.004 0.019 0.033 0.036 0.042

WY2 ON

WY1 -0.007 0.017 0.029 0.094 0.159 0.172 0.196

WX1 -0.177 -0.162 -0.154 -0.113 -0.072 -0.064 -0.048

WINT1 -0.055 -0.048 -0.045 -0.027 -0.009 -0.005 0.002

WINT2 ON

WINT1 0.228 0.258 0.274 0.355 0.436 0.451 0.482

WX1 0.029 0.040 0.046 0.077 0.109 0.114 0.126

WY1 -0.082 -0.070 -0.065 -0.034 -0.004 0.002 0.014

WX3 ON

WX2 0.303 0.328 0.340 0.407 0.474 0.487 0.512

WY2 -0.126 -0.110 -0.101 -0.058 -0.014 -0.006 0.011

WINT2 -0.008 0.002 0.007 0.033 0.059 0.064 0.073

WY3 ON

WY2 -0.009 0.017 0.030 0.097 0.165 0.178 0.203

WX2 -0.242 -0.222 -0.212 -0.158 -0.104 -0.093 -0.073

WINT2 -0.087 -0.076 -0.071 -0.044 -0.016 -0.011 0.000

WINT3 ON

WINT2 0.233 0.258 0.271 0.339 0.407 0.419 0.445

WX2 0.024 0.033 0.038 0.064 0.089 0.094 0.103

WY2 -0.050 -0.043 -0.039 -0.021 -0.002 0.002 0.009

RIX ON

SEX 0.033 0.051 0.060 0.107 0.154 0.163 0.181

EDU 0.561 0.576 0.583 0.623 0.663 0.671 0.686

RIY ON

SEX -0.248 -0.228 -0.218 -0.165 -0.112 -0.102 -0.082

EDU -0.159 -0.142 -0.132 -0.085 -0.037 -0.028 -0.010

RIINT ON

SEX -0.126 -0.092 -0.074 0.017 0.108 0.125 0.159

EDU -0.058 0.027 0.070 0.297 0.523 0.567 0.651

WX2 ON

AG2 -0.198 -0.178 -0.168 -0.116 -0.064 -0.055 -0.035

CX2 0.083 0.096 0.103 0.139 0.174 0.181 0.195

MAR2 0.011 0.028 0.037 0.081 0.126 0.134 0.151

SMOK2 -0.132 -0.116 -0.108 -0.066 -0.023 -0.015 0.001

DK2 -0.057 -0.042 -0.034 0.005 0.044 0.051 0.066

TPA2 -0.057 -0.041 -0.032 0.011 0.055 0.064 0.080

CM2 -0.040 -0.025 -0.017 0.022 0.061 0.068 0.083

IAM2 -0.328 -0.303 -0.290 -0.224 -0.157 -0.144 -0.120

WY2 ON

AG2 -0.149 -0.131 -0.122 -0.075 -0.028 -0.019 -0.001

CX2 -0.163 -0.150 -0.143 -0.107 -0.072 -0.065 -0.052

MAR2 -0.111 -0.096 -0.088 -0.048 -0.007 0.001 0.016

SMOK2 -0.025 -0.011 -0.004 0.034 0.071 0.078 0.092

DK2 -0.130 -0.117 -0.110 -0.075 -0.039 -0.033 -0.019

TPA2 -0.030 -0.015 -0.008 0.033 0.073 0.081 0.096

CM2 0.117 0.133 0.141 0.186 0.230 0.238 0.254

IAM2 0.194 0.218 0.230 0.294 0.358 0.370 0.394

WINT2 ON

AG2 -0.060 -0.050 -0.044 -0.017 0.011 0.016 0.027

CX2 0.092 0.107 0.115 0.155 0.196 0.204 0.219

MAR2 -0.050 -0.038 -0.032 0.000 0.032 0.038 0.050

SMOK2 -0.047 -0.036 -0.030 0.000 0.030 0.036 0.048

DK2 0.006 0.020 0.027 0.064 0.100 0.107 0.121

TPA2 -0.033 -0.023 -0.018 0.008 0.035 0.040 0.050

CM2 -0.022 -0.009 -0.002 0.034 0.069 0.076 0.090

IAM2 -0.042 -0.036 -0.033 -0.017 -0.002 0.001 0.007

WX3 ON

AG3 -0.153 -0.136 -0.127 -0.082 -0.037 -0.029 -0.012

CX3 0.013 0.026 0.033 0.070 0.106 0.113 0.127

MAR3 -0.007 0.007 0.015 0.054 0.093 0.100 0.115

SMOK3 -0.056 -0.043 -0.036 0.000 0.036 0.043 0.057

DK3 -0.002 0.011 0.018 0.052 0.086 0.093 0.105

IAM3 -0.325 -0.302 -0.290 -0.228 -0.165 -0.153 -0.130

TPA3 -0.002 0.012 0.019 0.057 0.094 0.101 0.115

CM3 -0.034 -0.021 -0.015 0.019 0.052 0.059 0.072

WY3 ON

AG3 -0.198 -0.181 -0.172 -0.127 -0.081 -0.072 -0.055

CX3 -0.174 -0.159 -0.151 -0.112 -0.073 -0.066 -0.051

MAR3 -0.139 -0.123 -0.115 -0.074 -0.033 -0.025 -0.010

SMOK3 -0.080 -0.066 -0.058 -0.019 0.020 0.027 0.042

DK3 -0.090 -0.077 -0.071 -0.036 -0.002 0.004 0.017

IAM3 0.247 0.269 0.280 0.339 0.398 0.409 0.431

TPA3 -0.025 -0.010 -0.003 0.036 0.076 0.083 0.098

CM3 0.100 0.116 0.124 0.167 0.210 0.219 0.235

WINT3 ON

AG3 -0.127 -0.116 -0.110 -0.081 -0.051 -0.045 -0.034

CX3 0.102 0.115 0.122 0.158 0.194 0.201 0.214

MAR3 -0.028 -0.017 -0.011 0.017 0.046 0.051 0.062

SMOK3 -0.017 -0.005 0.001 0.033 0.065 0.072 0.084

DK3 -0.007 0.005 0.011 0.043 0.075 0.081 0.093

IAM3 -0.093 -0.085 -0.081 -0.059 -0.037 -0.033 -0.025

TPA3 -0.065 -0.054 -0.049 -0.020 0.009 0.014 0.025

CM3 -0.006 0.006 0.013 0.046 0.079 0.086 0.098

WX1 ON

AG1 -0.299 -0.280 -0.270 -0.219 -0.168 -0.158 -0.139

CX1 0.106 0.124 0.132 0.179 0.226 0.235 0.252

MAR1 -0.024 -0.004 0.006 0.058 0.110 0.120 0.140

SMOK1 -0.144 -0.125 -0.115 -0.064 -0.013 -0.003 0.016

DK1 -0.075 -0.058 -0.049 -0.004 0.042 0.051 0.068

TPA1 -0.095 -0.079 -0.071 -0.027 0.016 0.025 0.041

IAM1 -0.367 -0.345 -0.334 -0.275 -0.216 -0.204 -0.182

CM1 -0.070 -0.052 -0.043 0.003 0.049 0.058 0.075

WY1 ON

AG1 -0.166 -0.151 -0.143 -0.102 -0.060 -0.053 -0.037

CX1 -0.213 -0.200 -0.193 -0.158 -0.123 -0.117 -0.104

MAR1 -0.194 -0.179 -0.171 -0.131 -0.090 -0.083 -0.067

SMOK1 -0.064 -0.049 -0.041 0.000 0.041 0.049 0.064

DK1 -0.053 -0.040 -0.033 0.003 0.039 0.046 0.060

TPA1 -0.049 -0.036 -0.029 0.007 0.043 0.050 0.063

IAM1 0.272 0.289 0.298 0.343 0.388 0.396 0.413

CM1 0.178 0.192 0.199 0.238 0.277 0.284 0.298

WINT1 ON

AG1 -0.077 -0.061 -0.054 -0.013 0.028 0.036 0.051

CX1 0.085 0.100 0.108 0.148 0.189 0.197 0.212

MAR1 -0.051 -0.039 -0.032 0.001 0.034 0.040 0.052

SMOK1 -0.092 -0.076 -0.067 -0.023 0.021 0.030 0.046

DK1 -0.006 0.010 0.019 0.063 0.108 0.117 0.133

TPA1 -0.054 -0.041 -0.035 0.000 0.035 0.042 0.055

IAM1 -0.032 -0.026 -0.022 -0.006 0.011 0.014 0.021

CM1 -0.053 -0.035 -0.026 0.020 0.067 0.076 0.094

WX1 WITH

WY1 -0.275 -0.249 -0.236 -0.166 -0.097 -0.083 -0.057

WINT1 -0.012 0.003 0.010 0.050 0.089 0.097 0.111

RIX 0.000 0.000 0.000 0.000 0.000 0.000 0.000

RIY 0.000 0.000 0.000 0.000 0.000 0.000 0.000

RIINT 0.000 0.000 0.000 0.000 0.000 0.000 0.000

WY1 WITH

WINT1 -0.096 -0.083 -0.077 -0.044 -0.010 -0.004 0.009

RIX 0.000 0.000 0.000 0.000 0.000 0.000 0.000

RIY 0.000 0.000 0.000 0.000 0.000 0.000 0.000

WX2 WITH

WY2 -0.237 -0.212 -0.200 -0.133 -0.067 -0.055 -0.030

WINT2 0.014 0.027 0.034 0.069 0.104 0.111 0.124

WY2 WITH

WINT2 -0.104 -0.093 -0.087 -0.056 -0.025 -0.019 -0.007

WX3 WITH

WY3 -0.248 -0.227 -0.217 -0.163 -0.110 -0.099 -0.079

WINT3 0.047 0.060 0.066 0.100 0.134 0.140 0.153

WY3 WITH

WINT3 -0.107 -0.094 -0.087 -0.051 -0.016 -0.009 0.005

RIINT WITH

WINT1 0.000 0.000 0.000 0.000 0.000 0.000 0.000

Intercepts

X1 2.460 2.647 2.742 3.239 3.736 3.831 4.017

X2 1.665 1.894 2.011 2.624 3.236 3.353 3.582

X3 1.580 1.782 1.886 2.426 2.967 3.070 3.273

Y1 2.086 2.268 2.361 2.846 3.331 3.424 3.606

Y2 1.375 1.592 1.703 2.283 2.863 2.974 3.191

Y3 2.154 2.369 2.480 3.054 3.629 3.739 3.954

INT1 -1.367 -1.172 -1.072 -0.552 -0.032 0.067 0.262

INT2 -1.097 -0.914 -0.820 -0.330 0.160 0.254 0.438

INT3 0.076 0.260 0.354 0.844 1.335 1.429 1.613

Residual Variances

X1 0.000 0.000 0.000 0.000 0.000 0.000 0.000

X2 0.000 0.000 0.000 0.000 0.000 0.000 0.000

X3 0.000 0.000 0.000 0.000 0.000 0.000 0.000

Y1 0.000 0.000 0.000 0.000 0.000 0.000 0.000

Y2 0.000 0.000 0.000 0.000 0.000 0.000 0.000

Y3 0.000 0.000 0.000 0.000 0.000 0.000 0.000

INT1 0.000 0.000 0.000 0.000 0.000 0.000 0.000

INT2 0.000 0.000 0.000 0.000 0.000 0.000 0.000

INT3 0.000 0.000 0.000 0.000 0.000 0.000 0.000

RIX 0.478 0.497 0.506 0.555 0.604 0.613 0.631

RIINT 0.469 0.574 0.628 0.908 1.189 1.243 1.348

RIY 0.922 0.931 0.935 0.956 0.978 0.982 0.990

WX1 0.732 0.749 0.758 0.804 0.850 0.859 0.876

WX2 0.656 0.673 0.682 0.728 0.773 0.782 0.799

WX3 0.535 0.554 0.564 0.615 0.666 0.676 0.695

WINT1 0.947 0.953 0.957 0.973 0.989 0.992 0.998

WINT2 0.718 0.739 0.749 0.806 0.862 0.873 0.894

WINT3 0.718 0.735 0.744 0.790 0.837 0.845 0.863

WY1 0.694 0.707 0.714 0.751 0.787 0.794 0.807

WY2 0.699 0.715 0.722 0.764 0.806 0.814 0.829

WY3 0.649 0.666 0.674 0.718 0.762 0.770 0.786

CONFIDENCE INTERVALS OF TOTAL, TOTAL INDIRECT, SPECIFIC INDIRECT, AND DIRECT EFFECTS

Lower .5% Lower 2.5% Lower 5% Estimate Upper 5% Upper 2.5% Upper .5%

Effects from WX1 to WY3

Total -0.189 -0.174 -0.166 -0.124 -0.082 -0.074 -0.059

Total indirect -0.189 -0.174 -0.166 -0.124 -0.082 -0.074 -0.059

Specific indirect 1

WY3

WX2

WX1 -0.148 -0.135 -0.128 -0.093 -0.057 -0.051 -0.037

Specific indirect 2

WY3

WINT2

WX1 -0.015 -0.014 -0.013 -0.007 -0.002 -0.001 0.001

Specific indirect 3

WY3

WY2

WX1 -0.050 -0.044 -0.041 -0.024 -0.007 -0.004 0.003

Effects from WY1 to WX3

Total -0.037 -0.032 -0.030 -0.017 -0.005 -0.003 0.002

Total indirect -0.037 -0.032 -0.030 -0.017 -0.005 -0.003 0.002

Specific indirect 1

WX3

WX2

WY1 -0.028 -0.025 -0.023 -0.013 -0.004 -0.002 0.002

Specific indirect 2

WX3

WINT2

WY1 -0.002 -0.002 -0.002 -0.001 0.000 0.000 0.001

Specific indirect 3

WX3

WY2

WY1 -0.009 -0.008 -0.007 -0.003 0.000 0.001 0.002

CONFIDENCE INTERVALS OF STANDARDIZED TOTAL, TOTAL INDIRECT, SPECIFIC INDIRECT, AND DIRECT EFFECTS

STDYX Standardization

Lower .5% Lower 2.5% Lower 5% Estimate Upper 5% Upper 2.5% Upper .5%

Effects from WX1 to WY3

Total -0.087 -0.080 -0.076 -0.057 -0.037 -0.033 -0.026

Total indirect -0.087 -0.080 -0.076 -0.057 -0.037 -0.033 -0.026

Specific indirect 1

WY3

WX2

WX1 -0.069 -0.063 -0.059 -0.042 -0.025 -0.022 -0.016

Specific indirect 2

WY3

WINT2

WX1 -0.007 -0.006 -0.006 -0.003 -0.001 -0.001 0.000

Specific indirect 3

WY3

WY2

WX1 -0.023 -0.020 -0.018 -0.011 -0.003 -0.002 0.001

Effects from WY1 to WX3

Total -0.059 -0.051 -0.047 -0.028 -0.008 -0.004 0.004

Total indirect -0.059 -0.051 -0.047 -0.028 -0.008 -0.004 0.004

Specific indirect 1

WX3

WX2

WY1 -0.045 -0.039 -0.036 -0.021 -0.006 -0.003 0.003

Specific indirect 2

WX3

WINT2

WY1 -0.003 -0.003 -0.002 -0.001 0.000 0.001 0.001

Specific indirect 3

WX3

WY2

WY1 -0.015 -0.013 -0.011 -0.005 0.001 0.002 0.004

DIAGRAM INFORMATION

Use View Diagram under the Diagram menu in the Mplus Editor to view the diagram.

If running Mplus from the Mplus Diagrammer, the diagram opens automatically.

Diagram output

t:\mplus\shuj\测试.dgm

Beginning Time: 15:49:55

Ending Time: 16:01:17

Elapsed Time: 00:11:22

MUTHEN & MUTHEN

3463 Stoner Ave.

Los Angeles, CA 90066

Tel: (310) 391-9971

Fax: (310) 391-8971

Web: www.StatModel.com

Support: Support@StatModel.com

Copyright (c) 1998-2019 Muthen & Muthen.
