# Supplementary material for: Mediating Role of Internet Use in Cognitive-Depressive Pathways: A Random Intercept Cross-Lagged Panel Modeling Approach
Source: Int J Public Health. 2025 Oct 21;70:1608478. doi: 10.3389/ijph.2025.1608478 (PMC12583110; doi:10.3389/ijph.2025.1608478)
Supplement: Supplementary file 2 [file Supplementaryfile3.doc]

Tests of Measurement Invariance.

Cognition function scale (2015-2020)

TITLE: SEM-jchg

Data:

file is CLPMC1.dat;

FORMAT IS FREE;

TYPE IS INDIVIDUAL;

VARIABLE:

Names = id1 am1-am3 xq1-xq9 yq1-yq30 sex age1-age3

cx1-cx3 edu1-edu3 mar1-mar3 smok1-smok3;

USEvar = xq1-xq9;

Grouping=sex(1=male 0=female);

model:

x1 by xq1-xq3;

x2 by xq4-xq6;

x3 by xq7-xq9;

analysis: estimator=ML;

model=configural metric scalar;

Output: standardized;

MODEL FIT INFORMATION

Invariance Testing

Number of Degrees of

Model Parameters Chi-Square Freedom P-Value

Configural 60 3197.417 48 0.0000

Metric 54 3222.501 54 0.0000

Scalar 48 4514.839 60 0.0000

Degrees of

Models Compared Chi-Square Freedom P-Value

Metric against Configural 25.084 6 0.0003

Scalar against Configural 1317.423 12 0.0000

Scalar against Metric 1292.339 6 0.0000

MODEL FIT INFORMATION FOR THE CONFIGURAL MODEL

Number of Free Parameters 60

Loglikelihood

H0 Value -281139.069

H1 Value -279540.361

Information Criteria

Akaike (AIC) 562398.139

Bayesian (BIC) 562861.801

Sample-Size Adjusted BIC 562671.125

(n* = (n + 2) / 24)

Chi-Square Test of Model Fit

Value 3197.417

Degrees of Freedom 48

P-Value 0.0000

Chi-Square Contribution and P-Value From Each Group (degrees of freedom = 24)

FEMALE 1714.370 0.000

MALE 1483.047 0.000

RMSEA (Root Mean Square Error Of Approximation)

Estimate 0.088

90 Percent C.I. 0.086 0.091

Probability RMSEA <= .05 0.000

CFI/TLI

CFI 0.951

TLI 0.927

Chi-Square Test of Model Fit for the Baseline Model

Value 64900.435

Degrees of Freedom 72

P-Value 0.0000

SRMR (Standardized Root Mean Square Residual)

Value 0.030

MODEL FIT INFORMATION FOR THE METRIC MODEL

Number of Free Parameters 54

Loglikelihood

H0 Value -281151.611

H1 Value -279540.361

Information Criteria

Akaike (AIC) 562411.223

Bayesian (BIC) 562828.519

Sample-Size Adjusted BIC 562656.910

(n* = (n + 2) / 24)

Chi-Square Test of Model Fit

Value 3222.501

Degrees of Freedom 54

P-Value 0.0000

Chi-Square Contribution From Each Group

FEMALE 1724.987

MALE 1497.514

RMSEA (Root Mean Square Error Of Approximation)

Estimate 0.084

90 Percent C.I. 0.081 0.086

Probability RMSEA <= .05 0.000

CFI/TLI

CFI 0.951

TLI 0.935

Chi-Square Test of Model Fit for the Baseline Model

Value 64900.435

Degrees of Freedom 72

P-Value 0.0000

SRMR (Standardized Root Mean Square Residual)

Value 0.031

MODEL FIT INFORMATION FOR THE SCALAR MODEL

Number of Free Parameters 48

Loglikelihood

H0 Value -281797.781

H1 Value -279540.361

Information Criteria

Akaike (AIC) 563691.562

Bayesian (BIC) 564062.492

Sample-Size Adjusted BIC 563909.951

(n* = (n + 2) / 24)

Chi-Square Test of Model Fit

Value 4514.839

Degrees of Freedom 60

P-Value 0.0000

Chi-Square Contribution From Each Group

FEMALE 2325.099

MALE 2189.741

RMSEA (Root Mean Square Error Of Approximation)

Estimate 0.092

90 Percent C.I. 0.090 0.094

Probability RMSEA <= .05 0.000

CFI/TLI

CFI 0.941

TLI 0.924

Chi-Square Test of Model Fit for the Baseline Model

Value 64900.435

Degrees of Freedom 72

P-Value 0.0000

SRMR (Standardized Root Mean Square Residual)

Value 0.040

MODEL RESULTS FOR THE CONFIGURAL MODEL

Two-Tailed

Estimate S.E. Est./S.E. P-Value

Group FEMALE

X1 BY

XQ1 1.000 0.000 999.000 999.000

XQ2 0.499 0.008 60.227 0.000

XQ3 0.138 0.002 58.293 0.000

X2 BY

XQ4 1.000 0.000 999.000 999.000

XQ5 0.924 0.013 69.523 0.000

XQ6 0.198 0.003 60.672 0.000

X3 BY

XQ7 1.000 0.000 999.000 999.000

XQ8 0.772 0.010 77.830 0.000

XQ9 0.135 0.002 60.566 0.000

X2 WITH

X1 7.258 0.184 39.360 0.000

X3 WITH

X1 7.043 0.175 40.344 0.000

X2 6.889 0.159 43.433 0.000

Means

X1 0.000 0.000 999.000 999.000

X2 0.000 0.000 999.000 999.000

X3 0.000 0.000 999.000 999.000

Intercepts

XQ1 3.932 0.048 82.505 0.000

XQ2 2.909 0.023 124.756 0.000

XQ3 0.405 0.007 58.241 0.000

XQ4 3.695 0.043 86.390 0.000

XQ5 3.180 0.033 97.645 0.000

XQ6 0.179 0.008 21.336 0.000

XQ7 4.747 0.038 123.549 0.000

XQ8 3.975 0.028 139.873 0.000

XQ9 0.111 0.006 17.162 0.000

Variances

X1 11.195 0.302 37.050 0.000

X2 8.110 0.225 35.975 0.000

X3 8.435 0.198 42.587 0.000

Residual Variances

XQ1 9.025 0.190 47.532 0.000

XQ2 2.046 0.045 45.592 0.000

XQ3 0.218 0.004 51.990 0.000

XQ4 8.173 0.146 56.043 0.000

XQ5 2.514 0.068 36.872 0.000

XQ6 0.311 0.006 56.030 0.000

XQ7 4.707 0.102 46.192 0.000

XQ8 2.165 0.054 40.418 0.000

XQ9 0.216 0.004 59.097 0.000

Group MALE

X1 BY

XQ1 1.000 0.000 999.000 999.000

XQ2 0.542 0.010 53.099 0.000

XQ3 0.150 0.003 53.259 0.000

X2 BY

XQ4 1.000 0.000 999.000 999.000

XQ5 0.870 0.016 53.081 0.000

XQ6 0.185 0.004 48.368 0.000

X3 BY

XQ7 1.000 0.000 999.000 999.000

XQ8 0.797 0.012 66.720 0.000

XQ9 0.138 0.003 53.709 0.000

X2 WITH

X1 5.337 0.167 32.053 0.000

X3 WITH

X1 5.162 0.156 33.069 0.000

X2 5.584 0.156 35.862 0.000

Means

X1 0.000 0.000 999.000 999.000

X2 0.000 0.000 999.000 999.000

X3 0.000 0.000 999.000 999.000

Intercepts

XQ1 5.803 0.048 121.901 0.000

XQ2 3.041 0.024 125.850 0.000

XQ3 0.599 0.007 82.216 0.000

XQ4 4.930 0.047 105.946 0.000

XQ5 3.639 0.032 112.299 0.000

XQ6 0.408 0.008 49.159 0.000

XQ7 5.902 0.041 145.488 0.000

XQ8 4.206 0.030 141.396 0.000

XQ9 0.203 0.007 29.200 0.000

Variances

X1 9.341 0.285 32.799 0.000

X2 7.237 0.246 29.457 0.000

X3 7.908 0.210 37.682 0.000

Residual Variances

XQ1 8.499 0.192 44.206 0.000

XQ2 1.847 0.048 38.436 0.000

XQ3 0.209 0.004 47.098 0.000

XQ4 9.809 0.188 52.115 0.000

XQ5 2.786 0.081 34.393 0.000

XQ6 0.295 0.006 50.765 0.000

XQ7 5.049 0.120 42.150 0.000

XQ8 1.945 0.062 31.383 0.000

XQ9 0.232 0.004 55.205 0.000

MODEL RESULTS FOR THE METRIC MODEL

Two-Tailed

Estimate S.E. Est./S.E. P-Value

Group FEMALE

X1 BY

XQ1 1.000 0.000 999.000 999.000

XQ2 0.518 0.006 80.162 0.000

XQ3 0.143 0.002 78.818 0.000

X2 BY

XQ4 1.000 0.000 999.000 999.000

XQ5 0.905 0.010 87.433 0.000

XQ6 0.193 0.002 77.454 0.000

X3 BY

XQ7 1.000 0.000 999.000 999.000

XQ8 0.783 0.008 102.557 0.000

XQ9 0.137 0.002 80.959 0.000

X2 WITH

X1 7.192 0.170 42.402 0.000

X3 WITH

X1 6.801 0.159 42.665 0.000

X2 6.954 0.149 46.698 0.000

Means

X1 0.000 0.000 999.000 999.000

X2 0.000 0.000 999.000 999.000

X3 0.000 0.000 999.000 999.000

Intercepts

XQ1 3.932 0.047 83.228 0.000

XQ2 2.909 0.023 124.248 0.000

XQ3 0.405 0.007 57.975 0.000

XQ4 3.695 0.043 85.749 0.000

XQ5 3.180 0.032 97.869 0.000

XQ6 0.179 0.008 21.405 0.000

XQ7 4.747 0.038 123.974 0.000

XQ8 3.975 0.028 139.507 0.000

XQ9 0.111 0.006 17.156 0.000

Variances

X1 10.660 0.260 41.002 0.000

X2 8.394 0.206 40.826 0.000

X3 8.302 0.179 46.441 0.000

Residual Variances

XQ1 9.209 0.184 50.046 0.000

XQ2 2.013 0.043 46.626 0.000

XQ3 0.216 0.004 52.689 0.000

XQ4 8.134 0.144 56.369 0.000

XQ5 2.529 0.066 38.160 0.000

XQ6 0.312 0.006 56.654 0.000

XQ7 4.750 0.099 47.903 0.000

XQ8 2.142 0.052 41.435 0.000

XQ9 0.216 0.004 59.513 0.000

Group MALE

X1 BY

XQ1 1.000 0.000 999.000 999.000

XQ2 0.518 0.006 80.162 0.000

XQ3 0.143 0.002 78.818 0.000

X2 BY

XQ4 1.000 0.000 999.000 999.000

XQ5 0.905 0.010 87.433 0.000

XQ6 0.193 0.002 77.454 0.000

X3 BY

XQ7 1.000 0.000 999.000 999.000

XQ8 0.783 0.008 102.557 0.000

XQ9 0.137 0.002 80.959 0.000

X2 WITH

X1 5.330 0.147 36.257 0.000

X3 WITH

X1 5.386 0.149 36.104 0.000

X2 5.466 0.134 40.706 0.000

Means

X1 0.000 0.000 999.000 999.000

X2 0.000 0.000 999.000 999.000

X3 0.000 0.000 999.000 999.000

Intercepts

XQ1 5.803 0.048 120.702 0.000

XQ2 3.041 0.024 126.430 0.000

XQ3 0.599 0.007 82.650 0.000

XQ4 4.930 0.046 107.010 0.000

XQ5 3.639 0.033 111.939 0.000

XQ6 0.408 0.008 48.965 0.000

XQ7 5.902 0.041 144.891 0.000

XQ8 4.206 0.030 141.798 0.000

XQ9 0.203 0.007 29.213 0.000

Variances

X1 9.907 0.248 39.997 0.000

X2 6.797 0.183 37.198 0.000

X3 8.076 0.183 44.190 0.000

Residual Variances

XQ1 8.289 0.183 45.363 0.000

XQ2 1.890 0.045 41.970 0.000

XQ3 0.211 0.004 48.964 0.000

XQ4 9.911 0.184 53.973 0.000

XQ5 2.759 0.076 36.434 0.000

XQ6 0.293 0.006 51.876 0.000

XQ7 4.988 0.113 44.104 0.000

XQ8 1.980 0.057 34.822 0.000

XQ9 0.231 0.004 55.893 0.000

MODEL RESULTS FOR THE SCALAR MODEL

Two-Tailed

Estimate S.E. Est./S.E. P-Value

Group FEMALE

X1 BY

XQ1 1.000 0.000 999.000 999.000

XQ2 0.479 0.006 79.619 0.000

XQ3 0.140 0.002 80.621 0.000

X2 BY

XQ4 1.000 0.000 999.000 999.000

XQ5 0.874 0.010 88.647 0.000

XQ6 0.193 0.002 79.411 0.000

X3 BY

XQ7 1.000 0.000 999.000 999.000

XQ8 0.758 0.007 102.037 0.000

XQ9 0.135 0.002 81.632 0.000

X2 WITH

X1 7.619 0.176 43.208 0.000

X3 WITH

X1 7.177 0.166 43.246 0.000

X2 7.207 0.152 47.275 0.000

Means

X1 0.000 0.000 999.000 999.000

X2 0.000 0.000 999.000 999.000

X3 0.000 0.000 999.000 999.000

Intercepts

XQ1 4.270 0.045 94.048 0.000

XQ2 2.699 0.022 124.208 0.000

XQ3 0.419 0.006 64.745 0.000

XQ4 3.860 0.040 96.318 0.000

XQ5 3.056 0.031 97.218 0.000

XQ6 0.212 0.008 27.510 0.000

XQ7 4.964 0.037 134.241 0.000

XQ8 3.837 0.027 140.260 0.000

XQ9 0.111 0.006 19.470 0.000

Variances

X1 11.391 0.271 42.080 0.000

X2 8.672 0.209 41.507 0.000

X3 8.557 0.183 46.864 0.000

Residual Variances

XQ1 9.045 0.188 48.027 0.000

XQ2 2.175 0.045 48.516 0.000

XQ3 0.216 0.004 52.058 0.000

XQ4 8.074 0.145 55.733 0.000

XQ5 2.661 0.067 39.721 0.000

XQ6 0.311 0.006 56.083 0.000

XQ7 4.713 0.101 46.599 0.000

XQ8 2.246 0.053 42.574 0.000

XQ9 0.215 0.004 59.353 0.000

Group MALE

X1 BY

XQ1 1.000 0.000 999.000 999.000

XQ2 0.479 0.006 79.619 0.000

XQ3 0.140 0.002 80.621 0.000

X2 BY

XQ4 1.000 0.000 999.000 999.000

XQ5 0.874 0.010 88.647 0.000

XQ6 0.193 0.002 79.411 0.000

X3 BY

XQ7 1.000 0.000 999.000 999.000

XQ8 0.758 0.007 102.037 0.000

XQ9 0.135 0.002 81.632 0.000

X2 WITH

X1 5.617 0.153 36.623 0.000

X3 WITH

X1 5.659 0.156 36.335 0.000

X2 5.662 0.138 41.023 0.000

Means

X1 1.188 0.060 19.719 0.000

X2 0.841 0.049 17.051 0.000

X3 0.679 0.050 13.486 0.000

Intercepts

XQ1 4.270 0.045 94.048 0.000

XQ2 2.699 0.022 124.208 0.000

XQ3 0.419 0.006 64.745 0.000

XQ4 3.860 0.040 96.318 0.000

XQ5 3.056 0.031 97.218 0.000

XQ6 0.212 0.008 27.510 0.000

XQ7 4.964 0.037 134.241 0.000

XQ8 3.837 0.027 140.260 0.000

XQ9 0.111 0.006 19.470 0.000

Variances

X1 10.543 0.258 40.901 0.000

X2 7.011 0.187 37.560 0.000

X3 8.317 0.187 44.442 0.000

Residual Variances

XQ1 8.129 0.189 42.999 0.000

XQ2 2.080 0.047 44.181 0.000

XQ3 0.210 0.004 48.178 0.000

XQ4 9.869 0.185 53.282 0.000

XQ5 2.914 0.077 38.010 0.000

XQ6 0.292 0.006 51.156 0.000

XQ7 4.962 0.116 42.609 0.000

XQ8 2.107 0.058 36.228 0.000

XQ9 0.231 0.004 55.687 0.000

QUALITY OF NUMERICAL RESULTS FOR THE CONFIGURAL MODEL

Condition Number for the Information Matrix 0.233E-03

(ratio of smallest to largest eigenvalue)

QUALITY OF NUMERICAL RESULTS FOR THE METRIC MODEL

Condition Number for the Information Matrix 0.156E-03

(ratio of smallest to largest eigenvalue)

QUALITY OF NUMERICAL RESULTS FOR THE SCALAR MODEL

Condition Number for the Information Matrix 0.141E-03

(ratio of smallest to largest eigenvalue)

Tests of Measurement Invariance.

Depressive symptoms scale (2015-2020)

TITLE: SEM-jchg

INPUT INSTRUCTIONS

TITLE: SEM-jchg

Data:

file is CLPMC1.dat;

FORMAT IS FREE;

TYPE IS INDIVIDUAL;

VARIABLE:

Names = id1 am1-am3 xq1-xq9 yq1-yq30 sex age1-age3

cx1-cx3 edu1-edu3 mar1-mar3 smok1-smok3;

USEvar = yq1-yq30;

Grouping=sex(1=male 0=female);

model:

y1 by yq1-yq10;

y2 by yq11-yq20;

y3 by yq21-yq30;

analysis: estimator=ML;

model=configural metric scalar;

Output: standardized;

MODEL FIT INFORMATION

Invariance Testing

Number of Degrees of

Model Parameters Chi-Square Freedom P-Value

Configural 186 23966.023 804 0.0000

Metric 159 24311.925 831 0.0000

Scalar 132 25186.834 858 0.0000

Degrees of

Models Compared Chi-Square Freedom P-Value

Metric against Configural 345.902 27 0.0000

Scalar against Configural 1220.811 54 0.0000

Scalar against Metric 874.909 27 0.0000

MODEL FIT INFORMATION FOR THE CONFIGURAL MODEL

Number of Free Parameters 186

Loglikelihood

H0 Value -688439.321

H1 Value -676456.310

Information Criteria

Akaike (AIC) 1377250.642

Bayesian (BIC) 1378687.995

Sample-Size Adjusted BIC 1378096.899

(n* = (n + 2) / 24)

Chi-Square Test of Model Fit

Value 23966.023

Degrees of Freedom 804

P-Value 0.0000

Chi-Square Contribution and P-Value From Each Group (degrees of freedom = 402)

FEMALE 12737.087 0.000

MALE 11228.936 0.000

RMSEA (Root Mean Square Error Of Approximation)

Estimate 0.059

90 Percent C.I. 0.058 0.059

Probability RMSEA <= .05 0.000

CFI/TLI

CFI 0.886

TLI 0.876

Chi-Square Test of Model Fit for the Baseline Model

Value 203415.190

Degrees of Freedom 870

P-Value 0.0000

SRMR (Standardized Root Mean Square Residual)

Value 0.041

MODEL FIT INFORMATION FOR THE METRIC MODEL

Number of Free Parameters 159

Loglikelihood

H0 Value -688612.272

H1 Value -676456.310

Information Criteria

Akaike (AIC) 1377542.545

Bayesian (BIC) 1378771.250

Sample-Size Adjusted BIC 1378265.958

(n* = (n + 2) / 24)

Chi-Square Test of Model Fit

Value 24311.925

Degrees of Freedom 831

P-Value 0.0000

Chi-Square Contribution From Each Group

FEMALE 12908.492

MALE 11403.434

RMSEA (Root Mean Square Error Of Approximation)

Estimate 0.058

90 Percent C.I. 0.057 0.059

Probability RMSEA <= .05 0.000

CFI/TLI

CFI 0.884

TLI 0.879

Chi-Square Test of Model Fit for the Baseline Model

Value 203415.190

Degrees of Freedom 870

P-Value 0.0000

SRMR (Standardized Root Mean Square Residual)

Value 0.043

MODEL FIT INFORMATION FOR THE SCALAR MODEL

Number of Free Parameters 132

Loglikelihood

H0 Value -689049.727

H1 Value -676456.310

Information Criteria

Akaike (AIC) 1378363.453

Bayesian (BIC) 1379383.510

Sample-Size Adjusted BIC 1378964.023

(n* = (n + 2) / 24)

Chi-Square Test of Model Fit

Value 25186.834

Degrees of Freedom 858

P-Value 0.0000

Chi-Square Contribution From Each Group

FEMALE 13308.322

MALE 11878.512

RMSEA (Root Mean Square Error Of Approximation)

Estimate 0.058

90 Percent C.I. 0.058 0.059

Probability RMSEA <= .05 0.000

CFI/TLI

CFI 0.880

TLI 0.878

Chi-Square Test of Model Fit for the Baseline Model

Value 203415.190

Degrees of Freedom 870

P-Value 0.0000

SRMR (Standardized Root Mean Square Residual)

Value 0.044

MODEL RESULTS FOR THE CONFIGURAL MODEL

Two-Tailed

Estimate S.E. Est./S.E. P-Value

Group FEMALE

Y1 BY

YQ1 1.000 0.000 999.000 999.000

YQ2 0.915 0.015 62.229 0.000

YQ3 1.051 0.014 72.458 0.000

YQ4 0.984 0.015 65.011 0.000

YQ5 0.607 0.018 34.298 0.000

YQ6 0.617 0.011 54.617 0.000

YQ7 0.842 0.016 52.010 0.000

YQ8 0.826 0.016 51.957 0.000

YQ9 0.744 0.013 56.933 0.000

YQ10 0.662 0.011 58.109 0.000

Y2 BY

YQ11 1.000 0.000 999.000 999.000

YQ12 0.940 0.015 61.716 0.000

YQ13 1.064 0.015 71.290 0.000

YQ14 1.034 0.016 64.869 0.000

YQ15 0.560 0.018 31.881 0.000

YQ16 0.694 0.012 56.784 0.000

YQ17 0.864 0.017 52.163 0.000

YQ18 0.811 0.016 51.396 0.000

YQ19 0.800 0.014 56.755 0.000

YQ20 0.716 0.013 56.879 0.000

Y3 BY

YQ21 1.000 0.000 999.000 999.000

YQ22 0.980 0.015 65.274 0.000

YQ23 1.045 0.015 71.664 0.000

YQ24 1.054 0.015 68.678 0.000

YQ25 0.435 0.017 25.329 0.000

YQ26 0.723 0.012 58.888 0.000

YQ27 0.895 0.016 56.267 0.000

YQ28 0.668 0.015 43.410 0.000

YQ29 0.799 0.013 59.312 0.000

YQ30 0.750 0.013 59.681 0.000

Y2 WITH

Y1 0.396 0.012 32.685 0.000

Y3 WITH

Y1 0.361 0.012 30.361 0.000

Y2 0.467 0.013 36.091 0.000

Means

Y1 0.000 0.000 999.000 999.000

Y2 0.000 0.000 999.000 999.000

Y3 0.000 0.000 999.000 999.000

Intercepts

YQ1 0.843 0.013 63.723 0.000

YQ2 0.742 0.013 57.207 0.000

YQ3 0.800 0.013 62.191 0.000

YQ4 0.761 0.013 57.680 0.000

YQ5 1.111 0.015 72.814 0.000

YQ6 0.298 0.010 30.438 0.000

YQ7 1.029 0.014 72.976 0.000

YQ8 0.954 0.014 69.030 0.000

YQ9 0.442 0.011 39.147 0.000

YQ10 0.276 0.010 28.060 0.000

YQ11 0.893 0.013 67.345 0.000

YQ12 0.812 0.013 61.089 0.000

YQ13 0.867 0.013 66.271 0.000

YQ14 0.904 0.014 65.801 0.000

YQ15 1.036 0.015 68.918 0.000

YQ16 0.391 0.010 37.438 0.000

YQ17 1.150 0.014 80.768 0.000

YQ18 0.934 0.014 68.749 0.000

YQ19 0.537 0.012 44.729 0.000

YQ20 0.362 0.011 33.753 0.000

YQ21 0.953 0.013 71.110 0.000

YQ22 0.901 0.014 66.619 0.000

YQ23 0.843 0.013 64.033 0.000

YQ24 0.887 0.014 64.988 0.000

YQ25 1.100 0.015 73.134 0.000

YQ26 0.430 0.011 39.669 0.000

YQ27 1.131 0.014 80.232 0.000

YQ28 0.950 0.014 70.112 0.000

YQ29 0.524 0.012 44.344 0.000

YQ30 0.400 0.011 36.303 0.000

Variances

Y1 0.834 0.022 38.427 0.000

Y2 0.823 0.022 38.016 0.000

Y3 0.855 0.022 38.524 0.000

Residual Variances

YQ1 0.726 0.013 57.224 0.000

YQ2 0.799 0.013 59.548 0.000

YQ3 0.553 0.010 52.972 0.000

YQ4 0.741 0.013 57.819 0.000

YQ5 1.765 0.027 65.246 0.000

YQ6 0.537 0.009 61.823 0.000

YQ7 1.178 0.019 62.638 0.000

YQ8 1.131 0.018 62.504 0.000

YQ9 0.675 0.011 60.983 0.000

YQ10 0.497 0.008 60.531 0.000

YQ11 0.742 0.013 57.602 0.000

YQ12 0.844 0.014 59.671 0.000

YQ13 0.593 0.011 53.728 0.000

YQ14 0.800 0.014 57.883 0.000

YQ15 1.752 0.027 65.511 0.000

YQ16 0.575 0.009 61.037 0.000

YQ17 1.192 0.019 62.577 0.000

YQ18 1.100 0.018 62.676 0.000

YQ19 0.756 0.012 60.853 0.000

YQ20 0.604 0.010 60.835 0.000

YQ21 0.746 0.013 57.748 0.000

YQ22 0.808 0.014 58.720 0.000

YQ23 0.611 0.011 54.713 0.000

YQ24 0.709 0.013 56.475 0.000

YQ25 1.853 0.028 66.031 0.000

YQ26 0.599 0.010 60.891 0.000

YQ27 1.084 0.018 61.887 0.000

YQ28 1.253 0.019 64.321 0.000

YQ29 0.699 0.012 60.432 0.000

YQ30 0.603 0.010 60.328 0.000

Group MALE

Y1 BY

YQ1 1.000 0.000 999.000 999.000

YQ2 0.964 0.018 53.852 0.000

YQ3 1.071 0.017 62.025 0.000

YQ4 1.089 0.019 58.340 0.000

YQ5 0.753 0.023 32.397 0.000

YQ6 0.554 0.011 49.875 0.000

YQ7 0.870 0.020 44.584 0.000

YQ8 0.890 0.021 42.761 0.000

YQ9 0.827 0.015 54.050 0.000

YQ10 0.652 0.012 52.726 0.000

Y2 BY

YQ11 1.000 0.000 999.000 999.000

YQ12 1.026 0.019 54.614 0.000

YQ13 1.114 0.018 60.725 0.000

YQ14 1.127 0.020 55.968 0.000

YQ15 0.666 0.023 28.488 0.000

YQ16 0.609 0.013 48.463 0.000

YQ17 0.919 0.021 44.679 0.000

YQ18 0.856 0.021 40.835 0.000

YQ19 0.881 0.017 52.556 0.000

YQ20 0.735 0.014 51.050 0.000

Y3 BY

YQ21 1.000 0.000 999.000 999.000

YQ22 1.032 0.017 59.038 0.000

YQ23 1.109 0.017 66.070 0.000

YQ24 1.118 0.018 60.647 0.000

YQ25 0.585 0.021 27.346 0.000

YQ26 0.637 0.012 53.565 0.000

YQ27 0.927 0.018 50.222 0.000

YQ28 0.768 0.019 39.997 0.000

YQ29 0.859 0.015 56.447 0.000

YQ30 0.786 0.014 57.408 0.000

Y2 WITH

Y1 0.239 0.009 27.784 0.000

Y3 WITH

Y1 0.239 0.009 27.022 0.000

Y2 0.297 0.010 31.292 0.000

Means

Y1 0.000 0.000 999.000 999.000

Y2 0.000 0.000 999.000 999.000

Y3 0.000 0.000 999.000 999.000

Intercepts

YQ1 0.546 0.012 44.895 0.000

YQ2 0.535 0.012 43.639 0.000

YQ3 0.530 0.012 44.953 0.000

YQ4 0.554 0.013 43.933 0.000

YQ5 1.090 0.016 67.871 0.000

YQ6 0.092 0.008 12.282 0.000

YQ7 0.655 0.013 48.870 0.000

YQ8 0.827 0.014 57.764 0.000

YQ9 0.282 0.010 27.323 0.000

YQ10 0.128 0.008 15.438 0.000

YQ11 0.592 0.012 48.447 0.000

YQ12 0.606 0.013 48.436 0.000

YQ13 0.601 0.012 49.698 0.000

YQ14 0.669 0.013 50.945 0.000

YQ15 1.049 0.016 66.042 0.000

YQ16 0.154 0.008 18.674 0.000

YQ17 0.760 0.014 55.359 0.000

YQ18 0.818 0.014 58.106 0.000

YQ19 0.367 0.011 33.614 0.000

YQ20 0.207 0.009 22.103 0.000

YQ21 0.614 0.013 48.987 0.000

YQ22 0.658 0.013 50.883 0.000

YQ23 0.578 0.012 46.737 0.000

YQ24 0.675 0.013 50.075 0.000

YQ25 1.073 0.016 67.847 0.000

YQ26 0.163 0.009 18.749 0.000

YQ27 0.710 0.014 52.229 0.000

YQ28 0.811 0.014 57.202 0.000

YQ29 0.352 0.011 31.788 0.000

YQ30 0.231 0.010 23.239 0.000

Variances

Y1 0.560 0.017 33.486 0.000

Y2 0.544 0.017 32.628 0.000

Y3 0.618 0.018 34.621 0.000

Residual Variances

YQ1 0.605 0.011 55.046 0.000

YQ2 0.663 0.012 56.293 0.000

YQ3 0.451 0.009 50.855 0.000

YQ4 0.590 0.011 53.507 0.000

YQ5 1.714 0.028 61.170 0.000

YQ6 0.274 0.005 57.464 0.000

YQ7 0.990 0.017 59.242 0.000

YQ8 1.171 0.020 59.554 0.000

YQ9 0.455 0.008 55.685 0.000

YQ10 0.306 0.005 56.114 0.000

YQ11 0.630 0.011 55.404 0.000

YQ12 0.659 0.012 55.444 0.000

YQ13 0.475 0.009 50.717 0.000

YQ14 0.669 0.012 54.209 0.000

YQ15 1.745 0.028 61.557 0.000

YQ16 0.337 0.006 57.711 0.000

YQ17 1.026 0.017 59.046 0.000

YQ18 1.160 0.019 59.814 0.000

YQ19 0.516 0.009 55.749 0.000

YQ20 0.398 0.007 56.361 0.000

YQ21 0.620 0.011 55.483 0.000

YQ22 0.658 0.012 55.428 0.000

YQ23 0.445 0.009 50.191 0.000

YQ24 0.658 0.012 54.269 0.000

YQ25 1.758 0.028 61.841 0.000

YQ26 0.346 0.006 57.474 0.000

YQ27 0.924 0.016 58.627 0.000

YQ28 1.217 0.020 60.522 0.000

YQ29 0.508 0.009 55.967 0.000

YQ30 0.395 0.007 55.399 0.000

MODEL RESULTS FOR THE METRIC MODEL

Two-Tailed

Estimate S.E. Est./S.E. P-Value

Group FEMALE

Y1 BY

YQ1 1.000 0.000 999.000 999.000

YQ2 0.936 0.011 82.261 0.000

YQ3 1.059 0.011 95.294 0.000

YQ4 1.029 0.012 87.306 0.000

YQ5 0.665 0.014 46.988 0.000

YQ6 0.580 0.008 73.680 0.000

YQ7 0.855 0.012 68.499 0.000

YQ8 0.852 0.013 67.256 0.000

YQ9 0.782 0.010 78.706 0.000

YQ10 0.655 0.008 78.521 0.000

Y2 BY

YQ11 1.000 0.000 999.000 999.000

YQ12 0.977 0.012 82.496 0.000

YQ13 1.085 0.012 93.666 0.000

YQ14 1.073 0.013 85.669 0.000

YQ15 0.601 0.014 42.669 0.000

YQ16 0.647 0.009 74.081 0.000

YQ17 0.887 0.013 68.670 0.000

YQ18 0.829 0.013 65.594 0.000

YQ19 0.835 0.011 77.563 0.000

YQ20 0.724 0.009 76.557 0.000

Y3 BY

YQ21 1.000 0.000 999.000 999.000

YQ22 1.003 0.011 88.031 0.000

YQ23 1.074 0.011 97.582 0.000

YQ24 1.082 0.012 91.555 0.000

YQ25 0.498 0.013 36.989 0.000

YQ26 0.673 0.009 79.052 0.000

YQ27 0.909 0.012 75.392 0.000

YQ28 0.709 0.012 58.887 0.000

YQ29 0.826 0.010 81.969 0.000

YQ30 0.766 0.009 83.001 0.000

Y2 WITH

Y1 0.383 0.011 34.178 0.000

Y3 WITH

Y1 0.349 0.011 31.593 0.000

Y2 0.451 0.012 38.209 0.000

Means

Y1 0.000 0.000 999.000 999.000

Y2 0.000 0.000 999.000 999.000

Y3 0.000 0.000 999.000 999.000

Intercepts

YQ1 0.843 0.013 64.160 0.000

YQ2 0.742 0.013 57.022 0.000

YQ3 0.800 0.013 62.435 0.000

YQ4 0.761 0.013 56.909 0.000

YQ5 1.111 0.015 72.040 0.000

YQ6 0.298 0.010 31.146 0.000

YQ7 1.029 0.014 72.995 0.000

YQ8 0.954 0.014 68.765 0.000

YQ9 0.442 0.011 38.630 0.000

YQ10 0.276 0.010 28.318 0.000

YQ11 0.893 0.013 67.901 0.000

YQ12 0.812 0.013 60.574 0.000

YQ13 0.867 0.013 66.228 0.000

YQ14 0.904 0.014 65.239 0.000

YQ15 1.036 0.015 68.480 0.000

YQ16 0.391 0.010 38.504 0.000

YQ17 1.150 0.014 80.553 0.000

YQ18 0.934 0.014 68.689 0.000

YQ19 0.537 0.012 44.267 0.000

YQ20 0.362 0.011 33.834 0.000

YQ21 0.953 0.013 71.687 0.000

YQ22 0.901 0.014 66.431 0.000

YQ23 0.843 0.013 63.695 0.000

YQ24 0.887 0.014 64.710 0.000

YQ25 1.100 0.015 72.446 0.000

YQ26 0.430 0.011 40.871 0.000

YQ27 1.131 0.014 80.262 0.000

YQ28 0.950 0.014 69.499 0.000

YQ29 0.524 0.012 44.040 0.000

YQ30 0.400 0.011 36.229 0.000

Variances

Y1 0.808 0.018 43.983 0.000

Y2 0.794 0.018 43.440 0.000

Y3 0.826 0.019 44.485 0.000

Residual Variances

YQ1 0.730 0.013 57.880 0.000

YQ2 0.798 0.013 59.836 0.000

YQ3 0.555 0.010 53.775 0.000

YQ4 0.736 0.013 57.894 0.000

YQ5 1.760 0.027 65.181 0.000

YQ6 0.544 0.009 62.495 0.000

YQ7 1.178 0.019 62.827 0.000

YQ8 1.126 0.018 62.615 0.000

YQ9 0.673 0.011 61.075 0.000

YQ10 0.499 0.008 61.072 0.000

YQ11 0.745 0.013 58.241 0.000

YQ12 0.841 0.014 59.860 0.000

YQ13 0.591 0.011 54.346 0.000

YQ14 0.795 0.014 58.064 0.000

YQ15 1.749 0.027 65.494 0.000

YQ16 0.586 0.009 61.838 0.000

YQ17 1.191 0.019 62.731 0.000

YQ18 1.099 0.017 62.833 0.000

YQ19 0.755 0.012 61.043 0.000

YQ20 0.605 0.010 61.261 0.000

YQ21 0.749 0.013 58.393 0.000

YQ22 0.807 0.014 59.092 0.000

YQ23 0.608 0.011 55.177 0.000

YQ24 0.707 0.012 56.808 0.000

YQ25 1.849 0.028 65.944 0.000

YQ26 0.611 0.010 61.737 0.000

YQ27 1.085 0.017 62.123 0.000

YQ28 1.248 0.019 64.316 0.000

YQ29 0.698 0.011 60.694 0.000

YQ30 0.603 0.010 60.693 0.000

Group MALE

Y1 BY

YQ1 1.000 0.000 999.000 999.000

YQ2 0.936 0.011 82.261 0.000

YQ3 1.059 0.011 95.294 0.000

YQ4 1.029 0.012 87.306 0.000

YQ5 0.665 0.014 46.988 0.000

YQ6 0.580 0.008 73.680 0.000

YQ7 0.855 0.012 68.499 0.000

YQ8 0.852 0.013 67.256 0.000

YQ9 0.782 0.010 78.706 0.000

YQ10 0.655 0.008 78.521 0.000

Y2 BY

YQ11 1.000 0.000 999.000 999.000

YQ12 0.977 0.012 82.496 0.000

YQ13 1.085 0.012 93.666 0.000

YQ14 1.073 0.013 85.669 0.000

YQ15 0.601 0.014 42.669 0.000

YQ16 0.647 0.009 74.081 0.000

YQ17 0.887 0.013 68.670 0.000

YQ18 0.829 0.013 65.594 0.000

YQ19 0.835 0.011 77.563 0.000

YQ20 0.724 0.009 76.557 0.000

Y3 BY

YQ21 1.000 0.000 999.000 999.000

YQ22 1.003 0.011 88.031 0.000

YQ23 1.074 0.011 97.582 0.000

YQ24 1.082 0.012 91.555 0.000

YQ25 0.498 0.013 36.989 0.000

YQ26 0.673 0.009 79.052 0.000

YQ27 0.909 0.012 75.392 0.000

YQ28 0.709 0.012 58.887 0.000

YQ29 0.826 0.010 81.969 0.000

YQ30 0.766 0.009 83.001 0.000

Y2 WITH

Y1 0.249 0.008 29.774 0.000

Y3 WITH

Y1 0.249 0.009 28.628 0.000

Y2 0.311 0.009 34.068 0.000

Means

Y1 0.000 0.000 999.000 999.000

Y2 0.000 0.000 999.000 999.000

Y3 0.000 0.000 999.000 999.000

Intercepts

YQ1 0.546 0.012 44.519 0.000

YQ2 0.535 0.012 43.809 0.000

YQ3 0.530 0.012 44.737 0.000

YQ4 0.554 0.012 44.630 0.000

YQ5 1.090 0.016 68.715 0.000

YQ6 0.092 0.008 11.994 0.000

YQ7 0.655 0.013 48.854 0.000

YQ8 0.827 0.014 58.043 0.000

YQ9 0.282 0.010 27.717 0.000

YQ10 0.128 0.008 15.287 0.000

YQ11 0.592 0.012 47.953 0.000

YQ12 0.606 0.012 48.914 0.000

YQ13 0.601 0.012 49.737 0.000

YQ14 0.669 0.013 51.476 0.000

YQ15 1.049 0.016 66.532 0.000

YQ16 0.154 0.009 18.109 0.000

YQ17 0.760 0.014 55.536 0.000

YQ18 0.818 0.014 58.170 0.000

YQ19 0.367 0.011 33.995 0.000

YQ20 0.207 0.009 22.045 0.000

YQ21 0.614 0.013 48.522 0.000

YQ22 0.658 0.013 51.049 0.000

YQ23 0.578 0.012 47.004 0.000

YQ24 0.675 0.013 50.340 0.000

YQ25 1.073 0.016 68.575 0.000

YQ26 0.163 0.009 18.173 0.000

YQ27 0.710 0.014 52.207 0.000

YQ28 0.811 0.014 57.793 0.000

YQ29 0.352 0.011 32.025 0.000

YQ30 0.231 0.010 23.288 0.000

Variances

Y1 0.583 0.014 42.198 0.000

Y2 0.571 0.014 41.663 0.000

Y3 0.645 0.015 42.941 0.000

Residual Variances

YQ1 0.602 0.011 55.339 0.000

YQ2 0.664 0.012 56.793 0.000

YQ3 0.450 0.009 51.480 0.000

YQ4 0.598 0.011 54.550 0.000

YQ5 1.725 0.028 61.448 0.000

YQ6 0.271 0.005 57.280 0.000

YQ7 0.989 0.017 59.457 0.000

YQ8 1.176 0.020 59.924 0.000

YQ9 0.458 0.008 56.432 0.000

YQ10 0.304 0.005 56.320 0.000

YQ11 0.627 0.011 55.679 0.000

YQ12 0.663 0.012 56.151 0.000

YQ13 0.476 0.009 51.563 0.000

YQ14 0.674 0.012 55.043 0.000

YQ15 1.751 0.028 61.747 0.000

YQ16 0.334 0.006 57.361 0.000

YQ17 1.027 0.017 59.340 0.000

YQ18 1.163 0.019 60.102 0.000

YQ19 0.519 0.009 56.462 0.000

YQ20 0.397 0.007 56.726 0.000

YQ21 0.616 0.011 55.678 0.000

YQ22 0.659 0.012 55.905 0.000

YQ23 0.447 0.009 51.074 0.000

YQ24 0.660 0.012 54.890 0.000

YQ25 1.768 0.029 61.999 0.000

YQ26 0.342 0.006 57.119 0.000

YQ27 0.923 0.016 58.843 0.000

YQ28 1.226 0.020 60.829 0.000

YQ29 0.510 0.009 56.539 0.000

YQ30 0.395 0.007 55.891 0.000

MODEL RESULTS FOR THE SCALAR MODEL

Two-Tailed

Estimate S.E. Est./S.E. P-Value

Group FEMALE

Y1 BY

YQ1 1.000 0.000 999.000 999.000

YQ2 0.929 0.011 83.366 0.000

YQ3 1.055 0.011 96.845 0.000

YQ4 1.018 0.012 88.417 0.000

YQ5 0.646 0.014 46.624 0.000

YQ6 0.582 0.008 75.183 0.000

YQ7 0.866 0.012 70.237 0.000

YQ8 0.839 0.012 67.636 0.000

YQ9 0.774 0.010 79.674 0.000

YQ10 0.651 0.008 79.598 0.000

Y2 BY

YQ11 1.000 0.000 999.000 999.000

YQ12 0.968 0.012 83.585 0.000

YQ13 1.079 0.011 95.189 0.000

YQ14 1.064 0.012 86.897 0.000

YQ15 0.580 0.014 42.086 0.000

YQ16 0.651 0.009 75.682 0.000

YQ17 0.898 0.013 70.433 0.000

YQ18 0.815 0.012 65.927 0.000

YQ19 0.827 0.011 78.543 0.000

YQ20 0.717 0.009 77.579 0.000

Y3 BY

YQ21 1.000 0.000 999.000 999.000

YQ22 0.994 0.011 89.408 0.000

YQ23 1.064 0.011 99.222 0.000

YQ24 1.066 0.011 92.750 0.000

YQ25 0.483 0.013 36.733 0.000

YQ26 0.677 0.008 80.875 0.000

YQ27 0.919 0.012 77.400 0.000

YQ28 0.698 0.012 59.406 0.000

YQ29 0.815 0.010 83.049 0.000

YQ30 0.757 0.009 84.173 0.000

Y2 WITH

Y1 0.387 0.011 34.273 0.000

Y3 WITH

Y1 0.354 0.011 31.672 0.000

Y2 0.458 0.012 38.351 0.000

Means

Y1 0.000 0.000 999.000 999.000

Y2 0.000 0.000 999.000 999.000

Y3 0.000 0.000 999.000 999.000

Intercepts

YQ1 0.819 0.012 69.684 0.000

YQ2 0.755 0.011 66.583 0.000

YQ3 0.797 0.012 67.560 0.000

YQ4 0.786 0.012 65.989 0.000

YQ5 1.178 0.012 96.959 0.000

YQ6 0.260 0.008 34.340 0.000

YQ7 0.948 0.012 80.032 0.000

YQ8 0.992 0.012 84.465 0.000

YQ9 0.461 0.010 47.810 0.000

YQ10 0.285 0.008 35.184 0.000

YQ11 0.867 0.012 73.866 0.000

YQ12 0.831 0.012 71.319 0.000

YQ13 0.870 0.012 72.385 0.000

YQ14 0.921 0.012 74.368 0.000

YQ15 1.110 0.012 93.881 0.000

YQ16 0.347 0.008 41.822 0.000

YQ17 1.067 0.012 88.218 0.000

YQ18 0.974 0.011 84.816 0.000

YQ19 0.558 0.010 54.560 0.000

YQ20 0.377 0.009 42.139 0.000

YQ21 0.918 0.012 77.099 0.000

YQ22 0.915 0.012 76.400 0.000

YQ23 0.856 0.012 70.914 0.000

YQ24 0.924 0.012 74.314 0.000

YQ25 1.150 0.012 99.252 0.000

YQ26 0.379 0.009 43.774 0.000

YQ27 1.043 0.012 85.998 0.000

YQ28 0.974 0.011 87.471 0.000

YQ29 0.551 0.010 54.138 0.000

YQ30 0.421 0.009 44.899 0.000

Variances

Y1 0.817 0.018 44.379 0.000

Y2 0.803 0.018 43.852 0.000

Y3 0.838 0.019 44.943 0.000

Residual Variances

YQ1 0.729 0.013 57.770 0.000

YQ2 0.798 0.013 59.872 0.000

YQ3 0.554 0.010 53.757 0.000

YQ4 0.737 0.013 57.971 0.000

YQ5 1.767 0.027 65.164 0.000

YQ6 0.545 0.009 62.338 0.000

YQ7 1.183 0.019 62.531 0.000

YQ8 1.130 0.018 62.666 0.000

YQ9 0.674 0.011 61.119 0.000

YQ10 0.499 0.008 61.111 0.000

YQ11 0.744 0.013 58.132 0.000

YQ12 0.841 0.014 59.905 0.000

YQ13 0.591 0.011 54.355 0.000

YQ14 0.796 0.014 58.116 0.000

YQ15 1.757 0.027 65.451 0.000

YQ16 0.587 0.010 61.642 0.000

YQ17 1.197 0.019 62.419 0.000

YQ18 1.102 0.018 62.878 0.000

YQ19 0.756 0.012 61.095 0.000

YQ20 0.606 0.010 61.306 0.000

YQ21 0.748 0.013 58.219 0.000

YQ22 0.807 0.014 59.123 0.000

YQ23 0.609 0.011 55.225 0.000

YQ24 0.711 0.012 56.928 0.000

YQ25 1.852 0.028 65.932 0.000

YQ26 0.612 0.010 61.496 0.000

YQ27 1.091 0.018 61.743 0.000

YQ28 1.250 0.019 64.337 0.000

YQ29 0.699 0.012 60.753 0.000

YQ30 0.604 0.010 60.748 0.000

Group MALE

Y1 BY

YQ1 1.000 0.000 999.000 999.000

YQ2 0.929 0.011 83.366 0.000

YQ3 1.055 0.011 96.845 0.000

YQ4 1.018 0.012 88.417 0.000

YQ5 0.646 0.014 46.624 0.000

YQ6 0.582 0.008 75.183 0.000

YQ7 0.866 0.012 70.237 0.000

YQ8 0.839 0.012 67.636 0.000

YQ9 0.774 0.010 79.674 0.000

YQ10 0.651 0.008 79.598 0.000

Y2 BY

YQ11 1.000 0.000 999.000 999.000

YQ12 0.968 0.012 83.585 0.000

YQ13 1.079 0.011 95.189 0.000

YQ14 1.064 0.012 86.897 0.000

YQ15 0.580 0.014 42.086 0.000

YQ16 0.651 0.009 75.682 0.000

YQ17 0.898 0.013 70.433 0.000

YQ18 0.815 0.012 65.927 0.000

YQ19 0.827 0.011 78.543 0.000

YQ20 0.717 0.009 77.579 0.000

Y3 BY

YQ21 1.000 0.000 999.000 999.000

YQ22 0.994 0.011 89.408 0.000

YQ23 1.064 0.011 99.222 0.000

YQ24 1.066 0.011 92.750 0.000

YQ25 0.483 0.013 36.733 0.000

YQ26 0.677 0.008 80.875 0.000

YQ27 0.919 0.012 77.400 0.000

YQ28 0.698 0.012 59.406 0.000

YQ29 0.815 0.010 83.049 0.000

YQ30 0.757 0.009 84.173 0.000

Y2 WITH

Y1 0.251 0.008 29.826 0.000

Y3 WITH

Y1 0.252 0.009 28.677 0.000

Y2 0.314 0.009 34.155 0.000

Means

Y1 -0.251 0.014 -18.103 0.000

Y2 -0.252 0.014 -18.286 0.000

Y3 -0.271 0.014 -19.013 0.000

Intercepts

YQ1 0.819 0.012 69.684 0.000

YQ2 0.755 0.011 66.583 0.000

YQ3 0.797 0.012 67.560 0.000

YQ4 0.786 0.012 65.989 0.000

YQ5 1.178 0.012 96.959 0.000

YQ6 0.260 0.008 34.340 0.000

YQ7 0.948 0.012 80.032 0.000

YQ8 0.992 0.012 84.465 0.000

YQ9 0.461 0.010 47.810 0.000

YQ10 0.285 0.008 35.184 0.000

YQ11 0.867 0.012 73.866 0.000

YQ12 0.831 0.012 71.319 0.000

YQ13 0.870 0.012 72.385 0.000

YQ14 0.921 0.012 74.368 0.000

YQ15 1.110 0.012 93.881 0.000

YQ16 0.347 0.008 41.822 0.000

YQ17 1.067 0.012 88.218 0.000

YQ18 0.974 0.011 84.816 0.000

YQ19 0.558 0.010 54.560 0.000

YQ20 0.377 0.009 42.139 0.000

YQ21 0.918 0.012 77.099 0.000

YQ22 0.915 0.012 76.400 0.000

YQ23 0.856 0.012 70.914 0.000

YQ24 0.924 0.012 74.314 0.000

YQ25 1.150 0.012 99.252 0.000

YQ26 0.379 0.009 43.774 0.000

YQ27 1.043 0.012 85.998 0.000

YQ28 0.974 0.011 87.471 0.000

YQ29 0.551 0.010 54.138 0.000

YQ30 0.421 0.009 44.899 0.000

Variances

Y1 0.588 0.014 42.533 0.000

Y2 0.577 0.014 42.013 0.000

Y3 0.654 0.015 43.336 0.000

Residual Variances

YQ1 0.602 0.011 55.232 0.000

YQ2 0.665 0.012 56.826 0.000

YQ3 0.450 0.009 51.461 0.000

YQ4 0.600 0.011 54.636 0.000

YQ5 1.734 0.028 61.428 0.000

YQ6 0.271 0.005 57.113 0.000

YQ7 0.994 0.017 59.154 0.000

YQ8 1.180 0.020 59.956 0.000

YQ9 0.459 0.008 56.489 0.000

YQ10 0.304 0.005 56.349 0.000

YQ11 0.627 0.011 55.569 0.000

YQ12 0.664 0.012 56.200 0.000

YQ13 0.476 0.009 51.569 0.000

YQ14 0.675 0.012 55.098 0.000

YQ15 1.761 0.029 61.699 0.000

YQ16 0.334 0.006 57.155 0.000

YQ17 1.032 0.017 59.016 0.000

YQ18 1.167 0.019 60.128 0.000

YQ19 0.520 0.009 56.524 0.000

YQ20 0.398 0.007 56.769 0.000

YQ21 0.617 0.011 55.509 0.000

YQ22 0.659 0.012 55.933 0.000

YQ23 0.447 0.009 51.125 0.000

YQ24 0.663 0.012 54.981 0.000

YQ25 1.773 0.029 61.989 0.000

YQ26 0.343 0.006 56.873 0.000

YQ27 0.929 0.016 58.460 0.000

YQ28 1.228 0.020 60.850 0.000

YQ29 0.511 0.009 56.601 0.000

YQ30 0.396 0.007 55.949 0.000

QUALITY OF NUMERICAL RESULTS FOR THE CONFIGURAL MODEL

Condition Number for the Information Matrix 0.118E-01

(ratio of smallest to largest eigenvalue)

QUALITY OF NUMERICAL RESULTS FOR THE METRIC MODEL

Condition Number for the Information Matrix 0.151E-01

(ratio of smallest to largest eigenvalue)

QUALITY OF NUMERICAL RESULTS FOR THE SCALAR MODEL

Condition Number for the Information Matrix 0.122E-01

(ratio of smallest to largest eigenvalue)
