# Supplementary material for: Mediating Role of Internet Use in Cognitive-Depressive Pathways: A Random Intercept Cross-Lagged Panel Modeling Approach
Source: Int J Public Health. 2025 Oct 21;70:1608478. doi: 10.3389/ijph.2025.1608478 (PMC12583110; doi:10.3389/ijph.2025.1608478)
Supplement: Supplementary file 3 [file Supplementaryfile1.doc]

Appendix A

**Section 1**

In this study, the wave-3 (2015), wave-4(2018) and wave-5 (2020) data were used to explore the relationship between cognitive function, depressive symptoms.

Figure S1. Flow chart of participants into the analytical sample.


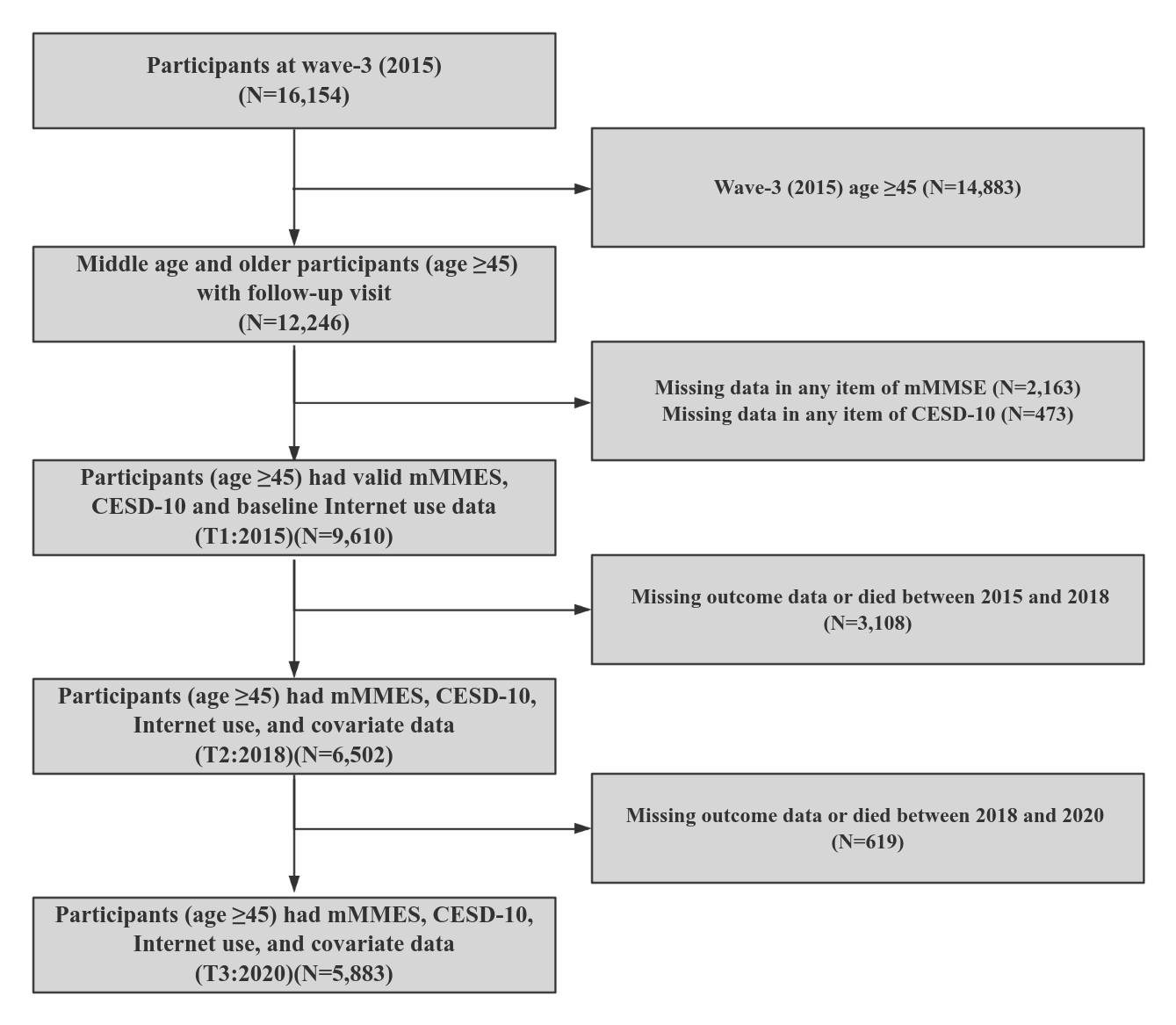


Note: Data were from the China Health and Retirement Longitudinal Study (http://charls.pku. edu.cn/en).

**Section 2**

The RI-CLPM was selected to explicitly separate between-person (stable trait-like differences) and within-person (temporal dynamics) effects in longitudinal data (Hamaker E, L., 2023)[[[1]](#footnote-2)]. Traditional CLPM conflates these effects by assuming all variability reflects causal processes, whereas RI-CLPM accounts for time-invariant individual differences through a random intercept. Key assumptions include:

1. Between-person level: Stable traits (e.g., baseline cognitive function) are captured by the random intercept.

2. Within-person level: Cross-lagged paths represent how deviations from one’s own mean (e.g., increased depressive symptoms at T1) predict subsequent deviations (e.g., cognitive decline at T2).

3. Stationarity: Within-person effects are consistent across time points.

Furthermore, using the RI-CLPM method, this study separates two distinct aspects of the link between internet use and health: between-person differences (whether prior internet users have better subsequent cognitive function and mental health) and within-person changes (Whether transitioning to internet use improves an individual’s cognitive abilities and mental health over time). This prevents the misattribution of inherent individual characteristics as causal effects of internet use.

Also, We prioritized RI-CLPM over alternative models for three reasons:

1. Traditional CLPM: Fails to distinguish within-person processes from between-person heterogeneity, potentially biasing estimates.

2. Latent Curve Models (LCM): Focus on trajectories rather than temporal dynamics, making them less suitable for testing reciprocal effects.

3.Growth Models were unsuitable as they cannot test reciprocal relationships, which were essential for isolating the within-person dynamics of our hypotheses.

4. Dynamic Structural Equation Modeling (DSEM): While flexible, DSEM requires intensive computational resources and larger samples; RI-CLPM offers a parsimonious balance for our research questions.

Figure S2. Descriptions of the RI-CLPM include the association between cognitive function, depressive symptoms, and Internet Use across the three time waves.


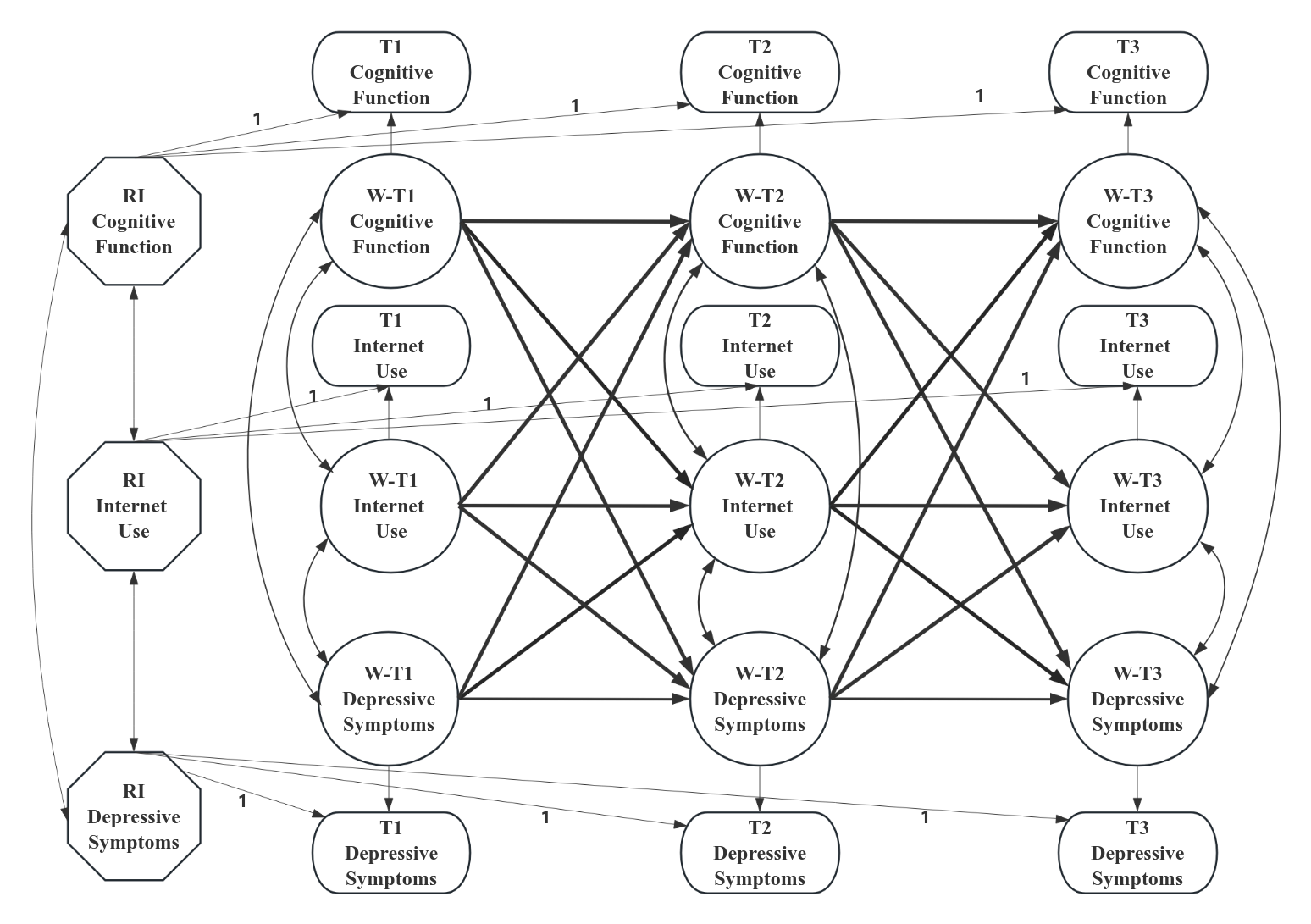


Note :W denotes within-person variable.

**Section 3**

Table S1. Measurement of the health status covariates.

| **Variables** | **Items/checklist** | **Scale** | **Score range** | **Cronbach’s** **α coefficients** |
| --- | --- | --- | --- | --- |
| Instrumental activities of daily living (IADL) | housework,  meal preparation, shopping, medication management, financial management, and telephone use. | Four-point scale  0= No, I do not have any difficulty;  1= I have difficulty, but I can still do it;  2= Yes, I have difficulty and need help;  3= I cannot do it | 0-18.  (Higher scores reflecting greater IADL dependence) | 0.765(2015), 0.820(2018) 0.876(2020) |
| Cumulative chronic disease index | hypertension, dyslipidemia, diabetes, cancer, chronic lung disease, liver disease, myocardial infarction, stroke, kidney disease, gastrointestinal disorders, psychiatric disorders, cognitive impairment, arthritis, and asthma. | Single terms scale  1= presence  0= absence | 0-14.   (Higher scores indicate more chronic conditions and a greater burden) | - |

Prior to RI-CLPM analysis, we assessed longitudinal measurement invariance for all latent variables. Nested model comparisons, using chi-square difference tests and fit indices (CFI, TLI, RMSEA), were employed to evaluate configural, metric, and scalar invariance. We considered measurement invariance to be supported if the change in CFI (ΔCFI) was ≤ 0.01 and the change in RMSEA (ΔRMSEA) was ≤ 0.015[[[2]](#footnote-3)]. These criteria were met for all measures, with scalar invariance confirmed for cognitive function and depressive symptom scales. Complete fit indices are reported in Following table, and the analysis code and results are available in Appendix C.

Table S2. Tests of Measurement Invariance .

| **Variable** | ***X^2^*** | ***df*** | **RMSEA[90%CI]** | **CFI** | **TLI** | △**RMSEA** | △ **CFI** | **P-value** |
| --- | --- | --- | --- | --- | --- | --- | --- | --- |
| Cognition function scale  (2015,2018,2020) |  |  |  |  |  |  |  |  |
| Model 0  (Configural Invariance level) | 3197.417 | 48 | 0.088 [0.086-0.091] | 0.951 | 0.928 |  |  | < 0.001 |
| Model 1  (Metric Invariance level) | 3222.501 | 54 | 0.084 [0.081-0.086] | 0.951 | 0.935 | 0.004 | < 0.001 | < 0.001 |
| Model 2  (Scalar Invariance level) | 4514.839 | 60 | 0.092 [0.090-0.094] | 0.941 | 0.924 | 0.008 | 0.01 | < 0.001 |
| Depressive symptoms scale  (2015,2018,2020) |  |  |  |  |  |  |  |  |
| Model 0  (Configural Invariance level) | 23966.023 | 804 | 0.059 [0.058-0.059] | 0.886 | 0.876 |  |  | < 0.001 |
| Model 1  (Metric Invariance level) | 24311.925 | 831 | 0.058 [0.057-0.059] | 0.884 | 0.879 | 0.001 | 0.002 | < 0.001 |
| Model 2  (Scalar Invariance level) | 25186.834 | 858 | 0.058 [0.058-0.059] | 0.880 | 0.878 | < 0.001 | 0.004 | < 0.001 |

Note: χ^2^ = Chi-square test statistic; df = degree of freedom; CFI = comparative fit index; TLI = Tucker Lewis index; RMSEA = root mean square error of approximation;

Table S3. Correlations between depressive symptoms and cognitive function and a manifest variable of internet use.

| **Variables** | **1** | **2** | **3** | **4** | **5** | **6** | **7** | **8** | **9** |
| --- | --- | --- | --- | --- | --- | --- | --- | --- | --- |
| 1. Cognitive Function 2015 | 1 |  |  |  |  |  |  |  |  |
| 1. Cognitive Function 2018 | 0.533^***^ | 1 |  |  |  |  |  |  |  |
| 1. Cognitive Function 2020 | 0.560^***^ | 0.674^***^ | 1 |  |  |  |  |  |  |
| 1. Depressive Symptoms 2015 | −0.222^***^ | −0.238^***^ | −0.251^***^ | 1 |  |  |  |  |  |
| 1. Depressive Symptoms 2018 | −0.192^***^ | −0.272^***^ | −0.254^***^ | 0.543^***^ | 1 |  |  |  |  |
| 1. Depressive Symptoms 2020 | −0.222^***^ | −0.255^***^ | −0.301^***^ | 0.537^***^ | 0.576^***^ | 1 |  |  |  |
| 1. Internet Use 2015 | 0.241^***^ | 0.224^***^ | 0.223^***^ | −0.110^***^ | −0.103^***^ | −0.126^***^ | 1 |  |  |
| 1. Internet Use 2018 | 0.268^***^ | 0.290^***^ | 0.273^***^ | −0.134^***^ | −0.132^***^ | −0.140^***^ | 0.423^***^ | 1 |  |
| 1. Internet Use 2020 | 0.337^***^ | 0.367^***^ | 0.397^***^ | −0.146^***^ | −0.136^***^ | −1.690^***^ | 0.295^***^ | 0.408^***^ | 1 |
| Note: * *p* < 0.05, ** *p* < 0.01, *** *p* < 0.001. | | | | | | | | | |

Table S4. Estimated coefficients in Model 4i.

| **Model pathway** | **β*** |  | **SE** |  | **β** |  | **SE** |
| --- | --- | --- | --- | --- | --- | --- | --- |
| **Between-person level** |  |  |  |  |  |  |  |
| Random intercept factors correlation |  |  |  |  |  |  |  |
| CF(T1) → RI_CF_ | 1.000 |  | 0.000 |  | 0.680*** |  | 0.009 |
| CF(T2) → RI_CF_ | 1.000 |  | 0.000 |  | 0.590*** |  | 0.010 |
| CF(T3) → RI_CF_ | 1.000 |  | 0.000 |  | 0.632*** |  | 0.010 |
| DS(T1) → RI_DS_ | 1.000 |  | 0.000 |  | 0.623*** |  | 0.011 |
| DS(T2) → RI_DS_ | 1.000 |  | 0.000 |  | 0.574*** |  | 0.013 |
| DS(T3) → RI_DS_ | 1.000 |  | 0.000 |  | 0.588** |  | 0.012 |
| RI_CF_ ↔ RI_DS_ | -1.209*** |  | 0.166 |  | -0.206*** |  | 0.027 |
| **Within-person level** |  |  |  |  |  |  |  |
| Autoregressive paths |  |  |  |  |  |  |  |
| CF(T1) → CF(T2) | 0.195*** |  | 0.022 |  | 0.153** |  | 0.017 |
| CF(T2) → CF(T3) | 0.195*** |  | 0.022 |  | 0.219*** |  | 0.025 |
| DS(T1) → DS(T2) | 0.146*** |  | 0.024 |  | 0.129*** |  | 0.020 |
| DS(T2) → DS(T3) | 0.146*** |  | 0.024 |  | 0.153*** |  | 0.025 |
| Cross-lagged paths |  |  |  |  |  |  |  |
| CF(T1) → DS(T2) | -0.080*** |  | 0.029 |  | -0.036*** |  | 0.013 |
| CF(T2) → DS(T3) | -0.080*** |  | 0.029 |  | -0.048*** |  | 0.017 |
| DS(T1) → CF(T2) | -0.019** |  | 0.009 |  | -0.029** |  | 0.014 |
| DS(T2) → CF(T3) | -0.019** |  | 0.009 |  | -0.036** |  | 0.018 |
| (Residual) correlations |  |  |  |  |  |  |  |
| CF(T1) ↔ DS(T1) | -0.395*** |  | 0.166 |  | -0.047** |  | 0.020 |
| CF(T2) ↔ DS(T2) | -1.727*** |  | 0.260 |  | -0.148*** |  | 0.022 |
| CF(T3) ↔ DS(T3) | -1.240*** |  | 0.168 |  | -0.130*** |  | 0.017 |

**Note.** Baseline N = 9,610 ; bootstrap replications = 500. β = standardized path coefficients; β* = unstandardized path coefficients; SE = standard error; CF = cognitive function; DS = depressive symptoms; RI = random intercept; T1/T3 = time 1 and time 3. ***p < 0.001, **p < 0.05, *p < 0.1.

Table S5. Estimated Coefficients for Model 5i by Age Subgroup (age 45-64 and age≥65).

| **Model pathway** | **(age45-64)subgroup** | | | | | | |  | **(age≥65)subgroup** | | | | | | |
| --- | --- | --- | --- | --- | --- | --- | --- | --- | --- | --- | --- | --- | --- | --- | --- |
|  | **β*** |  | **SE** |  | **β** |  | **SE** |  | **β*** |  | **SE** |  | **β** |  | **SE** |
| **Between-person level** |  |  |  |  |  |  |  |  |  |  |  |  |  |  |  |
| CF(T1) → RI_CF_ | 1.000 |  | 0.000 |  | 0.686*** |  | 0.012 |  | 1.000 |  | 0.000 |  | 0.698*** |  | 0.021 |
| CF(T2) → RI_CF_ | 1.000 |  | 0.000 |  | 0.568*** |  | 0.011 |  | 1.000 |  | 0.000 |  | 0.576*** |  | 0.020 |
| CF(T3) → RI_CF_ | 1.000 |  | 0.000 |  | 0.609*** |  | 0.012 |  | 1.000 |  | 0.000 |  | 0.600*** |  | 0.021 |
| DS(T1) → RI_DS_ | 1.000 |  | 0.000 |  | 0.572*** |  | 0.014 |  | 1.000 |  | 0.000 |  | 0.578*** |  | 0.022 |
| DS(T2) → RI_DS_ | 1.000 |  | 0.000 |  | 0.532*** |  | 0.014 |  | 1.000 |  | 0.000 |  | 0.563*** |  | 0.022 |
| DS(T3) → RI_DS_ | 1.000 |  | 0.000 |  | 0.547*** |  | 0.014 |  | 1.000 |  | 0.000 |  | 0.559*** |  | 0.021 |
| IU(T3) → RI_IU_ | 1.000 |  | 0.000 |  | 0.387*** |  | 0.026 |  | 1.000 |  | 0.000 |  | 0.520*** |  | 0.113 |
| IU(T3) → RI_IU_ | 1.000 |  | 0.000 |  | 0.302*** |  | 0.022 |  | 1.000 |  | 0.000 |  | 0.348*** |  | 0.081 |
| IU(T3) → RI_IU_ | 1.000 |  | 0.000 |  | 0.217*** |  | 0.015 |  | 1.000 |  | 0.000 |  | 0.212*** |  | 0.049 |
| **Within-person level** |  |  |  |  |  |  |  |  |  |  |  |  |  |  |  |
| Autoregressive paths |  |  |  |  |  |  |  |  |  |  |  |  |  |  |  |
| CF(T1) → CF(T2) | 0.305*** |  | 0.020 |  | 0.225*** |  | 0.015 |  | 0.379*** |  | 0.038 |  | 0.269*** |  | 0.029 |
| CF(T2) → CF(T3) | 0.305*** |  | 0.020 |  | 0.340*** |  | 0.022 |  | 0.379*** |  | 0.038 |  | 0.407*** |  | 0.041 |
| DS(T1) → DS(T2) | 0.187*** |  | 0.024 |  | 0.169*** |  | 0.021 |  | 0.098** |  | 0.041 |  | 0.094** |  | 0.039 |
| DS(T2) → DS(T3) | 0.187*** |  | 0.024 |  | 0.195*** |  | 0.026 |  | 0.098** |  | 0.041 |  | 0.097** |  | 0.041 |
| IU(T1) → IU(T2) | 0.293*** |  | 0.018 |  | 0.222*** |  | 0.015 |  | 0.582*** |  | 0.059 |  | 0.355*** |  | 0.049 |
| IU(T2) → IU(T3) | 0.293*** |  | 0.018 |  | 0.205*** |  | 0.013 |  | 0.582*** |  | 0.059 |  | 0.339*** |  | 0.041 |
| Cross-lagged paths |  |  |  |  |  |  |  |  |  |  |  |  |  |  |  |
| CF(T1) → DS(T2) | -0.200*** |  | 0.026 |  | -0.087*** |  | 0.012 |  | -0.244*** |  | 0.052 |  | -0.113*** |  | 0.025 |
| CF(T2) → DS(T3) | -0.200*** |  | 0.026 |  | -0.123*** |  | 0.017 |  | -0.244*** |  | 0.052 |  | -0.158*** |  | 0.033 |
| CF(T1) → IU(T2) | 0.019*** |  | 0.001 |  | 0.133*** |  | 0.010 |  | 0.006*** |  | 0.002 |  | 0.077*** |  | 0.019 |
| CF(T2) → IU(T3) | 0.019*** |  | 0.001 |  | 0.126*** |  | 0.010 |  | 0.006*** |  | 0.002 |  | 0.064*** |  | 0.015 |
| IU(T1) → CF(T2) | 0.653*** |  | 0.083 |  | 0.053*** |  | 0.007 |  | 0.540** |  | 0.261 |  | 0.019** |  | 0.009 |
| IU(T2) → CF(T3) | 0.653*** |  | 0.083 |  | 0.077*** |  | 0.010 |  | 0.540** |  | 0.261 |  | 0.033** |  | 0.016 |
| DS(T1) → CF(T2) | -0.060*** |  | 0.008 |  | -0.093*** |  | 0.012 |  | -0.035** |  | 0.016 |  | -0.052** |  | 0.024 |
| DS(T2) → CF(T3) | -0.060*** |  | 0.008 |  | -0.114*** |  | 0.014 |  | -0.035** |  | 0.016 |  | -0.058** |  | 0.027 |
| DS(T1) → IU(T2) | -0.002** |  | 0.001 |  | -0.029** |  | 0.011 |  | -0.001* |  | 0.001 |  | -0.034* |  | 0.018 |
| DS(T2) → IU(T3) | -0.002** |  | 0.001 |  | -0.022** |  | 0.008 |  | -0.001* |  | 0.001 |  | -0.021* |  | 0.011 |
| IU(T1) → DS(T2) | -0.371** |  | 0.156 |  | -0.018** |  | 0.007 |  | -1.195** |  | 0.465 |  | -0.027** |  | 0.011 |
| IU(T2) → DS(T3) | -0.371** |  | 0.156 |  | -0.024** |  | 0.010 |  | -1.195** |  | 0.465 |  | -0.044** |  | 0.017 |
| (Residual) correlations |  |  |  |  |  |  |  |  |  |  |  |  |  |  |  |
| CF(T1) ↔ DS(T1) | -1.399*** |  | 0.205 |  | -0.146*** |  | 0.020 |  | -1.542*** |  | 0.416 |  | -0.166*** |  | 0.042 |
| CF(T1) ↔ IU(T1) | 0.078*** |  | 0.008 |  | 0.146*** |  | 0.014 |  | 0.012** |  | 0.006 |  | 0.050** |  | 0.024 |
| DS(T1) ↔ IU(T1) | -0.039*** |  | 0.013 |  | -0.037*** |  | 0.013 |  | -0.022** |  | 0.010 |  | -0.044** |  | 0.020 |
| CF(T2) ↔ DS(T2) | -2.575*** |  | 0.276 |  | -0.198*** |  | 0.022 |  | -1.748*** |  | 0.516 |  | -0.133*** |  | 0.040 |
| CF(T2) ↔ IU(T2) | 0.092*** |  | 0.011 |  | 0.109*** |  | 0.013 |  | 0.034** |  | 0.011 |  | 0.069*** |  | 0.021 |
| DS(T2) ↔ IU(T2) | -0.061*** |  | 0.018 |  | -0.042*** |  | 0.012 |  | -0.043** |  | 0.015 |  | -0.056*** |  | 0.019 |
| CF(T3) ↔ DS(T3) | -1.830*** |  | 0.181 |  | -0.173*** |  | 0.017 |  | -1.794*** |  | 0.369 |  | -0.163*** |  | 0.033 |
| CF(T3) ↔ IU(T3) | 0.114*** |  | 0.014 |  | 0.112*** |  | 0.014 |  | 0.072*** |  | 0.015 |  | 0.100*** |  | 0.021 |
| DS(T3) ↔ IU(T3) | -0.083*** |  | 0.024 |  | -0.046*** |  | 0.013 |  | -0.066** |  | 0.028 |  | -0.051** |  | 0.022 |

**Note.** Baseline N = 9,610; bootstrap replications = 500. β = standardized path coefficients; β* = unstandardized path coefficients; SE = standard error; CF = cognitive function; DS = depressive symptoms; RI = random intercept; T1/T3 = time 1 and time 3. ***p < 0.001, **p < 0.05, *p < 0.1.

As suggested by the reviewers, we have added RI-CLPM model fit indices for the 45-64 and 65+ age groups to the Supplementary Materials. A multi-group analysis demonstrates that both age groups exhibit adequate model fit, reliability, and validity, permitting further exploration of differences between the subgroup models.

This study compared model coefficients using both standardized and unstandardized approaches, following the guidance of Duncan (1975). Specifically, we used unstandardized coefficients for testing the statistical significance of differences between models, as recommended by Duncan. The corresponding formula from Duncan (1975)[[[3]](#footnote-4)]is presented below:


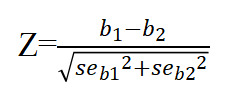


Following this approach, an absolute Z-value greater than 1.96 indicates a significant difference between two unstandardized regression coefficients; otherwise, no significant difference is inferred. Based on this method, we examined if a significant difference existed in the mediating effects of internet use on the cognition-depression pathway between the two age subgroup models.

Table S6. Comparison of path coefficients in RI-CLPM age subgroup models.

1. Fit indices Table

| **Model** | **S-B x2** | **df** | **RMSEA** | **CFI** | **TLI** | **P** |
| --- | --- | --- | --- | --- | --- | --- |
|
| Model 5i, (age45-64)subgroup | 1335.841 | 171 | 0.031 | 0.948 | 0.917 | <0.001 |
| Model 5i, (age≥65)subgroup | 504.028 | 171 | 0.028 | 0.955 | 0.928 | <0.001 |

Note: S-B χ^2^ =Satorra-Bentler Chi-square test statistic; df = degree of freedom; CFI = comparative fit index; TLI = Tucker Lewis index;

RMSEA = root mean square error of approximation.

B.Model Path Coefficients Table（Model 5i Cognition → Depression pathway）

| **Model pathway** | **(age45-64)subgroup** | | |  | **(age≥65)subgroup** | | |  |  |  |
| --- | --- | --- | --- | --- | --- | --- | --- | --- | --- | --- |
|  | **β*** |  | **SE** |  | **β*** |  | **SE** |  | **Z value** | **P-value** |
| Within-person level |  |  |  |  |  |  |  |  |  |  |
| CF(T1)→ DS(T3) | -0.105 |  | 0.012 |  | -0.124 |  | 0.025 |  | - | - |
| CF(T1) → CF(T2)→ DS(T3) | -0.061 |  | 0.009 |  | -0.093 |  | 0.021 |  | - | - |
| CF(T1) → IU(T2)→ DS(T3) | -0.007 |  | 0.003 |  | -0.007 |  | 0.003 |  | - | - |
| CF(T1) → DS(T2)→ DS(T3) | -0.037 |  | 0.005 |  | -0.024 |  | 0.010 |  | - | - |
| CF(T1) → CF(T2) | 0.305 |  | 0.020 |  | 0.379 |  | 0.038 |  | 1.72 | 0.085 |
| CF(T2)→ DS(T3) | -0.200 |  | 0.026 |  | -0.035 |  | 0.016 |  | 5.40 | <0.001 |
| CF(T1) → IU(T2) | 0.019 |  | 0.001 |  | 0.006 |  | 0.002 |  | 5.81 | <0.001 |
| IU(T2)→ DS(T3) | -0.371 |  | 0.156 |  | -1.195 |  | 0.465 |  | 1.68 | 0.098 |
| CF(T1) → DS(T2) | -0.200 |  | 0.026 |  | -0.035 |  | 0.016 |  | 5.40 | <0.001 |
| DS(T2)→ DS(T3) | 0.187 |  | 0.024 |  | 0.098 |  | 0.041 |  | 1.87 | 0.061 |

Note:β,standardized path coefficients; β*,unstandardized path coefficients; SE, standard error,

CF, Cognitive function ; DS, Depressive symptoms; IU, Internet use; T1 ,T3 time 1 and 3.

Considering this high attrition rate, we further assess whether the variables of interest can predict their exit by conducting regression analysis, and report the results in Table S7. As the dependent variable is whether the participates left after a specific survey year, we find that in Column (1), for example, Internet use , and cognitive function level and depression symptoms in 2015 does not predict her leave after 2015. Similar results are found in other columns, indicating that attrition does not significantly correlate with Internet use, cognition and depression, confirming that the issue of sample attrition does not seriously bias the baseline results.

Table s7, Robustness Test for Attrition Bias.

| Explained variables: | 2015-2018 | 2018-2020 | 2015-2020 |
| --- | --- | --- | --- |
| Exiting the sample (yes = 1) | (1) | (2) | (3) |
| N | 9610 | 6502 | 16112 |
| Internet use (0-1) | 0.884  (0.132) | 1.064  (0.153) | 0.965  (0.113) |
| Cognitive Function (0-21) | 1.006  (0.005) | 0.998  (0.008) | 1.001  （0.005） |
| Depressive Symptoms (0-30) | 0.882  (0.009) | 0.891  (0.013) | 0.869  （0.005） |
| Control variables | Yes | Yes | Yes |
| Year effect Control | / | / | Yes |
| Adj. R2 | 0.088 | 0.046 | 0.090 |

*Note : Control variables included gender, age, educational attainment, residence type, marital status, physical activity level, smoking habits, alcohol consumption, instrumental activities of daily living (IADL) scale, and chronic disease status. Robust standard errors are presented in parentheses and are clustered at the individual level. The constant item is not shown; ***p < 0.001, **p < 0.05, *p < 0.1.*

**Section 4**

(Sensitivity analysis)

We incorporated COVID-19 related variables from the CHARLS Wave 5 (2020) dataset into sensitivity analyses (Models 4i and 5i in the manuscript). These variables included: (1) duration of self-isolation during the pandemic outbreak, and (2) self-reported fear or anxiety following the pandemic. Detailed descriptions of these COVID-19 related measures are presented in Table Q1.

Table Q1. Measurement of the COVID-19 related covariates.

| **Variables** | **Score range** | **Sample Characteristic**  **(Wave 5; 2020, N=5883)** |
| --- | --- | --- |
| Days of Longest Self-Isolation During the Pandemic | Four-point scale  0= 0 day; 1= 1-14 days;  2= 15-30 days ; 3= more than 30days. | 0 day, N=2263;  1-14 days, N=1184;  15-30 days, N=1450;  more than 30days, N=986. |
| Feeling Fears During the Lunar New Year Outbreak | Four-point scale  0= Rarely or Never;  1= Not often;  2= Sometimes ; 3= Often Times. | Rarely or Never, N=3458;  Not often, N=638;  Sometimes, N=968;  Often Times, N=819. |
| Feeling Anxiety During the Lunar New Year Outbreak | Four-point scale  0= Rarely or Never;  1= Not often;  2= Sometimes ; 3= Often Times. | Rarely or Never, N=3640;  Not often, N=665;  Sometimes, N=981;  Often Times, N=597. |

Table Q2. Fit indices of RI-CLPM and mediation analysis.

| **Model** | **S-B χ2** | **df** | **RMSEA** | **CFI** | **TLI** | ***p*** |
| --- | --- | --- | --- | --- | --- | --- |
|
| Model Ai, add Covid-19 related variables in Model 4i | 522.289 | 121 | 0.021 | 0.952 | 0.925 | <0.001 |
| Model Bi, add Covid-19 related variables in Model 5i | 957.202 | 189 | 0.024 | 0.947 | 0.917 | <0.001 |

Table Q3. Estimated coefficients in Model Ai.

| **Model pathway** | **β*** |  | **SE** |  | **β** |  | **SE** |
| --- | --- | --- | --- | --- | --- | --- | --- |
| **Between-person level** |  |  |  |  |  |  |  |
| Random intercept factors correlation |  |  |  |  |  |  |  |
| CF(T1) → RI_CF_ | 1.000 |  | 0.000 |  | 0.680*** |  | 0.010 |
| CF(T2) → RI_CF_ | 1.000 |  | 0.000 |  | 0.591*** |  | 0.011 |
| CF(T3) → RI_CF_ | 1.000 |  | 0.000 |  | 0.633*** |  | 0.011 |
| DS(T1) → RI_DS_ | 1.000 |  | 0.000 |  | 0.608*** |  | 0.012 |
| DS(T2) → RI_DS_ | 1.000 |  | 0.000 |  | 0.561*** |  | 0.013 |
| DS(T3) → RI_DS_ | 1.000 |  | 0.000 |  | 0.576** |  | 0.013 |
| RI_CF_ ↔ RI_DS_ | -1.178*** |  | 0.186 |  | -0.206*** |  | 0.032 |
| **Within-person level** |  |  |  |  |  |  |  |
| Autoregressive paths |  |  |  |  |  |  |  |
| CF(T1) → CF(T2) | 0.192*** |  | 0.025 |  | 0.151*** |  | 0.019 |
| CF(T2) → CF(T3) | 0.192*** |  | 0.025 |  | 0.216*** |  | 0.029 |
| DS(T1) → DS(T2) | 0.150*** |  | 0.021 |  | 0.133*** |  | 0.019 |
| DS(T2) → DS(T3) | 0.150*** |  | 0.021 |  | 0.158*** |  | 0.023 |
| Cross-lagged paths |  |  |  |  |  |  |  |
| CF(T1) → DS(T2) | -0.072** |  | 0.033 |  | -0.032** |  | 0.015 |
| CF(T2) → DS(T3) | -0.072** |  | 0.033 |  | -0.043** |  | 0.020 |
| DS(T1) → CF(T2) | -0.018** |  | 0.010 |  | -0.029* |  | 0.016 |
| DS(T2) → CF(T3) | -0.018** |  | 0.010 |  | -0.037* |  | 0.020 |
| (Residual) correlations |  |  |  |  |  |  |  |
| CF(T1) ↔ DS(T1) | -0.408** |  | 0.168 |  | -0.048** |  | 0.019 |
| CF(T2) ↔ DS(T2) | -1.712*** |  | 0.305 |  | -0.146*** |  | 0.026 |
| CF(T3) ↔ DS(T3) | -1.234*** |  | 0.155 |  | -0.133*** |  | 0.016 |

**Note.** Baseline N = 9,610; bootstrap replications = 500. β = standardized path coefficients; β* = unstandardized path coefficients; SE = standard error; CF = cognitive function; DS = depressive symptoms; RI = random intercept; T1/T3 = time 1 and time 3. ***p < 0.001, **p < 0.05, *p < 0.1.

Table Q4. Estimated coefficients in Model Bi.

| **Model pathway** | **β*** |  | **SE** |  | **β** |  | **SE** |
| --- | --- | --- | --- | --- | --- | --- | --- |
| **Between-person level** |  |  |  |  |  |  |  |
| CF(T1) → RI_CF_ | 1.000 |  | 0.000 |  | 0.646*** |  | 0.011 |
| CF(T2) → RI_CF_ | 1.000 |  | 0.000 |  | 0.563*** |  | 0.011 |
| CF(T3) → RI_CF_ | 1.000 |  | 0.000 |  | 0.600*** |  | 0.011 |
| DS(T1) → RI_DS_ | 1.000 |  | 0.000 |  | 0.592** |  | 0.013 |
| DS(T2) → RI_DS_ | 1.000 |  | 0.000 |  | 0.549*** |  | 0.013 |
| DS(T3) → RI_DS_ | 1.000 |  | 0.000 |  | 0.560*** |  | 0.014 |
| IU(T3) → RI_IU_ | 1.000 |  | 0.000 |  | 0.394*** |  | 0.026 |
| IU(T3) → RI_IU_ | 1.000 |  | 0.000 |  | 0.311*** |  | 0.023 |
| IU(T3) → RI_IU_ | 1.000 |  | 0.000 |  | 0.225*** |  | 0.016 |
| **Within-person level** |  |  |  |  |  |  |  |
| Autoregressive paths |  |  |  |  |  |  |  |
| CF(T1) → CF(T2) | 0.213*** |  | 0.026 |  | 0.170*** |  | 0.020 |
| CF(T2) → CF(T3) | 0.213*** |  | 0.026 |  | 0.239*** |  | 0.025 |
| DS(T1) → DS(T2) | 0.151*** |  | 0.022 |  | 0.135*** |  | 0.019 |
| DS(T2) → DS(T3) | 0.151*** |  | 0.022 |  | 0.157*** |  | 0.023 |
| IU(T1) → IU(T2) | 0.311*** |  | 0.021 |  | 0.237*** |  | 0.015 |
| IU(T2) → IU(T3) | 0.311*** |  | 0.021 |  | 0.219*** |  | 0.015 |
| Cross-lagged paths |  |  |  |  |  |  |  |
| CF(T1) → DS(T2) | -0.172*** |  | 0.022 |  | -0.079*** |  | 0.010 |
| CF(T2) → DS(T3) | -0.172*** |  | 0.022 |  | -0.102*** |  | 0.013 |
| CF(T1) → IU(T2) | 0.016*** |  | 0.002 |  | 0.106*** |  | 0.011 |
| CF(T2) → IU(T3) | 0.016*** |  | 0.002 |  | 0.094*** |  | 0.010 |
| IU(T1) → CF(T2) | 0.629*** |  | 0.080 |  | 0.057*** |  | 0.008 |
| IU(T2) → CF(T3) | 0.629*** |  | 0.080 |  | 0.082*** |  | 0.011 |
| DS(T1) → CF(T2) | -0.045*** |  | 0.008 |  | -0.071*** |  | 0.013 |
| DS(T2) → CF(T3) | -0.045*** |  | 0.008 |  | -0.088*** |  | 0.016 |
| DS(T1) → IU(T2) | -0.003*** |  | 0.001 |  | -0.037*** |  | 0.012 |
| DS(T2) → IU(T3) | -0.003*** |  | 0.001 |  | -0.029*** |  | 0.009 |
| IU(T1) → DS(T2) | -0.407*** |  | 0.136 |  | -0.021*** |  | 0.007 |
| IU(T2) → DS(T3) | -0.407*** |  | 0.136 |  | -0.029*** |  | 0.009 |
| (Residual) correlations |  |  |  |  |  |  |  |
| CF(T1) ↔ DS(T1) | -1.071*** |  | 0.155 |  | -0.120*** |  | 0.016 |
| CF(T1) ↔ IU(T1) | 0.069*** |  | 0.007 |  | 0.127*** |  | 0.013 |
| DS(T1) ↔ IU(T1) | -0.034** |  | 0.014 |  | -0.033** |  | 0.014 |
| CF(T2) ↔ DS(T2) | -2.222*** |  | 0.258 |  | -0.188*** |  | 0.022 |
| CF(T2) ↔ IU(T2) | 0.095*** |  | 0.011 |  | 0.117*** |  | 0.013 |
| DS(T2) ↔ IU(T2) | -0.077*** |  | 0.017 |  | -0.055*** |  | 0.012 |
| CF(T3) ↔ DS(T3) | -1.628*** |  | 0.156 |  | -0.172*** |  | 0.016 |
| CF(T3) ↔ IU(T3) | 0.092*** |  | 0.014 |  | 0.099*** |  | 0.015 |
| DS(T3) ↔ IU(T3) | -0.099*** |  | 0.025 |  | -0.060*** |  | 0.015 |

**Note.** Baseline N = 9,610; bootstrap replications = 500. β = standardized path coefficients; β* = unstandardized path coefficients; SE = standard error; CF = cognitive function; DS = depressive symptoms; RI = random intercept; T1/T3 = time 1 and time 3. ***p < 0.001, **p < 0.05, *p < 0.1.

Table Q5. Direct and indirect effects in Model Bi

| **Model pathway** |  | **Effect** | **β** | **SE** | **P value** | **95% CI** |
| --- | --- | --- | --- | --- | --- | --- |
| **Overall sample** |  | (Baseline 2015: N=9610) |  |  |  |  |
| pathway from CF T1 to DS T3 |  | Total effect | −0.033 | 0.005 | <0.001 | -0.042, -0.024 |
| pathway from CF T1 to CF T2 to DS T3 |  | Specific indirect 1 | −0.017 | 0.003 | <0.001 | -0.024, -0.011 |
| pathway from CF T1 to DS T2 to DS T3 |  | Specific indirect 2 | −0.012 | 0.002 | <0.001 | -0.017, -0.008 |
| pathway from CF T1 to IU T2 to DS T3 |  | Indirect effect | **−0.003** | **0.001** | **0.003** | **-0.005, -0.001** |
|  |  |  |  |  |  |  |
| pathway from DS T1 to CF T3 |  | Total effect | −0.032 | 0.006 | <0.001 | -0.044, -0.020 |
| pathway from DS T1 to CF T2 to CF T3 |  | Specific indirect 1 | −0.017 | 0.003 | <0.001 | -0.022, -0.010 |
| pathway from DS T1 to DS T2 to CF T3 |  | Specific indirect 2 | −0.012 | 0.003 | <0.001 | -0.017, -0.006 |
| pathway from DS T1 to IU T2 to CF T3 |  | Indirect effect | **−0.003** | **0.001** | **0.004** | **-0.005, -0.001** |

Note:All effects are standardized, CF, Cognitive function ; DS, Depressive symptoms; IU, Internet use; β, effect: standardized path coefficients, SE, standard error.

Although pandemic-related digital adoption may have inflated internet usage rates in 2020, our sensitivity analyses confirm the robustness of the observed mediation mechanisms. However, these findings likely generalize best to non-crisis contexts, warranting replication in post-pandemic cohorts.

1. [?] Hamaker, E. L. The within-between dispute in cross-lagged panel research and how to move forward. Psychological Methods.2023. Doi:10. 1037/met0000600. [↑](#footnote-ref-2)
2. [?] Wang, J., Li, C., Meng, X., & Liu, D. (2021). Validation of the Chinese Version of the Procrastination at Work Scale. Frontiers in psychology, 12, 726595. [↑](#footnote-ref-3)
3. [?] Duncan, O. D. (1975). Path analysis: Sociological examples (A primer). In H. M. Blalock (Ed.), Causal models in the social sciences (pp. 115-138). Aldine Publishing Company. [↑](#footnote-ref-4)
